# Alignment switching in 3D-printed smectic liquid crystal elastomers

**DOI:** 10.1038/s41467-026-75368-z

**Published:** 2026-07-10

**Authors:** Jin-Hyeong Lee, Kyeong Pyo Kim, Lijie Ding, Michael Li, Min Chan Kim, Kyu Hyun, Ji Hoon Kim, Jan-Michael Y. Carrillo, Suk-kyun Ahn

**Affiliations:** 1 School of Chemical Engineering, Pusan National University, Busan, Republic of Korea; 2 School of Mechanical Engineering, Pusan National University, Busan, Republic of Korea; 3 Neutron Scattering Division, Oak Ridge National Laboratory, Oak Ridge, TN USA; 4 Center for Nanophase Materials Sciences, Oak Ridge National Laboratory, Oak Ridge, TN USA; 5 Department of Electrical and Computer Engineering, University of Maryland, College Park, MD USA; 6 Computational Sciences and Engineering Division, Oak Ridge National Laboratory, Oak Ridge, TN USA; 7 Department of Polymer Science and Engineering, Pusan National University, Busan, Republic of Korea; 8 Research Institute of Industrial Technology, Pusan National University, Busan, Republic of Korea; 9 Research Institute for Convergence of Biomedical Science and Technology, Pusan National University Yangsan Hospital, Yangsan, Republic of Korea

**Keywords:** Polymers, Liquid crystals, Mechanical properties

## Abstract

Extrusion-based additive manufacturing has emerged as a powerful platform for designing shape-morphing materials through controlled orientation. However, existing approaches primarily rely on a single mode of flow-induced alignment, limiting orientation programmability. Herein, we present a direct-ink-writing approach for smectic liquid crystal elastics that exploits two distinct alignment modes within a single ink. The smectic ink exhibits shear- and temperature-dependent orientation switching, enabling molecular alignment either perpendicular or parallel to the print direction. Combined rheological, X-ray, and molecular dynamics analyses reveal that this alignment inversion arises from the preservation or collapse of smectic layers under flow. This reversible switching encodes both contractile and elongational actuation within individual filaments, greatly expanding the design freedom of printed liquid crystal elastomers. We demonstrate 2D and 3D structures with diverse programmed shape transformations, highlighting the potential of this platform for adaptive soft actuators and architected functional materials.

## Abstract (web summary)

Extrusion based additive manufacturing is a powerful tool to design shape morphing material, though it is challenging to program shape morphing. Here the authors report a direct-ink-writing method to control the alignment of liquid crystal mesogens for programmable actuation.

## Introduction

Orientation is a fundamental design principle in biology for programming shape and motion to adapt to environmental changes. For example, in the morphogenesis of plant tissues such as *Aquilegia* and *Arabidopsis*, cell orientation patterns encode anisotropic growth, driving the formation of complex shapes. In animals, the orientation of muscle fibers supports diverse modes of actuation in organs, including elongation and contraction^1–4^. Responsive materials can change their shape or properties in response to external stimuli, enabling the design of synthetic systems that undergo programmed shape transformations and perform specific functions under diverse environmental conditions^5–9^. Analogous to biological systems, precise control of molecular or structural orientation in these materials has been a central strategy for programming morphogenesis and shape transformation. However, achieving this requires sophisticated fabrication techniques^10–14^.

Additive manufacturing (AM), or three-dimensional (3D) printing, has advanced beyond rapid prototyping into a platform for programmable material fabrication^15–19^. Among various AM methods, extrusion-based printing is particularly effective for controlling local orientation, as rheological fields align polymer chains^20^, fibrils^10,11,21^, sheets^22^, or liquid crystals^23,24^ along arbitrary print paths during extrusion. Stimuli-responsive materials such as hydrogels, shape memory polymers, liquid crystal elastomers (LCEs), and their composites have been printed with on-demand shape programmability through voxelised anisotropy^25,26^.

In particular, LCEs have emerged as promising candidates for extrusion-based direct-ink-writing (DIW) because their molecular orientation can be programmed during printing, enabling large and reversible anisotropic deformations^23–26^. LCEs are lightly crosslinked polymer networks containing rod-like mesogens^27–31^. The mesogen orientational order, described by the director ($\hat{n}$), couples to the polymer backbone, enabling reversible contraction along the director and expansion perpendicular to the director through order–disorder transitions^32–39^. In DIW, LC prepolymers are extruded through a nozzle and aligned by shear or extensional forces^26,40^. Subsequent in-situ crosslinking fixes the director orientation, enabling the fabrication of LCE structures with spatially programmed anisotropy. To date, DIW of LCEs has typically relied on nematic inks, in which mesogens exhibit only directional order and align uniformly along the print path regardless of processing conditions^23–26,41,42^. Thus, while this approach is well-established, it is inherently limited to a single actuation mode, namely contraction along the print direction, within each printed filament. Recent studies have demonstrated that printing parameters such as print speed, temperature, extrusion pressure, and nozzle geometry can be used to spatially modulate the degree of director alignment^25,26,43–45^ or to generate through-thickness orientation gradients for bending actuation^46^. Nevertheless, in all these approaches, the director orientation within each filament remains fixed parallel to the print path, and achieving both contractile and elongational responses within a single printed filament has not been realized in DIW.

**Fig. 1:**
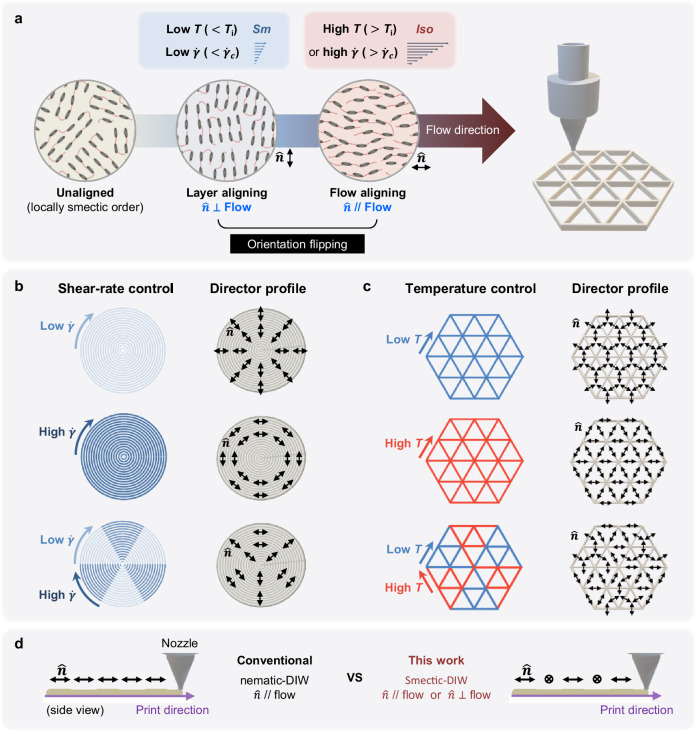
Direct-ink-write 3D printing of smectic LCE. **a** Schematic of the orientation behavior of smectic oligomer ink under flow (left) and DIW-based additive manufacturing (right). In the perpendicular orientation, smectic layers align parallel to the flow direction, and mesogens orient perpendicular to it, while in the parallel orientation, mesogens align along the flow direction. **b** Schematic illustration of shear-rate-controlled printing of LCE disks using an azimuthal print path and the corresponding radial (top), concentric (middle), and mixed (bottom) director profiles. **c** Schematic illustration of temperature-controlled printing of triangular lattice objects with director profiles oriented perpendicular (top) or parallel (middle) to the print path, and a mixed pattern (bottom). **d** Comparison between smectic-DIW (this work) and conventional nematic-DIW.

In this work, we address this limitation by employing the switchable alignment behavior of smectic LCs in DIW. Unlike nematic LCs, smectic LCs, which feature layered structures with directional order, offer two distinct alignment modes: (1) layer alignment in the smectic phase at low shear rates, producing a director orientation perpendicular to the print path, and (2) flow alignment of mesogens once the layers collapse at high temperatures or shear rates, yielding a parallel orientation (Fig. 1a)^47–51^. By tuning printing parameters that govern the shear rate and temperature applied to the ink, smectic DIW enables switching of the director between perpendicular and parallel orientations within a single continuous print path. Given that main-chain LCEs contract parallel and expand perpendicular to the director, this switching provides access to both contractile and elongational shape changes within the same filament. Smectic DIW thus overcomes the limitation of existing DIW strategies, enabling diverse actuation programming within a single print path and complex director patterning within individual filaments or lattice structures that are inaccessible with nematic DIW (Fig. 1b–d).

## Results

### Synthesis and flow behavior of 3D printable smectogenic ink

**Fig. 2:**
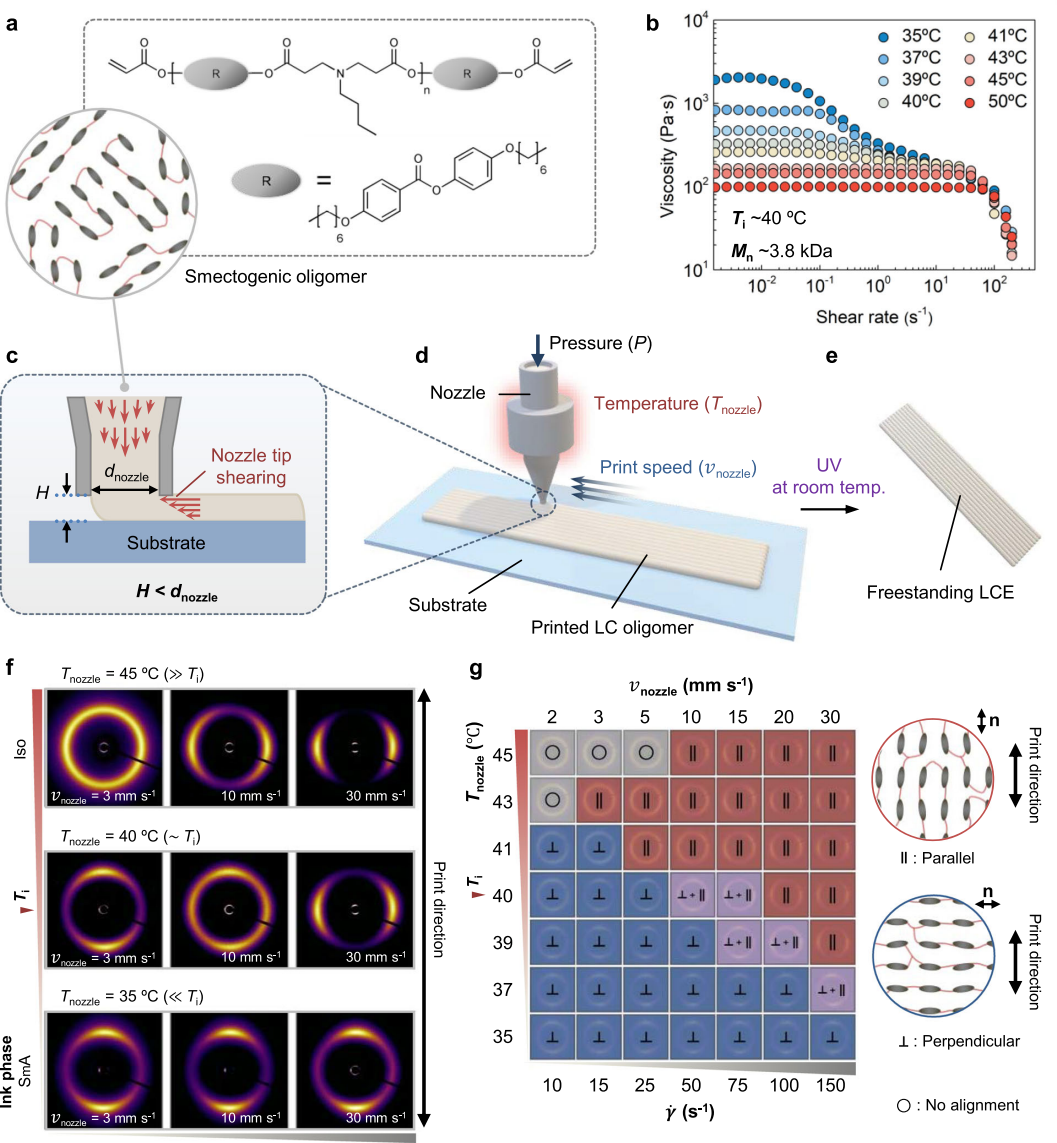
Alignment behavior of 3D-printable smectogenic ink under different printing conditions. **a** Chemical structure of the smectogenic oligomer ink. **b** Shear viscosity as a function of shear rate at various temperatures for the oligomer. **c‒e** Schematic of the direct-ink-write 3D printing of smectic LCEs with varying parameters. *H* denotes the distance between the nozzle tip and substrate (or previous layer), *d*_nozzle_ is the inner diameter of the nozzle, *P*, *T*_nozzle_, and v_nozzle_ are the print pressure, temperature, and speed, respectively. **f** Representative WAXS patterns of LCEs printed at different temperatures and speeds. **g** Mapping diagram of molecular alignment in the print temperature‒shear rate space. The shear rates ($\dot{\gamma }$, bottom axis) were calculated as $\dot{\gamma }$ = v_nozzle_/*H*, using the corresponding v_nozzle_ values shown on the top axis and *H* = 0.2 mm.

To harness the smectic mesophase in DIW, we first prepared a smectogenic oligomer as the printing ink. The oligomer was synthesized following our previously reported protocol via aza-Michael addition between a diacrylate smectogen (C6BAPE) and an amine chain extender (n-butylamine) at a stoichiometric ratio of 1.1:1 (Fig. 2a, Supplementary Fig. 1)^50^. The resulting oligomer integrates smectogens into its backbone, while the acrylate terminal groups enable photopolymerisation after printing, making it directly applicable as a DIW ink (Supplementary Fig. 2). The number average molecular weight of the ink was determined to be 3.8 kDa by ^1^H nuclear magnetic resonance (NMR) spectroscopy and 5.6 kDa by size exclusion chromatography (SEC) (Supplementary Note 1, Supplementary Figs. 2, 3). The thermal transitions of the ink were characterized by differential scanning calorimetry (DSC) (Supplementary Fig. 4). The DSC thermogram shows a glass transition (*T*_*g*_) at −34 °C and a smectic-isotropic transition (*T*_*i*_) at 40 °C with an enthalpic change (Δ*H*) of 12.0 J g⁻^1^ during the second heating cycle. Upon photopolymerization, both *T*_*g*_ and *T*_*i*_ of the resulting smectic LCE increase to −19 °C and 55 °C, respectively, while the associated Δ*H* decreases to 4.8 J g^−^^1^.

The smectic mesophase of the ink affects its rheological behavior under flow as shown by steady-shear measurements (Fig. 2b). The steady shear viscosity (η) of the ink was measured from 35 °C (smectic phase) to 50 °C (isotropic phase), revealing a qualitative change in the shear rate ($\dot{\gamma }$) dependence across the isotropisation temperature, *T*_i_ (40 °C). Below *T*_i_, the viscosity curves (η vs $\dot{\gamma }$) display two distinct shear thinning: the first thinning at low shear rates (0.05$\, < \dot{\gamma } < \,$5), pseudo-Newtonian at intermediate shear rates (5$\, < \dot{\gamma } < \,$40), and the second thinning at higher shear rates ($\dot{\gamma } >$ 40). This double shear-thinning behavior diminishes as the temperature approaches *T*_i_. Above *T*_i_, where the smectic phase completely disappears, the ink exhibits a single shear-thinning regime at high shear rates ($\dot{\gamma } > \,$40) following a long Newtonian plateau, similar to nematic counterparts. The double shear-thinning behavior of LC ink in the smectic phase is attributed to alignment mechanisms analogous to those in layered fluids (e.g., smectic LCPs and lamellar block copolymers)^48^. At low shear rates, the lamellar order is preserved, with layers aligned along the flow and polymer chains oriented perpendicular to it. At high shear rates, the layers are distorted and collapsed, causing the chains to reorient parallel to the flow.

Our smectic DIW strategy aims to pattern the director either perpendicular or parallel to the extrusion path by exploiting the shear- and temperature-dependent alignment behavior of the ink. To examine this orientation controllability, the printing parameters were adjusted to fabricate freestanding rectangular LCE films using linear print paths. Freestanding LCE films were obtained by fixing the printed structures and their alignment through ultraviolet (UV) curing at room temperature. The films were subsequently characterized by X-ray scattering (Fig. 2c–e). Printing was performed using a nozzle with an inner diameter (*d*_nozzle_) of 0.4 mm and a print height (*H*) of ~0.2 mm. Two key parameters, nozzle temperature (*T*_nozzle_ = 35–45 °C) and nozzle translation speed (v_nozzle_ = 2–30 mm s^−1^), were systematically varied, while print pressure and bed temperature were maintained constant at 500 kPa and ~25 °C, respectively (Fig. 2c, d). The printing geometry, where *d*_nozzle_ > *H*, allows the volumetric supply of the ink from the nozzle to exceed the amount that can be carried away by the substrate under the given printing conditions (Supplementary Note 2 and Supplementary Fig. 5). This ensures that director alignment in the printed filament is primarily controlled by shear flow between the nozzle tip and the substrate, rather than by extensional flow or stretching of the extrudate^25,40^. To ensure high fidelity of the printed films, the varying filament widths (*w*) under different printing conditions were compensated by adjusting the infill density (“Methods” and Supplementary Fig. 6).

Wide-angle X-ray scattering (WAXS) measurements were performed to probe the microstructure and orientation of the LCE films printed under varying conditions (Fig. 2f, Supplementary Figs. 7–11). The samples were mounted with the print direction aligned vertically, and X-rays were directed along the surface normal of the films. All WAXS patterns displayed characteristic reflections at *q* ≈ 0.17 Å^−1^, corresponding to the smectic layer spacing of ~3.7 nm (consistent with the molecular length of C6BAPE), and at *q* ≈ 1.43 Å^−1^, corresponding to an inter-mesogen spacing of ~4.4 Å (Supplementary Figs. 10–12). Although smectic layering may be distorted by temperature or shear during printing, the mesogens reassemble into a smectic phase upon cooling to room temperature. Director orientation was analysed from azimuthal scans at *q* ≈ 1.43 Å⁻^1^ (Supplementary Fig. 11). Based on the 2D scattering patterns and scan profiles, four alignment types were identified: (1) perpendicular—arcs along the meridian (director perpendicular to both the surface normal and print direction); (2) parallel—arcs along the equator (director along the print path); (3) isotropic — uniform ring; and (4) mixed—arcs along both meridian and equator, indicating coexistence of perpendicular and parallel orientations. At 35 °C, well below *T*_i_, all printed LCEs exhibited perpendicular alignment with smectic A order, regardless of print speed (2–30 mm s^−1^), suggesting that the alignment is governed by the smectic layers. At 45 °C, well above *T*_i_, the patterns transitioned from isotropic to parallel with increasing print speed, consistent with flow-governed alignment in the absence of smectic layers. Notably, at 40 °C, close to *T*_i_, the director switched from perpendicular to parallel alignment with increasing print speed, and mixed orientations were observed at intermediate speeds (10–15 mm s⁻^1^), marking the critical transition regime. We speculate that the emergence of mixed patterns near the transition regime arises from the slow relaxation dynamics of the LC oligomer compared to low–molecular–weight liquid crystals. In this regime, the orientation distribution appears as a coexistence of perpendicular and parallel alignment rather than a purely discrete orientation.

To further elucidate this transition, the alignment states obtained from WAXS measurements were mapped as a function of print temperature and speed (Fig. 2g). The shear rates ($\dot{\gamma }$) applied during the print process on the x-axis were estimated using a simple Couette flow approximation:

1 $$\begin{array}{c}\dot{\gamma }=\frac{{{\rm{velocity}}}\; {{\rm{difference}}}\; {{\rm{between}}}\; {{\rm{plates}}}}{{{\rm{distance}}}\; {{\rm{seperating}}}\; {{\rm{plates}}}}=\frac{{v}_{{nozzle}}}{H}\end{array}$$

In this study, *H* = 0.2 mm; therefore, v_nozzle_ =[2, 3, 5, 10, 15, 20, 30] mm s^‒1^ generates shear rates of $\dot{\gamma }$ = [10, 15, 25, 50, 75, 100, 150] s^‒1^, respectively.

As shown in Fig. 2g, orientation flipping with varying print speed occurs only when the print temperature is close to *T*_i_. In contrast, at temperatures well below *T*_i_ (35 and 37 °C), the films retain perpendicular alignment across the entire speed range without orientation flipping. This behavior suggests that increasing temperature weakens the smectic order, thereby lowering the critical shear rate required to disrupt the layered structure^48^. We further examined the rows at *T*_nozzle_ = 39 and 40 °C and the columns at v_nozzle_ = 10 and 15 mm s^−1^ ($\dot{\gamma }$ = 50 and 75 s^−1^) in the mapping diagram. These data reveal that near *T*_i_, the director orientation can be switched between perpendicular and parallel either by adjusting the print speed at a fixed temperature or by tuning the print temperature within the intermediate speed range (10–15 mm s^−1^). In particular, the estimated shear-rate range of 50–75 s⁻^1^, corresponding to the orientation-transition region, closely matches the onset of the second shear-thinning regime in the steady-shear viscosity curve ($\dot{\gamma }\approx$ 40–60 s^−1^). This highlights that the shear-induced orientation flipping in the smectic phase is strongly associated with this second shear-thinning region. The optimized conditions described above were employed to print programmable shape morphing actuators, which are discussed later.

### NEMD simulations of mesogen alignment behavior

**Fig. 3:**
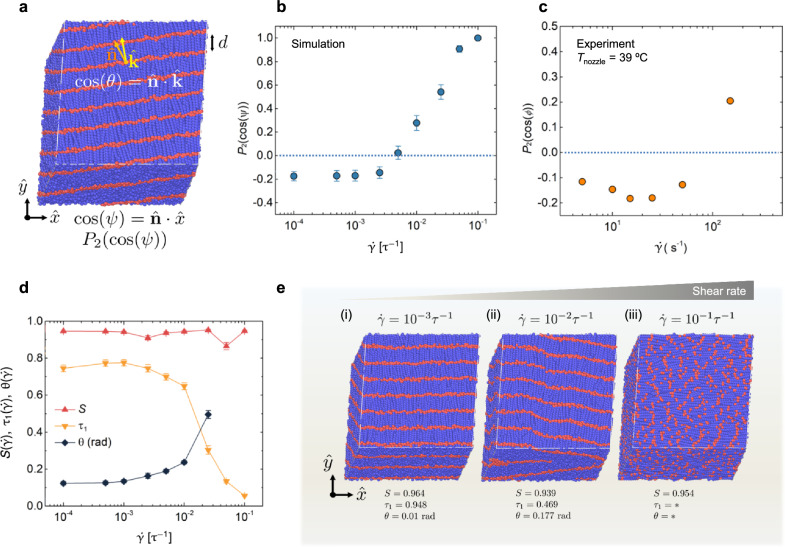
NEMD simulations of mesogen alignment behaviors in the smectic-A phase under shear. **a** System setup showing the mesogen with red bead ends and blue interior beads. The director is denoted by $\hat{{{\bf{n}}}}$, the layer normal vector is shown by $\hat{k}$, θ is the angle between $\hat{{{\bf{n}}}}$ and $\hat{{{\boldsymbol{k}}}}$, ψ is the angle between $\hat{{{\bf{n}}}}$ and the flow direction $\hat{x}$, and d is the distance between layers. **b** Average ${P}_{2}\left(\cos \left(\psi \right)\right)$ as a function of shear rate $\dot{\gamma }$, obtained from approximately 100 independent simulation. Error bars denote the standard deviation. **c** ${P}_{2}\left(\cos \left(\phi \right)\right)$ as a function of shear rate calculated from WAXS experiment. **d** Average $S\left(\dot{\gamma }\right)$, ${\tau }_{1}\left(\dot{\gamma }\right)$ and $\theta \left(\dot{\gamma }\right)$ as a function of shear rate, obtained from approximately 100 independent simulations. Error bars denote the standard deviation. **e** Selected snapshots of the system under shear deformation with shear rates of $\dot{\gamma }=\left[{10}^{-4},{10}^{-3},{10}^{-2}\right]\,{{{\rm{\tau }}}}^{-1}$. The values of S, ${\tau }_{1}$ and θ are indicated, with an asterisk (*) denoting cases where ${\tau }_{1}$ and θ are not reported because the system is no longer in the smectic-A phase. The flow, velocity gradient, and vorticity directions correspond to $\hat{x}$, $\hat{z}$ and $\hat{y}$, respectively.

Capturing the instantaneous reorientation of mesogens during shear flow (or printing) is experimentally challenging, as X-ray measurements only reveal the final structure after cooling and curing. To elucidate the molecular mechanism underlying the observed orientation flipping, we performed non-equilibrium molecular dynamics (NEMD) simulations (Fig. 3). Simulation details are provided in the “Methods” and Supplementary Note 3. To reproduce the shear rate–dependent orientation transition near *T*_i_, smectic-A forming mesogens were modeled as eight connected Lennard-Jones (LJ) beads, following the approach developed by Milchev^52^. In this model, the transition from smectic-A to nematic at shear rates above a critical threshold is primarily driven by entropy, while enthalpic interactions play a minimal role. Prior to NEMD runs, equilibrium MD confirmed the formation of the smectic-A phase, characterized by the translational order parameter (*τ*₁), orientational order parameter (*S*), and layer tilt angle (*θ*) (Fig. 3a, Supplementary Fig. 13). NEMD simulations were then conducted by applying simple shear deformation at different shear rates ($\dot{\gamma }$), where the flow, velocity gradient, and vorticity oriented along the $\hat{{{\bf{x}}}}$, $\hat{{{\bf{z}}}}$, and $\hat{{{\bf{y}}}}$ axes, respectively. The mesogen orientation under shear was quantified using the second-order Legendre polynomial,

2 $${P}_{2}(\cos \psi )=1/2({3\cos }^{2}\psi -1),$$

where *ψ* is the angle between the director and the flow ($\cos \left(\psi \right)=\hat{{{\bf{n}}}}\cdot \hat{{{\bf{x}}}}$). *P*₂ ranges from –0.5 (all mesogens perpendicular to the flow direction) to 1.0 (all mesogens parallel to the flow direction).

As shown in Fig. 3b, the averaged *P*₂ from independent simulations initially remained near –0.2, increased sharply once $\dot{\gamma }$ > 10^−3^ τ^−1^, and approached ≈ 1.0 at $\dot{\gamma }$ = 10^−1^, indicating a shear-induced orientation flip. Distribution analysis revealed that below the critical shear rate ($\dot{\gamma }$*), most domains retained perpendicular alignment (*P*_2_ ≈ –0.5), whereas the distribution inverted at $\dot{\gamma }$ = 10^−2^ τ^−1^, with the majority aligning parallel (*P*_2_ ≈ 1.0) (Supplementary Fig. 14). These simulated trends are consistent with the experimental *P*_2_ values extracted from WAXS measurements (Fig. 2g), confirming the correspondence between print speed and shear rate in governing orientation switching (Fig. 3c).

We further analysed the NEMD simulation trajectories to evaluate the orientational order parameter *S*($\dot{\gamma }$), smectic translational order parameter *τ*₁($\dot{\gamma }$), and layer tilt angle *θ*($\dot{\gamma }$) (Fig. 3d). The orientational order *S*, obtained from the largest eigenvalue of the Q-tensor, remained ≈ 0.9 across all shear rates, indicating that the mesogens maintained strong orientational correlation under all conditions. Moreover, *τ*₁ was calculated from the first Fourier component of the density profile^53,54^. It was ≈ 0.75 at $\dot{\gamma }$ ≤ 10^−2^ τ^−1^, indicating well-defined smectic layering, but decreased toward zero at $\dot{\gamma }$ > 10^−2^ τ^−1^, signifying the collapse of smectic order (i.e., a smectic–nematic transition), accompanied by the reorientation of mesogen from perpendicular to parallel alignment. The distributions of *τ*₁ at selected shear rates ($\dot{\gamma }$ = 10⁻⁴, 10^−3^, and 10^−2^ τ^−1^) are presented in Supplementary Fig. 15. This transition was accompanied by pronounced layer undulations, evidenced by an increase in *θ*, the angle between the layer normal and the director.

Collectively, below the critical shear rate ($\dot{\gamma }$ ≈ 10^−2^ τ^−1^), the smectic layers slide past one another, while the directors remain predominantly perpendicular to the flow, as reflected by high *τ*₁ and *P*₂(cos *ψ*) ≈ –0.2. Above this threshold, the layers become distorted and break down (*θ* increases), the directors realign along the flow (*P*₂(cos *ψ*) approaches 1), and *τ*₁ decays toward zero, marking a shear-induced smectic-to-nematic transition. As shown in Fig. 3e, this evolution is illustrated using representative simulation snapshots (well-ordered layers at $\dot{\gamma }$ = 10^−3^ τ^−1^, undulations near $\dot{\gamma }$ = 10^−2^ τ^−1^, and complete loss of layering at $\dot{\gamma }$ = 10^−1^ τ^−1^). This trend is consistent with previous observations of shear-induced orientation flipping in smectic and other layered fluids^55^.

2D scattering functions in the flow–vorticity plane and viscosity plots as a function of shear rate are obtained from NEMD simulations (Supplementary Figs. 16, 17). Detailed descriptions of the simulations are provided in Supplementary Note 3. The simulation results qualitatively capture the key experimental trends observed in WAXS and rheology measurements: the shear-rate-dependent transition from perpendicular to mixed and finally to parallel orientation relative to the flow direction, and the corresponding double shear-thinning behavior. Although the NEMD simulations operate at spatial and temporal scales different from those of the experiments, they provide qualitative mechanistic insight into how the rheological behavior of the smectic ink, particularly the double shear-thinning response, governs the shear-rate-dependent director switching observed during printing.

### Programming actuation in 3D-printed smectic LCEs

Reversible thermal actuation in LCEs, specifically the actuation mode (contraction or elongation) and strain magnitude, is governed by the director orientation and the degree of alignment, which is commonly quantified by order parameters. In conventional nematic ink–based DIW, the actuation strain can be tuned by controlling the magnitude of molecular orientation along the print path. However, fully reversing the actuation direction from contraction to elongation remains challenging, as the nematic director typically aligns parallel to the extrusion path^23–26,41,42^.

**Fig. 4:**
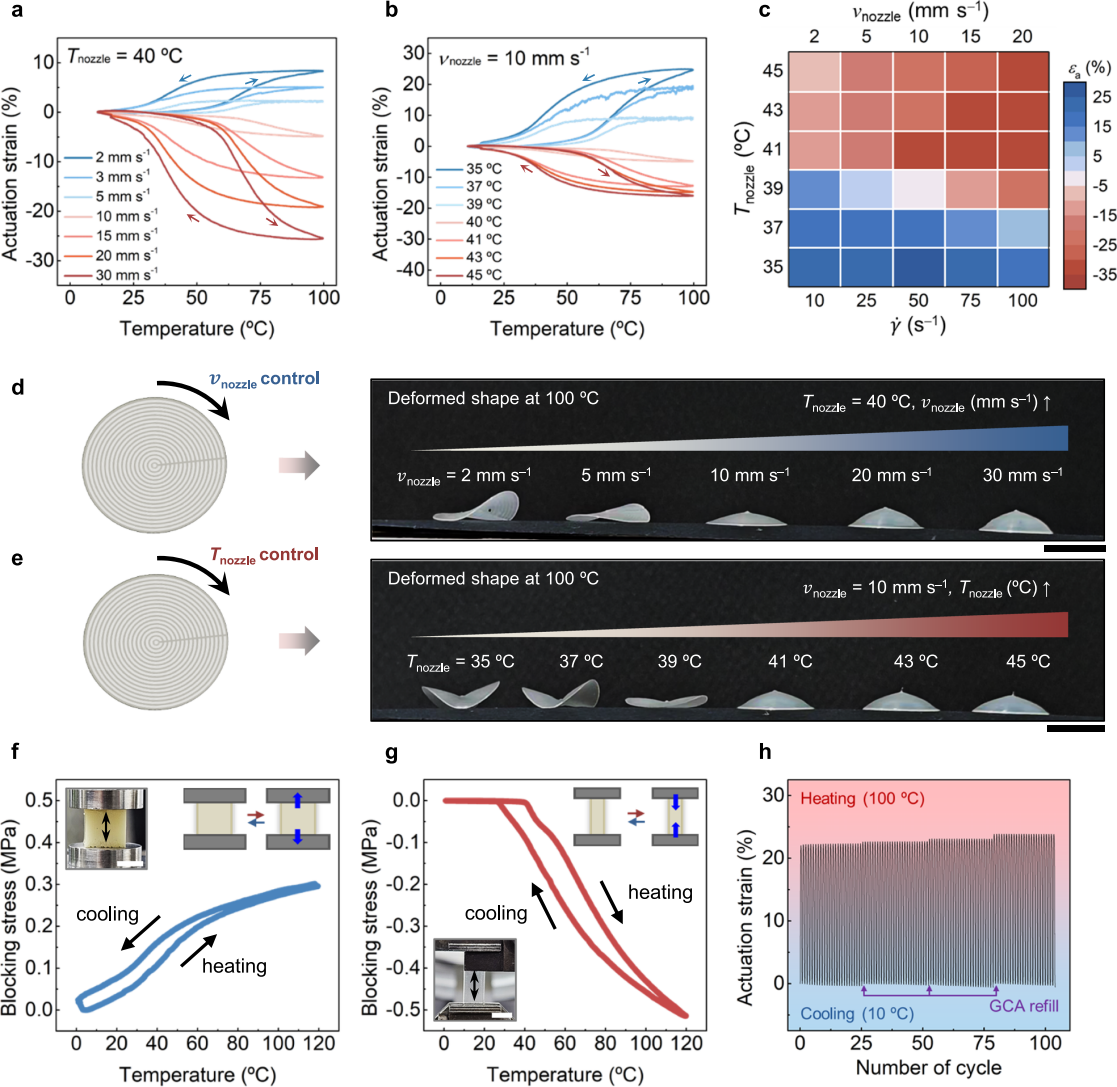
Actuation properties of 3D-printed smectic LCEs under different printing conditions. **a**,** b** Reversible actuation during heating-cooling cycles for samples printed at *T*_nozzle_ = 40 °C with varying v_nozzle_ (**a**), and at v_nozzle_ = 10 mm s^−1^ with different *T*_nozzle_ (**b**), measured by dynamic mechanical analyser. Actuation strain (%) = $(L(T)-{L}_{0})/{L}_{0}\times 100$, where $L\left(T\right)$ is the longitudinal film length at temperature *T*, and ${L}_{0}$ is the initial length. Negative and positive values denote thermotropic contra**c**tion and elongation, respectively. **c** Heat map of actuation strain at 100 °C for filament printed at varying temperatures (*T*_nozzle_, left axis) and nozzle translation speeds (v_nozzle_, top axis), measured by optical microscope. The corresponding shear rates ($\dot{\gamma }$, bottom axis) were calculated as $\dot{\gamma }$ = v_nozzle_/*H*, where *H* = 0.2 mm. **d**,** e** Programmable shape deformation of smectic LCEs printed along an azimuthal circular path by varying v_nozzle_ at fixed *T*_nozzle_ (**d**) or varying *T*_nozzle_ at fixed v_nozzle_ (**e**). Scale bars, 1 cm. **f**,** g** Blocking stress during elongational and contractile actuation as a function of temperature for samples printed at *T*_nozzle_ = 35 °C, v_nozzle_ = ^1^0 mm s⁻^1^ and *T*_nozzle_ = 45 °C, v_nozzle_ = 20 mm s⁻^1^, respectively. Insets show the printed samples; bidirectional arrows indicate the print direction. Scale bars, 1 cm. **h** Actuation stability of the LCE film printed at *T*_nozzle_ = 35 °C and *v*_nozzle_ = 3 mm s^‒1^ over 100 heating–cooling cycles. Liquid nitrogen was refilled into the gas cooling accessory (GCA) before the 26th, 53rd, and 78th cycles.

Based on the print parameter–director relationships established for smectic DIW (Fig. 2g), we demonstrated programmable actuation of LCEs in both strain and direction. Dimensional changes of films printed at *T*_nozzle_ = 40 °C with varying v_nozzle_, and at v_nozzle_ = 10 mm s^−1^ with varying *T*_nozzle_, were monitored during thermal cycling (Fig. 4a, b). The resulting actuation curves reveal graded maximum strains ranging from +8.4% to –25.7% with increasing print speed at 40 °C, and from +25.0% to –16.1% with varying print temperature at 10 mm s^−1^. A heat map of actuation strain, constructed from temperature-controlled optical microscopy measurements of single filaments printed under different conditions (Supplementary Fig. 18, Supplementary Table 1), reveals its dependence on *T*_nozzle_ (left axis) and applied shear rates, $\dot{\gamma }$ (bottom axis) (Fig. 4c). It highlights the transition between elongation (blue) and contraction (red), with color intensity indicating strain magnitude. The actuation direction distribution closely matches the alignment map derived from WAXS (Fig. 2g), confirming that shape morphing is achieved by programming the director in smectic DIW. By adjusting *T*_nozzle_ (35–45 °C) and v_nozzle_ (2–20 mm s^–1^), the actuation response of a single filament can be graded from +28% elongation to −31% contraction, indicating that smectic DIW provides broader and more flexible control over actuation behavior than conventional nematic DIW and fiber-extrusion approaches^25,50^. For example, modulating the printing parameters along the same concentric print path allows the same printed material to exhibit contrasting deformation modes—saddle-like (elongation) and cone-like (contraction) (Fig. 4d, e).

Shear rate and temperature are the primary parameters regulating alignment and thermal actuation, both of which are closely associated with the steady-shear viscosity behavior of the ink. To validate the cross-platform generality of our approach, we further examined the effects of *d*_nozzle_, *H*, and ink viscosity on alignment and actuation behavior. Supplementary Fig. 19 presents actuation strain heat maps as a function of v_nozzle_ and *H* for two nozzle diameters (0.4 and 0.6 mm) under constant conditions of *T*_nozzle_ = 40 °C and *P* = 500 kPa. Regardless of *d*_nozzle_, increasing *H* shifted the critical v_nozzle_ required for orientation switching to proportionally higher values, consistent with the Couette flow approximation, $\dot{\gamma }$ = v_nozzle_ /*H*. When expressed in terms of $\dot{\gamma }$, the perpendicular-to-parallel director transition occurred within a consistent range of 33–67 s^−1^ across all tested v_nozzle_ and *H* combinations, in good agreement with the onset of the second shear-thinning regime in the steady-shear viscosity curve. These results confirm that shear-induced director inversion is governed by the applied $\dot{\gamma }$ rather than by v_nozzle_ or *H* individually, establishing the cross-platform generality of our processing principle.

To examine the effect of ink viscosity, we synthesized a smectogenic ink with the same composition and *T*_i_ but a higher molecular weight (*M*_n_ ≈ 5.0 kDa) than the original ink (*M*_n_ ≈ 3.8 kDa) (Supplementary Figs. 20, 21). As shown in Supplementary Fig. 21, the steady-shear viscosity increased approximately 4–5-fold, and the double shear-thinning behavior shifted to lower shear rates. In the smectic phase, the viscosity curve showed initial shear thinning at low shear rates (0.03$\, < \dot{\gamma } < \,\,$ s^−1^), a pseudo-Newtonian regime at intermediate rates (1$\, < \dot{\gamma } < \,$1  s^−1^), and a second shear-thinning regime at higher rates ($\dot{\gamma } >$ 10 s^−1^). Accordingly, the critical shear rate for alignment switching shifted to lower values (10–25 s^‒1^) relative to that of the original ink (50–75 s^‒1^), as reflected in the actuation strain heat map (Supplementary Fig. 21). This shift is attributed to the longer polymer chains, which impose greater constraints on interlayer slipping and render the smectic layers more susceptible to shear-induced disruption, leading to second shear thinning at lower shear rates^48,49^. Taken together, these results demonstrate that the processing window for switchable alignment can be predicted from the relationship between the applied shear rate and the steady-shear viscosity profile, providing a generalizable design principle for shear-rate-controlled director programming.

The actuation performance of the printed smectic LCEs was evaluated using a dynamic mechanical analyser (Fig. 4f–g, Supplementary Fig. 22). Compression- and tensile-mode isostrain tests were conducted on representative elongating and contracting actuators printed at *T*_nozzle_ = 35 °C, v_nozzle_ = 10 mm s^−1^ and *T*_nozzle_ = 45 °C, v_nozzle_ = 20 mm s^−1^, revealing blocking stresses of +0.30 MPa and −0.51 MPa, respectively. The cyclic stability of the printed LCE was assessed under an isostress mode using a representative elongating LCE printed at *T*_nozzle_ = 35 °C and v_nozzle_ = 3 mm s^−1^ (Fig. 4h, Supplementary Fig. 23). The printed LCE was subjected to 100 thermal actuation cycles over 10–100 °C, during which the actuation strain remained stable, showing only a marginal change of approximately 1.5% and no significant performance degradation.

### Shape programming via print temperature control

DIW of smectic inks enables director orientation to be programmed either perpendicular or parallel to the extrusion path by controlling printing temperature or speed. This allows complex director profiles to be generated from simple rectilinear and azimuthal paths without altering the print path. Furthermore, director patterning can be achieved within a single filament or lattice structure, which is inaccessible using DIW with nematic LCEs.

**Fig. 5:**
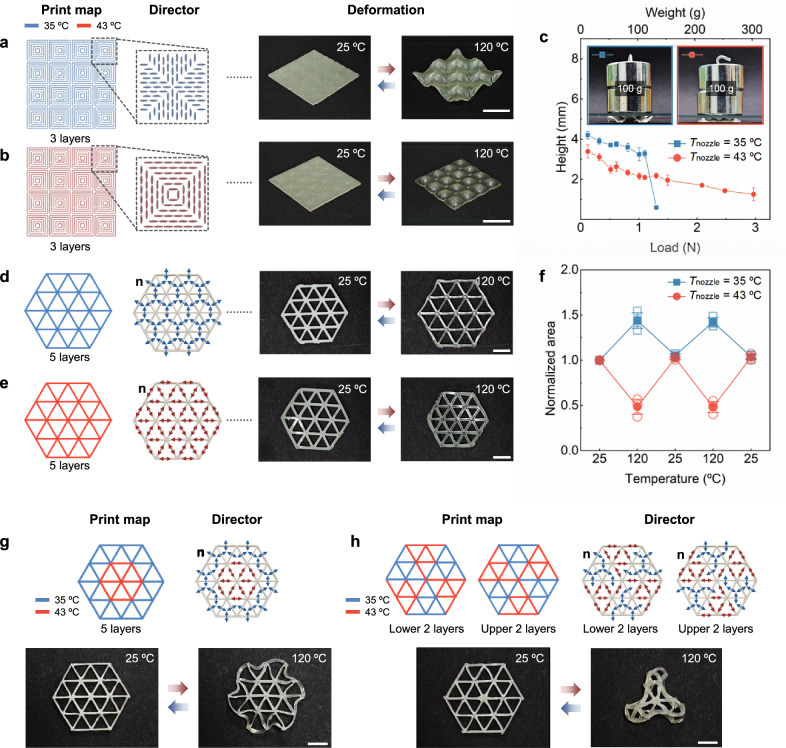
Programming shape transformation via print temperature control. **a**,** b** 4 × 4 concentric square arrays with 3 layers printed at *T*_nozzle_ = 35 °C (**a**) and *T*_nozzle_ = 43 °C (**b**) at v_nozzle_ = 10 mm s^−1,^ producing egg-crate and multiple-cone deformations at 120 °C, respectively. Scale bar, 1 cm. **c** Load-bearing measurements of the deformed structures on a 120 °C hot plate. Error bars represent SDs (*n* = 3). **d**,** e** Triangular lattice structures with 5 layers printed at *T*_nozzle_ = 35 °C (**d**) and *T*_nozzle_ = 43 °C (**e**) at v_nozzle_ = 10 mm s^−1^, showing expanding and contracting actuation at 120 °C, respectively. Scale bar, 4 mm. **f** Normalized pore area variation of the lattice structures in response to temperature changes. Hollow markers represent individual data points, solid markers indicate the mean values, and error bars denote SDs (*n* = 3). **g**,** h** Morphing of spatially programmed triangular lattices into gear-like (**g**) and boomerang-like (**h**) shapes at 120 °C. Scale bar, 4 mm. Paths printed at *T*_nozzle_ = 35 °C and *T*_nozzle_ = 43 °C are represented in blue and red, respectively. Director profiles are depicted as rods or bidirectional arrows.

We first employed temperature-dependent orientation control in smectic DIW to demonstrate programmable thermal transformations. Multilayered LCE structures were fabricated via layer-by-layer printing (Supplementary Fig. 24). At temperatures well below and above *T*_i_, a moderate speed of 10 mm s^−1^ yielded well-defined perpendicular and parallel director orientations, corresponding to elongational and contractile thermal actuation, respectively. Three-layer 4 × 4 concentric square arrays printed at *T*_nozzle_ = 35 °C and 43 °C transformed into egg-crate and multi-cone morphologies upon heating, exhibiting distinct load-bearing behaviors (Fig. 5a–c, Supplementary Fig. 25 and Movie 1–2). The egg-crate structure supported 112.6 g (≈ 266 × its own weight) while maintaining a height of over 3 mm before collapse, whereas the multi-cone structure lifted 302.6 g (≈ 887 × its own weight) while maintaining a height of more than 1 mm. These results demonstrate that programmed topographic deformations generated via multilayer printing can withstand substantial loads, offering opportunities for applications such as reconfigurable surfaces and adaptive topographical structures^56^.

Similarly, five-layer triangular lattices printed at 35 °C and 43 °C exhibited significantly different temperature-responsive pore area changes (approximately +44% and –52%, respectively) (Fig. 5d, f, Supplementary Fig. 25, and Movie 3). Two distinct actuation modes can be spatially integrated within the same lattice, producing more complex thermal deformations. For example, a lattice with an inner contracting region and an outer expanding region transformed from a hexagonal to a gear-like shape (Fig. 5g, Supplementary Movie 4). Spatial programming of opposing actuation modes across layers enabled out-of-plane bending driven by interlayer deformation mismatches. A triangular lattice with alternating upper elongating/lower contracting and upper contracting/lower elongating layers deformed into a boomerang-like shape due to the mismatch between opposing actuation directions (Fig. 5h, Supplementary Movie 5). Together, these results demonstrate the versatility of smectic DIW in achieving spatially programmable, multifunctional actuation using a single material platform.

### Shape programming via print speed control

Temperature modulation during printing may limit process continuity, as thermal equilibration, particularly cooling, is inherently time-consuming. Print speed modulation (i.e., shear-rate modulation), by contrast, enables nearly instantaneous director switching without time lag, making it a more practical approach for continuous and scalable fabrication (Supplementary Movie 6).

**Fig. 6:**
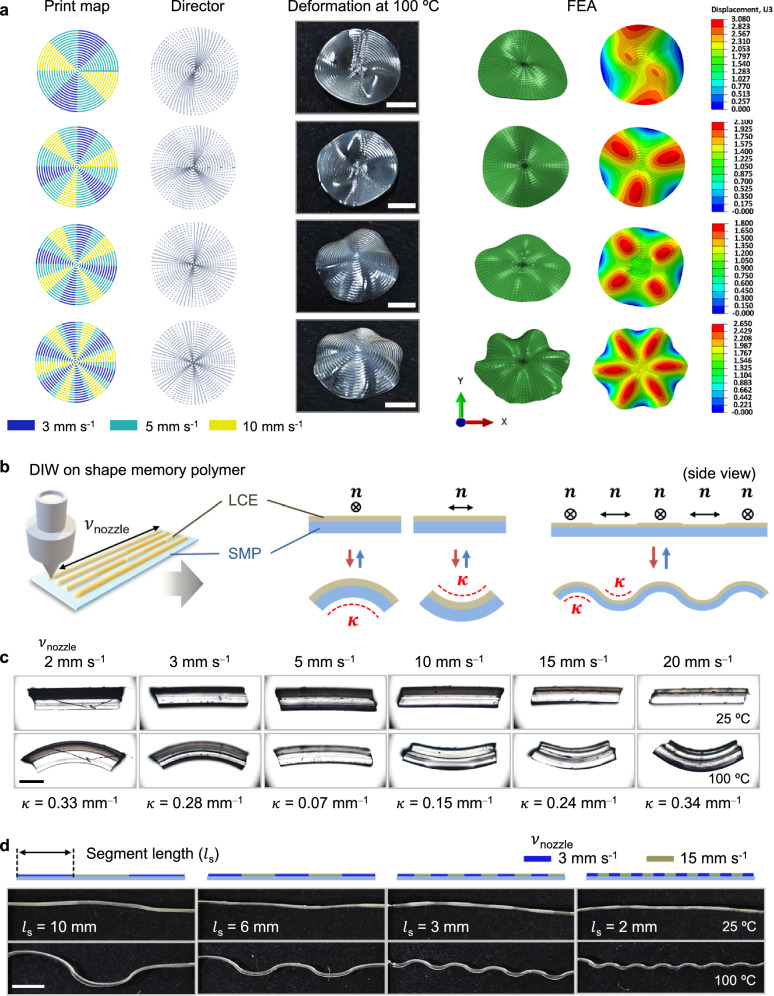
Programming shape transformation via shear rate control. **a** Print paths and speed maps of circular disks (15 mm in diameter), corresponding director profiles, actuated shapes, and finite element analysis (FEA). Thermal deformation images were captured by placing samples on a 100 °C hot plate. Scale bar, 5 mm. FEA results include deformed shapes (left) and vertical displacement contour maps (right). **b** Schematic illustration of shear rate-controlled DIW of smectic LCEs onto a SMP substrate, poly(*tert*-butyl acrylate-*co*-*n*-butyl acrylate). Double arrows indicate the director ($\vec{n}$). **c** Optical microscope images showing side views of bilayer filament segments at 25 °C (unactuated) and 100 °C (actuated), printed at different v_nozzle_. The curvature, κ (mm^−1^), is defined as 1/*R*, where *R* is the radius of curvature. Scale bar, 500 μm. **d** Optical images showing side views of bilayer filaments at 25 °C (unactuated) and 100 °C (actuated), printed with alternating v_nozzle_ of different periodicities. Scale bar, 5 mm.

We demonstrated programmable topographic morphing in LCEs by varying print speeds along azimuthal directions within a single concentric circular layer (Fig. 6a, Supplementary Fig. 26). Speed-induced orientation switching at *T*_nozzle_ = 40 °C was achieved using v_nozzle_ = 3, 5, and 10 mm s^−1^, yielding maximum actuation strains of approximately +5%, +2%, and –5%, respectively, while maintaining comparable filament widths (Fig. 4a, Supplementary Fig. 6). Upon heating to 100 °C, disks printed with azimuthal speed variations exhibited localized morphing, forming valleys and dimples that originated from regions of radial and concentric alignment, respectively (Fig. 6a). Finite element analysis (FEA) of thermal deformation (rightmost column of Fig. 6, Supplementary Fig. 27) reproduced the experimentally observed vertical displacement fields, suggesting good agreement between simulation and experiment results (see “Methods” and Supplementary Note 4 for details).

To further highlight the programming capability of shear rate-induced orientation control of in smectic DIW, we printed smectic LCE filaments onto a flexible shape memory polymer (SMP) substrate, consisting of poly(*tert*-butyl acrylate-*co*-*n*-butyl acrylate), at different v_nozzle_ values at a constant temperature of 40 °C (Fig. 6b–d, Supplementary Fig. 28). The resulting bilayers underwent thermal deformation into either convex or concave geometries with tunable curvature (κ ≈ 0.1–0.3 mm^–1^) to accommodate the strain mismatch between the passive SMP layer and the active LCE layer, whose elongation or contraction was programmed by varying v_nozzle_. (Fig. 6c). Moreover, by alternating v_nozzle_ between low and high values along a single filament, periodic undulations with tunable wavelength could be generated (Fig. 6d). Using a 0.4 mm-diameter nozzle, local segments as short as 1 mm with distinct director orientations could be programmed within a single printed filament (Supplementary Fig. 29). This spatial resolution of director programming is expected to depend on nozzle geometry and ink properties.

### Switchable surface topography actuators

**Fig. 7:**
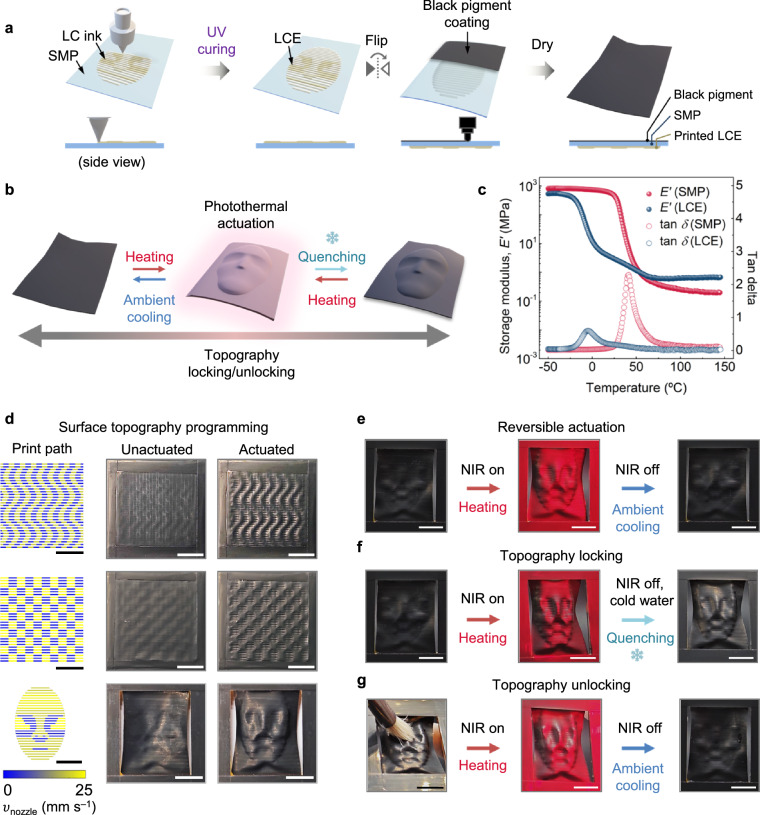
Reversibly switchable surface-topography actuator fabricated via shear rate-controlled DIW of smectic LCEs onto SMP substrates. **a** Schematic illustration of the fabrication process. Smectic LC filaments were printed onto SMP substrates at varying print speeds at 40 °C and subsequently UV-cured for 10 min. The opposite side of the printed surface was then coated with a black pigment to enable photothermal actuation and dried for 10 min under ambient conditions. **b** Schematic illustration of reversible surface-topography switching of the actuator, arising from the actuation of the LCE layer and the shape-fixing capability of the SMP layer. **c** Viscoelastic properties of the smectic LCE and SMP measured by DMA. **d** Photographs of programmed surface-topography patterns, including wavy stripes (top), golf ball dimples (middle), and a human face (bottom), generated from rectilinear print paths with spatially encoded print speeds. Scale bars, 1 cm. **e‒g** Snapshots of the transformable topography actuator programmed with a human-face pattern, showing reversible actuation (**e**), topography locking (**f**), and topography unlocking (**g**). Scale bars, 1 cm.

Finally, we fabricated reversibly switchable surface topography actuators by integrating smectic LCE filaments with a SMP substrate (Fig. 7). The LCE filaments were printed onto SMP substrates and coated with a black pigment to enable photothermal actuation (Fig. 7a). The SMP layer exhibits an abrupt modulus change from ≈470 MPa at 25 °C to ≈0.3 MPa at 100 °C around its glass transition temperature (*T*_g_ ≈ 41 °C). In contrast, the LCE layer shows a more gradual modulus transition over the same temperature range, decreasing from 3 MPa at 25 °C to ≈0.7 MPa at 100 °C (Fig. 7c). In the bilayer configuration, the LCE governs the mechanical response above *T*_i_, whereas the SMP dominates at room temperature (*T* < *T*_g_). As a result, the bilayer is sufficiently compliant to undergo large actuation above *T*_i_, yet becomes rigid upon cooling to room temperature. We exploit this temperature-dependent mechanical contrast between the two layers to achieve shape locking upon rapid quenching and shape unlocking upon ambient cooling (Fig. 7b)^57^.

Rectilinear print paths were encoded with spatially varying v_nozzle_ values to program distinct director profiles section by section (left panels, Fig. 7d). Upon near-infrared (NIR) irradiation, the printed structures exhibited preprogrammed surface topographies, including wavy stripes, golf ball dimples, and a human face, and returned to their initial flat state upon ambient cooling (middle and right panels, Fig. 7d, Supplementary Movie 7). This actuation is fully reversible across multiple cycles (Fig. 7e). Building on the complementary properties of the two layers, the shape-fixing capability of the SMP and the reversible actuation of the LCE, we further demonstrated topography locking by quenching in cold water after NIR actuation, and unlocking to the initial flat state by NIR heating followed by ambient cooling (Fig. 7f, g, Supplementary Movies 8, 9).

Compared with surface anchoring-assisted alignment strategies, which require complex and time-consuming fabrication of command layers^33^, our approach of printing smectic LCE filaments onto SMP substrates provides a considerably simpler and straightforward route to programmable and switchable surface topographies. Such dynamically reconfigurable surface textures^58^ are directly relevant to applications in haptics, where tunable tactile feedback is required, and in aerodynamic drag regulation for mobility systems^59,60^.

## Discussion

DIW of LCEs with smectogenic inks provides a promising platform for creating complex director profiles with versatile shape programmability from a single material. This capability stems from the rheological behavior of smectic inks, in which the director orientation can be modulated independently by either temperature or shear rate, enabling reversible switching between perpendicular and parallel alignments. Combined rheological, X-ray scattering, and NEMD studies reveal that this alignment inversion originates from the preservation or collapse of smectic layering near the critical shear rate or phase-transition temperature. Smectic DIW enables complex director profiles even along simple rectilinear paths or within lattice structures, while preserving the geometric fidelity and design freedom inherent to additive manufacturing. We demonstrated that a wide variety of 2D and 3D structures with programmable deformations can be fabricated using this strategy. Beyond LCEs, we anticipate that shear- and temperature-induced orientation switching can be generalized to other layered fluid systems, such as lamellar block copolymer melts^61^. We envision that this smectic DIW platform will serve as a powerful tool for fabricating soft actuators, switchable surface textures for haptic devices and aerodynamic drag control, and minimally invasive biomedical devices, with even greater potential when integrated with inverse design algorithms and multi-material printing^9,11,33,35,62^.

## Methods

### Preparation of the 3D printable smectogenic ink

For the synthesis of smectogenic ink, 4-(6-(acryloyloxy)hexyloxy)phenyl 4-(6-(acryloyloxy)hexyloxy)benzoate (C6BAPE, Chemfish Tokyo Co., Ltd) was used as the diacrylate-functionalised smectogen, n-butylamine (99.5%, Sigma-Aldrich) as the chain extender, and Irgacure 651 (BASF Corporation) as the photoinitiator. All chemicals were used without further purification. C6BAPE and n-butylamine were mixed at a 1.1:1 molar ratio and thoroughly vortexed with heating in the presence of 3.0 wt.% Irgacure 651. The mixture was then transferred to a stainless-steel barrel and oligomerised in a convection oven at 65 °C for 24 h to induce step-growth polymerization via an aza-Michael addition reaction between diacrylate and diamine. The resulting oligomer was used as 3D printing ink.

### 3D printing procedure

3D printing was carried out on a printer (Dr. INVIVO 4D, Rokit Healthcare) equipped with a pneumatic hot-melt dispenser and a stainless-steel nozzle with a diameter of 0.4 mm. Printing height was set to ~0.2 mm above the substrate, and the dispenser pressure was maintained at 500 kPa. Printing paths were generated and converted into G-codes using commercial software (NewCreator K, Rokit Healthcare) and then modified to control the printing speed, x–y motion of the print bed, and z-axis movement of the printhead. To ensure high structural fidelity, the paths were sliced with appropriate infill densities, calculated as the ratio of nozzle diameter (*d*) to filament width (*w*). Single-layer structures were printed onto glass slides and subsequently crosslinked in a custom UV chamber equipped with a 400 W metal-halide lamp (CLEO HPA 400/30 SD L, iSOLde, λ = 315–400 nm) for 10 min. The distance between the lamp and the samples was set to approximately 45 cm, at which the light intensity was measured to be 85 mW cm^–2^. Multi-layered structures were fabricated by layer-by-layer printing, with each layer exposed to weak UV light (OmniCure LX405S, λ = 365 nm, 1.5 mW c^−2^) for 30 s of partial curing before stacking the next layer. The nozzle height was incrementally increased by 0.2 mm for each successive layer. After all layers were printed, the structures were fully cured in a UV chamber for 10 min.

### Preparation of the SMP layer

SMP layers were prepared following the procedure reported by Hwang et al^58^. For the synthesis of the SMP layer, *tert*-butyl acrylate (*t*BAc, 98.0%, Sigma-Aldrich) and *n*-butyl acrylate (*n*BAc, >99.0%, Tokyo Chemical Industry) were used as monomers, while trimethylolpropane ethoxylate triacrylate (*M*_n_ ≈ 912, Sigma-Aldrich) and Irgacure 651 were used as the crosslinker and photoinitiator, respectively. Before use, *t*BAc and *n*BAc were passed through basic alumina columns to remove the inhibitor, 4-methoxyphenol. The crosslinker was used as received. *t*BAc, *n*BAc, crosslinker, and photoinitiator were mixed at a weight ratio of 83.3:14.7:1.0:1.0 and thoroughly vortexed at ambient temperature. The precursor mixture was then injected into a glass cell with a 140 μm gap and filled by capillary action. The cell was subsequently transferred to a UV chamber and polymerized under UV irradiation for 10 min. After polymerization, the top glass slide was removed, and the film attached to the bottom glass slide was used to fabricate bilayered 3D-printed structures (Supplementary Fig. 28).

### Characterization

^1^H NMR spectra were recorded at 600 MHz using an Agilent ProPulse spectrometer with CDCl_3_ as the solvent. SEC analysis was performed on an Agilent 1260 Infinity II system with a G7110B isocratic pump and a G7114A refractive index detector. Tetrahydrofuran (THF) was used as the eluent at 40 °C with a flow rate of 0.5 mL min^−1^. Polystyrene standards were used to construct a calibration curve. Differential scanning calorimetry (DSC) was performed on TA Instruments Discovery DSC 25 between −50 °C and 150 °C at a rate of 10 °C min^−1^ under a nitrogen flow. Rheological measurements of the LC oligomer were carried out using a TA Instruments HR-20 rotational rheometre equipped with a Peltier plate, employing a parallel-plate geometry with a 25 mm diameter and a 1 mm gap. Samples were loaded onto the Peltier plate, and viscosities were measured under rotational shear flow between parallel plates with a shear rate from 0.001 to 200 s^−1^ at various temperatures. To observe the dimensions and thermal deformations of the printed structures, a Nikon Eclipse LV100N POL microscope with a Linkam LTS420 heating stage was used for optical microscopy. Cyclic actuation, blocking stress, and viscoelastic properties were measured using a dynamic mechanical analyzer (DMA Q850, TA Instruments) equipped with a tension film clamp. For cyclic actuation measurements of printed films, rectangular samples [12 mm (L) × 3 mm (W)] were subjected to repeated heating–cooling cycles between 10 and 100 °C at a rate of 3 °C min^−1^ under a constant stress of 1 kPa, and the resulting strain changes were monitored. The blocking stresses (or actuation stresses) of elongational and contractile LCEs were measured under 0.1% isostrain during heating–cooling cycles between 0 and 120 °C at 3 °C min^−1^. An 8 × 8 × 8 mm^3^ cube printed at *T*_nozzle_ = 35 °C and v_nozzle_ = 10 mm s^−1^ was tested in compression mode to measure the elongational actuation stress, whereas a 15 × 5 × 0.2 mm^3^ film printed at *T*_nozzle_ = 45 °C and v_nozzle_ = 20 mm s^−1^ was tested in tensile mode to measure the contractile actuation stress. The actuation stability of the LCE film was evaluated over 100 heating–cooling cycles between 10 and 100 °C at a rate of 5 °C min^−1^ under a constant applied stress of 1 kPa. Because approximately 25 cycles could be performed with a fully filled gas cooling accessory (GCA), the GCA was refilled with liquid nitrogen before the 26th, 53rd, and 78th cycles to enable testing over 100 cycles. Viscoelastic properties of LCE and SMP were measured using the temperature-ramp mode. Polydomain LCE and SMP films [15 mm (L) × 5 mm (W) × 0.2 mm (T)] were heated from −50 to 180 °C at a rate of 3 °C min^−1^ under a constant frequency of 1 Hz and an amplitude of 15 μm. Load-bearing performance of the deformed structures and temperature-dependent variations in pore area of the lattice structures were recorded using a smartphone camera and quantitatively analysed with ImageJ software (version 1.54 g).

### WAXS experiment

Wide-angle X-ray scattering (WAXS) experiments were performed at the 9 A U-SAXS beamline of the Pohang Accelerator Laboratory (PAL), Republic of Korea, using a 2D charge-coupled device area detector (MX170-HS, Rayonix, LLC). The X-ray wavelength (λ) was set to 1.121 Å (E = 11.06 keV), with a sample-to-detector distance of 210.7 mm. From the two-dimensional patterns, 1D intensity profiles were extracted as a function of the scattering vector *q* (*q* = (4π/λ)sin(θ/2), where θ is the scattering angle). The ${P}_{2}(\cos \phi )$ values were calculated from azimuthal intensity distributions according to the following equation^63^:

3 $${P}_{2}(\cos \phi )\,=1-{N}^{-1}\frac{3}{2}{\int }_{\!\!\!0}^{\frac{\pi }{2}}I\left(\phi \right)\left[{\sin }^{2}\phi+\left(\sin \phi {\cos }^{2}\phi \right){\mathrm{ln}}\frac{1+\sin \phi }{\cos \phi }\right]d\phi,$$

where $N={\int }_{0}^{\frac{\pi }{2}}I\left(\phi \right)d\phi,\phi$ is the azimuthal angle, and *I*(ϕ) is the azimuthal angle-dependent intensity at a constant scattering vector *q*.

### MD simulations

The mesogens were modeled as eight-bead, purely repulsive LJ chains with bonds maintained using finitely extensible nonlinear elastic (FENE) potentials following the model by Milchev^64^. To ensure mesogen rigidity and a rod-like conformation, a bending potential was imposed on two neighboring bonds, with an energy of 96 ${k}_{B}T$ in reduced LJ units. The simulation box consisted of 12,696 mesogens (101,568 LJ beads) at a number density of ρ=0.8125 ${{{\rm{\sigma }}}}^{-3}$.

NEMD simulations were performed at a fixed temperature of *T* = 0.5 using a dissipative particle dynamics (DPD) thermostat implemented in the Large-scale Atomic/Molecular Massively Parallel Simulator (LAMMPS) package^65^, allowing the study of simple shear deformation^66,67^. In these simulations, the protocol by Milchev was modified by employing a triclinic box, which allowed the system to tilt and thereby facilitated the formation of Smectic-A layers. This was achieved by applying a barostat on ${P}_{{xy}}$, ${P}_{{xz}}$, and ${P}_{{yz}}$, ensuring that these pressure components were maintained at zero. In doing so, the smectic layers were able to orient correctly by minimizing the cross-components of the pressure tensor. Structural characterization of the NEMD trajectories, including layer order and orientational order parameters, was conducted once the system reached a steady state. Visualization of the system was performed using OVITO software^68^.

### FEA of LCE thermal deformations

Finite element analysis was carried out using ABAQUS 2023 software to predict the thermal deformation of 3D-printed LCEs. The simulated geometries replicated the actual printed dimensions and were modeled as linear thermoelastic materials with anisotropic thermal expansion and a Poisson’s ratio of 0.49. All models were discretised using eight-node brick elements (C3D8) with temperature and displacement degrees of freedom and a hybrid formulation for incompressibility. Temperature-dependent thermal expansion coefficients parallel to the print axis (α_xx_) for elements printed at 3, 5, and 10 mm s⁻^1^ were taken from measurements (Fig. 4a), while perpendicular coefficients (α_yy_ and α_zz_) were set to −α_xx_/2. (Supplementary Note 4, Supplementary Fig. 27) The starting and ending temperatures were set to 20 °C and 100 °C, respectively. Fixed boundary conditions were applied to elements in the small central circular region of the mesh to simulate the free-standing state of the LCEs. All simulations used a coupled temperature–displacement step with nonlinear geometry (NLGEOM = ON) to capture large deflections and hyperelastic behaviors. Vertical nodal displacements are reported in millimeters (Fig. 6).

## Supplementary information

Supplementary Information

Description of Additional Supplementary Files

Supplementary Movie 1

Supplementary Movie 2

Supplementary Movie 3

Supplementary Movie 4

Supplementary Movie 5

Supplementary Movie 6

Supplementary Movie 7

Supplementary Movie 8

Supplementary Movie 9

Transparent Peer Review file

## Source data

Source Data

## Data Availability

The data supporting the findings of this study are included in the Supplementary Information or are available from the corresponding author upon request. Source data are provided with this paper.
