# Supplementary material for: Alignment switching in 3D-printed smectic liquid crystal elastomers
Source: Nat Commun. 2026 Jul 10;17:8530. doi: 10.1038/s41467-026-75368-z (PMC13483228; doi:10.1038/s41467-026-75368-z)
Supplement: Supplementary file 1 [file 41467_2026_75368_MOESM12_ESM.pdf]

nature portfolio

Peer Review File

Alignment Switching in 3D-Printed Smectic Liquid Crystal Elastomers

Corresponding Author: Professor Suk-kyun Ahn

This file contains all reviewer reports in order by version, followed by all author rebuttals in order by version.

Version 0:

Reviewer comments:

Reviewer #1

(Remarks to the Author)
This manuscript reports a direct ink writing (DIW) strategy for fabricating smectic liquid crystal elastomers (LCEs) that exhibit reversible director switching between perpendicular and parallel orientations within a single printing path, achieved through the combined control of printing temperature and speed. The authors successfully demonstrate how this shear–temperature-dependent alignment inversion enables both contractile and elongational actuation modes in a single material system. Although the manuscript is carefully executed and the data are of good quality, it does not provide a sufficiently original conceptual or methodological advance. Several key issues require clarification and additional quantitative evidence.

1. The key ideas—smectic layer collapse under shear, resulting orientation inversion, and the corresponding switch between elongational and contractile actuation—have already been reported in the authors' own recent publication (Lee et al., Sci. Adv., 2025. 11, 3, eadt7613. DOI: 10.1126/sciadv.adt7613), which established the same physical mechanism using a melt-spinning process. The central mechanism of shear and temperature induced orientation flipping in smectic mesophases has also been extensively discussed in previous literature. The present manuscript largely transfers that known concept to a DIW printing platform without introducing a genuinely new physical insight, processing principle, or functional demonstration.

2. The DIW process employed here differs only superficially from conventional extrusion-based printing of nematic LCEs. The work lacks a clear demonstration of what DIW specifically enables that cannot be achieved through melt-spinning or other established alignment techniques. The presented 3D structures are relatively simple and mainly serve as qualitative proofs of concept. There is no demonstration of functional devices, complex motion sequences, or practical integration that would highlight the transformative potential of this technique.

3. The manuscript should also provide more quantitative comparison with the prior fiber-based approach in terms of shear-rate range, order parameters, actuation strain, or spatial resolution of director programming. Without such differentiation, it is difficult to identify what new capability this DIW method offers.

4. The manuscript repeatedly uses "printing speed" and "temperature" as control variables but does not provide a quantitative mapping to actual shear rates inside or at the nozzle exit. To ensure reproducibility and cross-platform generality, the authors should estimate or simulate the shear rate distribution based on nozzle geometry, pressure, and extrusion speed.

5. The "mixed" alignment regime near the phase transition is attributed to orientation gradients across the filament thickness, but this remains speculative. Please provide spatially resolved characterization, such as microbeam WAXS mapping, polarized optical/ Raman microscopy, or cross-sectional analysis to directly confirm the through-thickness or lateral director variation.
6. The presented actuation tests lack long-term cycling data. For assessing functional robustness, please report strain retention or hysteresis after multiple (e.g., >100) heating–cooling cycles.

Reviewer #2

(Remarks to the Author)

This study reports a DIW strategy that enables shear- and temperature-dependent orientation switching in smectic LCE inks. By tuning the print temperature and speed, the mesogen orientation can be switched between perpendicular and parallel alignment within a single filament, which allows both contractile and elongational actuation modes to coexist within a single material system. The approach successfully demonstrates the printing of LCE structures capable of complex shape transformations.
I have the following questions and comments:
1. It remains unclear how the non-equilibrium molecular dynamics (NEMD) simulation results contribute to understanding the DIW mechanisms. The simulations operate at vastly different spatial and temporal scales from the experiments, and there is no direct comparison between the simulated outcomes and the experimental measurements. The authors are suggested to clarify the intended purpose of the simulations, whether they are primarily conceptual or quantitatively predictive, and to discuss the limitations associated with the significant disparity in scales. Without clear validation or correlation, it is difficult to assess how the simulated insights can directly guide experimental understanding or process optimization.
2. The proposed orientation switching relies on adjusting either the print speed or the print temperature. While varying the print speed is practical, temperature control may limit the scalability or continuity of this method. Frequent temperature adjustments, particularly cooling, during printing could be time-consuming and may affect the homogeneity and interfacial bonding of printed filaments. In Figure 5, the printed structures appear to exhibit domain boundaries among regions printed at different speeds or temperatures. The authors should discuss how these transition regions affect the mechanical integrity and actuation uniformity of the printed samples. Do these boundary regions maintain uniform thickness? Furthermore, what is the smallest achievable size of independently controlled mesogen domains?
3. In the finite element simulations, it is stated that the transverse thermal expansion coefficients are set to $-\alpha xx/2$. What is the rationale behind this choice? Does this relationship ensure incompressibility of the material, where the product of stretch ratios along the x, y, and z directions equals 1? In addition, how does the model ensure that each finite element continues to contract or elongate along its local filament direction during large deformation, given that the filament direction itself may rotate as deformation progresses? More explanation of these modeling assumptions and their physical implications would strengthen the analysis.

Reviewer #3

(Remarks to the Author)
The manuscript reports a DIW strategy for smectic liquid crystal elastomers that enables switching between perpendicular and parallel molecular orientation within a single printed filament. This is an interesting contribution to the field of programmable soft materials. The idea of exploiting shear- and temperature-dependent alignment of smectic layers is novel and potentially impactful. However, the manuscript in its current form requires major revision before it can be considered for publication.
1.The manuscript would benefit from a clearer definition of the limitations in current DIW-based LCE alignment methods. In particular, outlining how existing single-mode strategies constrain programmability—and briefly comparing with previously reported multi-mode alignment approaches—would help better establish the research gap and highlight the contribution of this work.
2.It would strengthen the manuscript to provide a more systematic analysis of the printing processing window. At present, only a speed–temperature map is shown, but key factors such as nozzle diameter, ink viscosity range, and shear-rate quantification are not discussed. In addition, the manuscript does not clarify whether the same alignment behavior holds at different printing heights or under varying filament overlaps, both of which may affect director orientation and the resulting actuation strain. A more comprehensive evaluation of printing robustness would improve the clarity and reproducibility of the proposed method.
3.While the manuscript claims “significantly expanded design freedom” and “diverse programmed shape changes,” the demonstrations focus mainly on relatively simple 2D/3D structures. Including more complex or multi-step morphing examples—such as sequential folding, hierarchical deformation, or spatially coupled transformations—would better substantiate these statements and more convincingly showcase the advantages of the proposed approach.
4.The manuscript would benefit from additional characterization of actuator performance. In particular, data on cyclic stability (fatigue behavior), response time, and mechanical output (e.g., generated force or stress) are not provided. These metrics are essential for evaluating the robustness and practical applicability of the printed LCE actuators, and including them would substantially strengthen the work.

5.The manuscript would benefit from clearer reporting of the UV-curing conditions (intensity, exposure time, distance), as these parameters can influence director relaxation and final alignment.
6.The origin of the “mixed alignment” state is mentioned but not fully clarified. More discussion or supportive data would help readers understand the transition regime between perpendicular and parallel alignment.
7.Although NEMD simulations qualitatively reproduce the alignment transition, a more explicit quantitative comparison between simulated and experimental shear-rate thresholds would strengthen the mechanistic interpretation.

Version 1:

Reviewer comments:

Reviewer #1

(Remarks to the Author)
The authors have sucessfully addressed my concerns and this manuscript can be published.

Reviewer #2

(Remarks to the Author)
The authors have adequately addressed my comments. I recommend acceptance of the manuscript.

Reviewer #3

(Remarks to the Author)
The authors have adequately addressed the concerns raised in the previous review. The revised manuscript has been significantly improved through the addition of a more comprehensive discussion of the research gap, expanded characterization of the processing window, additional demonstrations of programmable shape morphing, and further evaluation of actuator performance. The revisions strengthen both the scientific rigor and the practical relevance of the work. I appreciate the authors’ thorough responses and the substantial efforts made to improve the manuscript. In my opinion, the manuscript is now suitable for publication in its present form.
Recommendation: Accept.

Open Access This Peer Review File is licensed under a Creative Commons Attribution 4.0 International License, which permits use, sharing, adaptation, distribution and reproduction in any medium or format, as long as you give appropriate credit to the original author(s) and the source, provide a link to the Creative Commons license, and indicate if changes were made.
In cases where reviewers are anonymous, credit should be given to 'Anonymous Referee' and the source.
The images or other third party material in this Peer Review File are included in the article’s Creative Commons license, unless indicated otherwise in a credit line to the material. If material is not included in the article’s Creative Commons license and your intended use is not permitted by statutory regulation or exceeds the permitted use, you will need to obtain permission directly from the copyright holder.
To view a copy of this license, visit https://creativecommons.org/licenses/by/4.0/

REVIEWER COMMENTS

Reviewer #1 (Remarks to the Author):

This manuscript reports a direct ink writing (DIW) strategy for fabricating smectic liquid crystal elastomers (LCEs) that exhibit reversible director switching between perpendicular and parallel orientations within a single printing path, achieved through the combined control of printing temperature and speed. The authors successfully demonstrate how this shear–temperature-dependent alignment inversion enables both contractile and elongational actuation modes in a single material system. Although the manuscript is carefully executed and the data are of good quality, it does not provide a sufficiently original conceptual or methodological advance. Several key issues require clarification and additional quantitative evidence.

Response: We are grateful to the reviewer for carefully reading the manuscript. We also appreciate the reviewer's essential comments on our work which improve the quality of our manuscript. We have tried our best to revise this article according to the comments of the reviewer. A point-by-point response is given in the following.

1. The key ideas—smectic layer collapse under shear, resulting orientation inversion, and the corresponding switch between elongational and contractile actuation—have already been reported in the authors' own recent publication (Lee et al., Sci. Adv., 2025. 11, 3, eadt7613. DOI: 10.1126/sciadv.adt7613), which established the same physical mechanism using a melt-spinning process. The central mechanism of shear and temperature induced orientation flipping in smectic mesophases has also been extensively discussed in previous literature. The present manuscript largely transfers that known concept to a DIW printing platform without introducing a genuinely new physical insight, processing principle, or functional demonstration.

Response:

We thank the reviewer for this thoughtful comment. We agree that the shear rate- and temperature-dependent alignment behavior of smectic LCs constitutes the underlying physical framework shared with our previous work (Lee et al., *Sci. Adv.* 2025, *11*, eadt7613). However, we would like to clarify that the transition from melt-spinning to DIW is not merely a change of processing platform, but rather enables a new distinct capability that is structurally inaccessible in melt-spinning. Specifically, DIW allows **spatially programmable director inversion within a single continuous print path** that has not been achieved in any prior work on LCEs.

In our previous melt-spinning work, the accessible shear-rate range during fiber extrusion was inherently limited by the process geometry. Moreover, temperature control, though effective for global alignment, is relatively slow and therefore cannot achieve spatial modulation of the director within a single extruded filament. As a result, each fiber possessed only a single, globally uniform director orientation (either perpendicular or parallel to the filament axis). **The critical advance of the present work lies in exploiting the programmable nozzle translation speed in DIW, which allows the applied shear rate to be tuned nearly instantaneously over a broad range, enabling locally distinct director orientations to be encoded at user-defined positions along a single printed filament**. This spatial programmability, which enables switching between elongational and contractile actuation domains within one continuous path, represents a new processing principle and a new functional degree of freedom that goes beyond our previous work on melt-spun fibers of smectic LCEs.

Regarding the reviewer's note that orientation inversion in smectic mesophases has been discussed in previous work: while the general phenomenon is established, its practical implementation in an additive manufacturing context for spatially programmable shape-morphing has not been realized. The present work demonstrates, for the first time, that this mechanism can be harnessed at the level of discrete segments along a printed filament, enabling both contractile and elongational actuation modes within a single-material, single-process fabrication route.

To further substantiate these distinctions and to address the reviewer's concern, we have added the following experimental results in the revised manuscript. (1) As a functional demonstration, we fabricated reversibly switchable surface topography printed on shape memory polymer (SMP) substrates enabled by nozzle-speed programming; these results are discussed in detail in **Q2**. (2) A quantitative comparison highlighting the advantages of DIW of smectic LCEs over our previous melt-spinning approach is provided in **Q3.** (3) A systematic study of the effects of ink viscosity, nozzle diameter, and printing height on smectic LC alignment is discussed in detail in **Q4**.

2. The DIW process employed here differs only superficially from conventional extrusion-based printing of nematic LCEs. The work lacks a clear demonstration of what DIW specifically enables that cannot be achieved through melt-spinning or other established alignment techniques. The presented 3D structures are relatively simple and mainly serve as qualitative proofs of concept. There is no demonstration of functional devices, complex motion sequences, or practical integration that would highlight the transformative potential of this technique.

<u>Response</u>:

We thank the reviewer for this constructive comment. We would like to address each of the raised points in turn.

First, regarding the distinction from conventional DIW with nematic LCEs: the critical difference lies in the shear-rate-dependent director inversion unique to smectic mesophases. In nematic LCEs, the director aligns uniformly along the shear direction regardless of printing speed, so spatial director inversion within a single filament is not achievable by nozzle speed control alone. By contrast, in smectic LCEs, the director orientation can be switched between perpendicular and parallel to the filament axis simply by adjusting the nozzle translation speed (v_{nozzle}) at a fixed temperature. This enables spatial director programming within a single continuous print path, a capability that is fundamentally inaccessible in DIW with nematic LCEs.

To demonstrate this capability, we printed smectic LCE filaments at different v_{nozzle} values at a constant temperature of 40 °C onto a SMP substrate, consisting of poly(*tert*-butyl acrylate-*co*-*n*-butyl acrylate) (**Fig. R1a**). The resulting bilayers underwent shape transformation into either convex or concave geometries with tunable curvature ($\kappa \approx 0.1$–0.3 mm^{-1}) (**Fig. R1b**). Moreover, by varying v_{nozzle} across different sections within a single filament, periodic undulations with tunable wavelength could be generated (**Fig. R1c**). Notably, both spatial director programming and the resulting complex topographic deformations were achieved using simple rectilinear print paths, which are not attainable with DIW with nematic LCEs.

We have included the following sentences in the revised manuscript on **Page 13, lines 358–369**.

"To further highlight the programming capability of shear rate-induced orientation control of in smectic DIW, we printed smectic LCE filaments onto a flexible shape memory polymer (SMP) substrate, consisting of poly(tert-butyl acrylate-co-n-butyl acrylate), at different v_{nozzle} values at a constant temperature of 40 °C (Fig. 6b–d and Supplementary Fig. 28). The resulting bilayers underwent thermal deformation into either convex or concave geometries with tunable curvature ($\kappa \approx$ 0.1–0.3 mm^{-1}) to accommodate the strain mismatch between the passive SMP layer and the active LCE layer, whose elongation or contraction was programmed by varying v_{nozzle}. (Fig. 6c). Moreover, by alternating v_{nozzle} between low and high values along a single filament, periodic undulations with tunable wavelength could be generated (Fig. 6d). Using a 0.4 mm-diameter nozzle, local segments as short as 1 mm with distinct director orientations could be programmed within a single printed filament (Supplementary Fig. 29). This spatial resolution of director programming is expected to depend on nozzle geometry and ink properties."

(**Fig. R1** has been included to the **Fig. 6b–d** on the revised manuscript.)

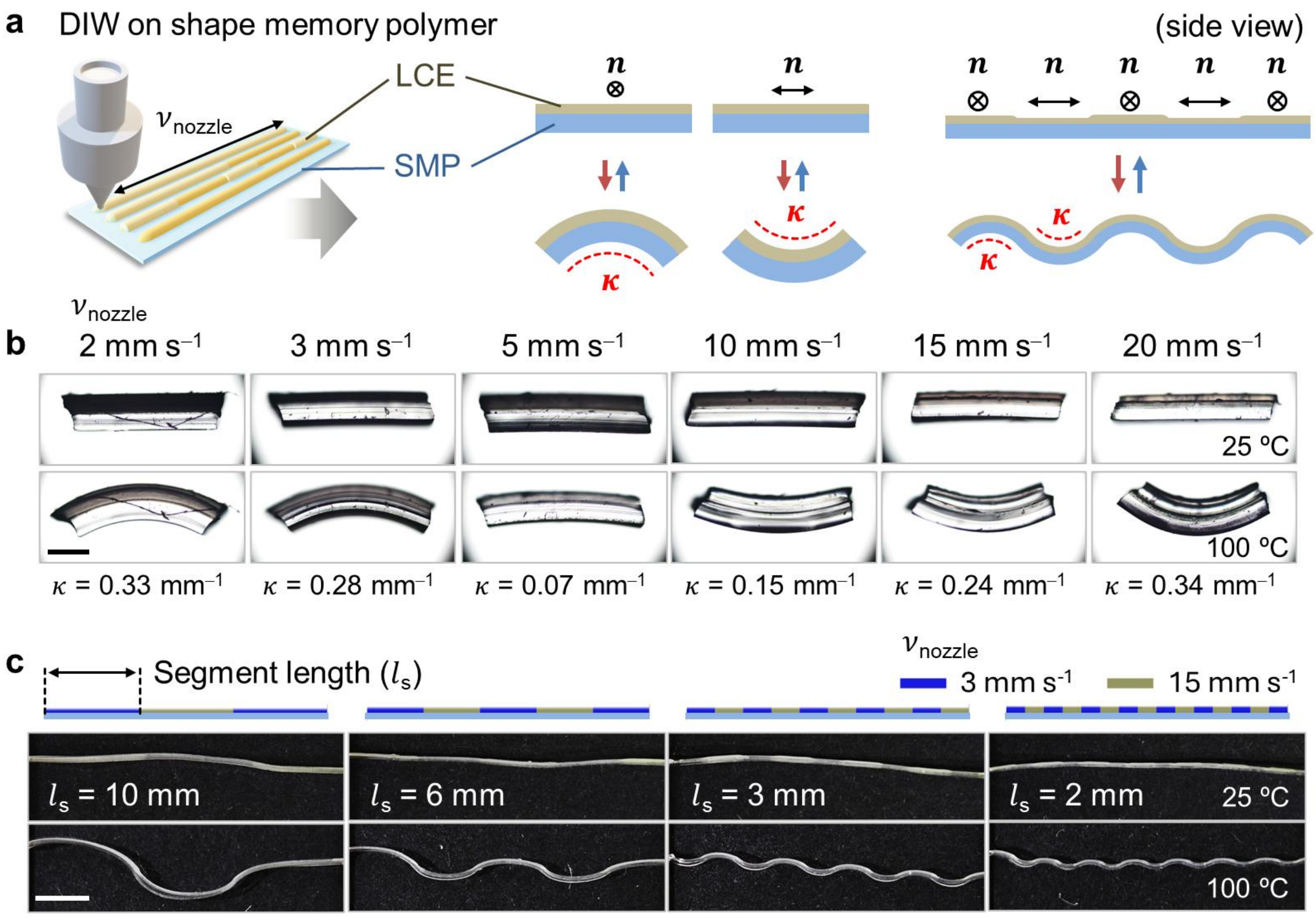

Figure R1. (a) Schematic illustration of shear rate controlled direct-ink-writing (DIW) of smectic liquid crystal elastomers (LCEs) onto a shape memory polymer (SMP) substrate, poly(*tert*-butyl acrylate-*co*-*n*-butyl acrylate). Double arrows indicate the director ($\vec{n}$). (b) Optical microscope images showing side views of bilayer filament segments at 25 °C (unactuated) and 100 °C (actuated), printed at different nozzle translation speeds (v_{nozzle}). The curvature, κ (mm^{-1}), is defined as $1/R$, where R is the radius of curvature. Scale bar = 500 µm. (c) Optical images showing side views of bilayer filaments at 25 °C (unactuated) and 100 °C (actuated), printed with alternating v_{nozzle} of different periodicities. Scale bar = 5 mm.

Second, to address the reviewer's concern regarding the absence of functional device demonstrations, we additionally fabricated **reversibly switchable surface topography actuators** (**Fig. R2**). Smectic LCE filaments were printed onto SMP substrates and coated with a black pigment to enable photothermal actuation (**Fig. R2a**). As the SMP layer, we used poly(*tert*-butyl acrylate-*co*-*n*-butyl acrylate), which exhibits an abrupt modulus change around its glass transition temperature ($T_g \approx$ 41 °C), thereby enabling shape locking and unlocking upon rapid cooling and ambient cooling, respectively (**Fig. R2b–c**). Rectilinear print paths were encoded with different v_{nozzle} values section by section as described in the left panels of **Fig. R2d**. Notably, each printed structure exhibited complex programmable surface topographies upon NIR irradiation, including (1) wavy stripes, (2) golf ball dimples, and (3) a human face, as shown in the middle and right panels of **Fig. R2d**, and could return to its initial shape by ambient cooling. **Fig. R2e** demonstrates that this actuation is fully reversible, switching between the initial flat and topographic states upon NIR irradiation and ambient cooling. Building on this, we combined the advantages of both the SMP and LCE layers: the ability of the SMP to fix a temporarily deformed shape and the reversible actuation capability of the LCE. This allows locking of the NIR-actuated surface topography by quenching in cold water prior to shape recovery (i.e., shape fixing), and unlocking to the initial shape by NIR heating followed by ambient cooling (**Fig. R2f–g and Supplementary Movie 8–9**).

We note that our approach (i.e., smectic LCE printing on SMP substrates) provides a straightforward route to realizing programmable surface topographies, and is considerably simpler than surface anchoring-assisted alignment strategies, which require inverse design and time-consuming fabrication of command layers. [Aharoni, H., *et al. Proc. Natl. Acad. Sci. U.S.A.* **115**, 7206–7211 (2018)] Our dynamically switchable surface textures are directly relevant to applications in haptics, where tunable tactile feedback is required, and in aerodynamic drag regulation for mobility systems. [Li, T. *et al. Matter* **8**, 102345 (2025)]

Accordingly, the functional demonstration of smectic DIW has been discussed in a new section, "Switchable surface topography actuators," in the revised manuscript on **Page 13, lines 372–399**, as follows:

Switchable surface topography actuators

"Finally, we fabricated reversibly switchable surface topography actuators by integrating smectic LCE filaments with a SMP substrate (Fig. 7). The LCE filaments were printed onto SMP substrates and coated with a black pigment to enable photothermal actuation (Fig. 7a). The SMP layer exhibits an abrupt modulus change from ≈470 MPa at 25 °C to ≈0.3 MPa at 100 °C around its glass transition temperature ($T_g \approx$ 41 °C). In contrast, the LCE layer shows a more gradual modulus transition over the same temperature range, decreasing from 3 MPa at 25 °C to ≈0.7 MPa at 100 °C (Fig. 7c). In the bilayer configuration, the LCE governs the mechanical response above T_i, whereas the SMP dominates at room temperature ($T < T_g$). As a result, the bilayer is sufficiently compliant to undergo large actuation above T_i, yet becomes rigid upon cooling to room temperature. We exploit this temperature-dependent mechanical contrast between the two layers to achieve shape locking upon rapid quenching and shape unlocking upon ambient cooling (Fig. 7b). [57]

Rectilinear print paths were encoded with spatially varying v_{nozzle} values to program distinct director profiles section by section (left panels, Fig. 7d). Upon near-infrared (NIR) irradiation, the

printed structures exhibited preprogrammed surface topographies, including wavy stripes, golf ball dimples, and a human face, and returned to their initial flat state upon ambient cooling (middle and right panels, Fig. 7d; Supplementary Movie 7). This actuation is fully reversible across multiple cycles (Fig. 7e). Building on the complementary properties of the two layers, the shape-fixing capability of the SMP and the reversible actuation of the LCE, we further demonstrated topography locking by quenching in cold water after NIR actuation, and unlocking to the initial flat state by NIR heating followed by ambient cooling (Figs. 7f–g and Supplementary Movie 8–9).

Compared with surface anchoring-assisted alignment strategies, which require complex and time-consuming fabrication of command layers [33], our approach of printing smectic LCE filaments onto SMP substrates provides a considerably simpler and straightforward route to programmable and switchable surface topographies. Such dynamically reconfigurable surface textures[58] are directly relevant to applications in haptics, where tunable tactile feedback is required, and in aerodynamic drag regulation for mobility systems.[59,60]"

(**Fig. R2** has been included to the **Fig. 7** on the revised manuscript.)

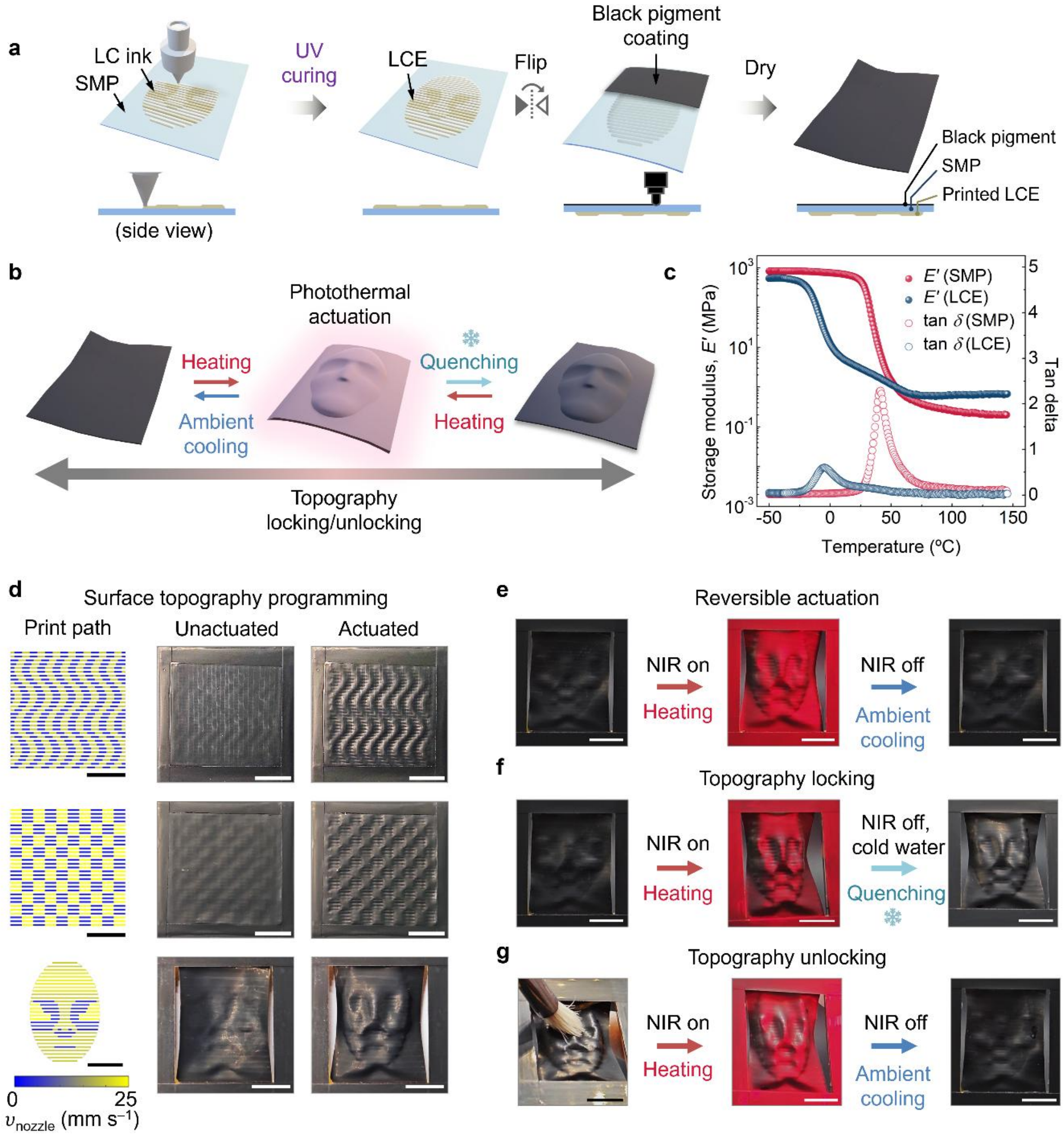

Figure R2. Reversibly switchable surface-topography actuator fabricated via shear rate controlled DIW of smectic LCEs onto SMP substrates. (a) Schematic illustration of fabrication process. Smectic LC filaments were printed onto SMP substrates at varying print speeds at 40 °C and subsequently UV-cured for 10 min. The opposite side of the printed surface was then coated with a black pigment to enable photothermal actuation and dried for 10 min under ambient conditions. (b) Schematic illustration of reversible surface-topography switching of the actuator, arising from the actuation of the LCE layer and the shape-fixing capability of the SMP layer. (c) Viscoelastic properties of the smectic LCE and SMP measured by DMA. (d) Photographs of programmed surface-topography patterns, including wavy stripes (top), golf ball dimples (middle), and a human face (bottom), generated from rectilinear print paths with spatially encoded print speeds. Scale bars, 1 cm. (e–g) Snapshots of the transformable topography actuator programmed with a human-face pattern, showing reversible actuation (e), topography locking (f), and topography unlocking (g). Scale bars, 1 cm.

3. The manuscript should also provide more quantitative comparison with the prior fiber-based approach in terms of shear-rate range, order parameters, actuation strain, or spatial resolution of director programming. Without such differentiation, it is difficult to identify what new capability this DIW method offers.

Response:

We appreciate the reviewer's comment. Smectic DIW in this work was conducted primarily under the following conditions: nozzle height of 0.15–0.25 mm, nozzle diameter of 0.4 mm, and nozzle translation speed of 2–30 mm s^{-1}. These printing conditions provide access to a shear-rate range of 8–200 s^{-1}, covering the second shear-thinning regime (around 60 s^{-1}) of the smectic ink in steady-shear viscosity measurements shown in **Fig. R3**. Within this processing window, the order parameter of printed filaments can be tuned at a fixed nozzle temperature of 40 °C from 0.17 (perpendicular director orientation) to 0.26 (parallel orientation). This translates into programmable actuation behavior, ranging from 8.4% thermal elongation to 25.7% thermal contraction. By further adjusting the nozzle temperature (35–45 °C) and printing speed (2–30 mm s^{-1}), the actuation response can be graded from +28% elongation to −31% contraction. Compared with our previous fiber-extrusion approach, which produced elongating LCE fibers with an order parameter of 0.20 (30% elongation) at 35 °C and contracting fibers with an order parameter of 0.42 (23% contraction) at 50 °C, **the DIW strategy provides broader and more flexible control of actuation behavior through processing parameters**.

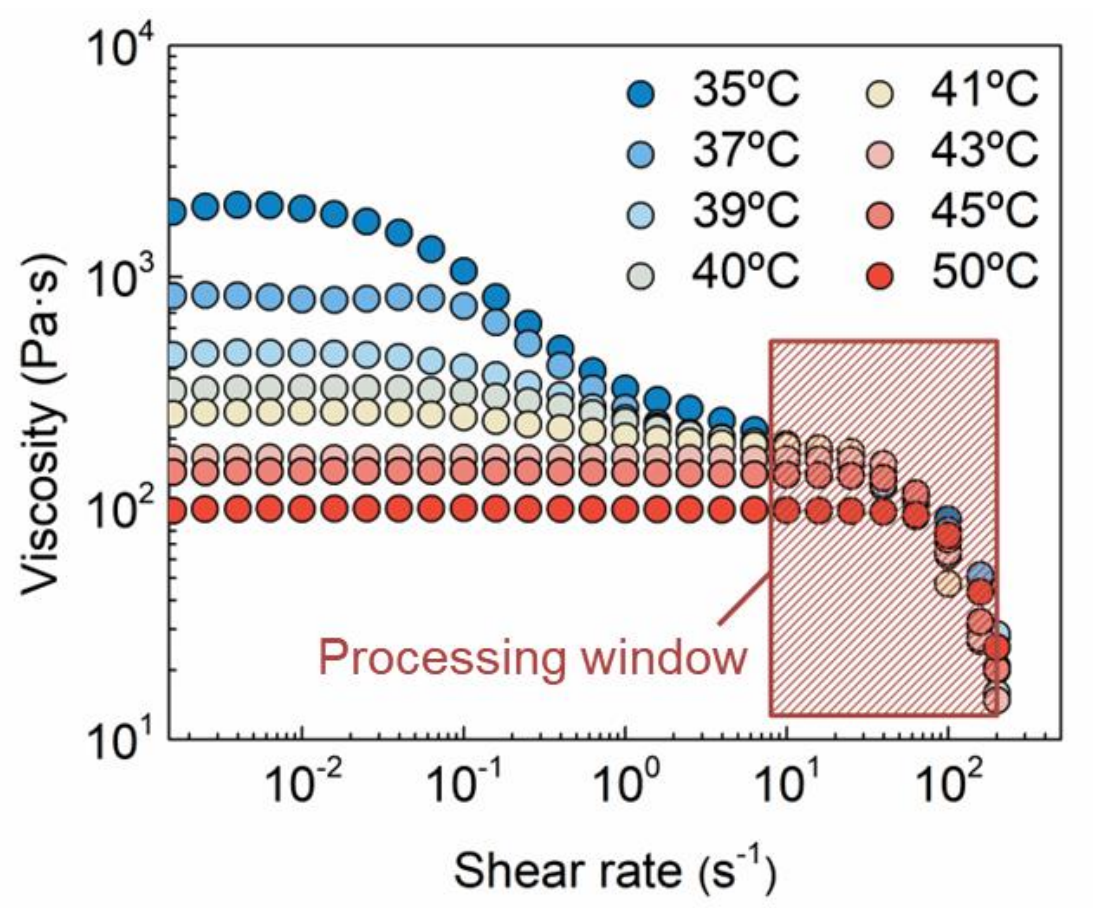

Figure R3. Steady shear viscosity as a function of shear rate at various temperatures for the smectic oligomer.

In the revised manuscript on **Page 10, lines 264–270**, we have added a quantitative comparison with the prior fiber-based approach in terms of order parameter and actuation strain, as follows:

"*By adjusting T_{nozzle} (35–45 °C) and v_{nozzle} (2–20 mm s^{-1}), the actuation response of a single filament can be graded from +28% elongation to −31% contraction, indicating that smectic DIW provides broader and more flexible control over actuation behaviour than conventional nematic DIW and fiber-extrusion approaches. [25,50] For example, modulating the printing parameters along the same concentric print path allows the same printed material to exhibit contrasting deformation modes—saddle-like (elongation) and cone-like (contraction) (Fig. 4d,e).*"

The prior fiber-based approach could produce only LCE filaments with a single, uniform

director alignment, either perpendicular or parallel to the filament axis. **In contrast, the present smectic DIW approach enables the fabrication of spatially patterned LCE filaments containing locally distinct director orientations** (**Fig. R4**). As shown in **Fig. R4**, alternating v_{nozzle} between low and high values within a single print path produces filaments with periodically inverted director orientations, which upon actuation generate correspondingly distinct local deformation modes. Using a 0.4 mm-diameter nozzle, local segments as short as 1 mm with different director orientations could be programmed within a single printed filament. **Fig. R1c** further demonstrates that the spatially programmed director pattern gives rise to correspondingly distinct local actuation shapes. The spatial resolution of director programming is expected to depend on nozzle geometry.

(**Fig. R4** has been included as **Supplementary Fig. 29** in the revised Supplementary Materials.)

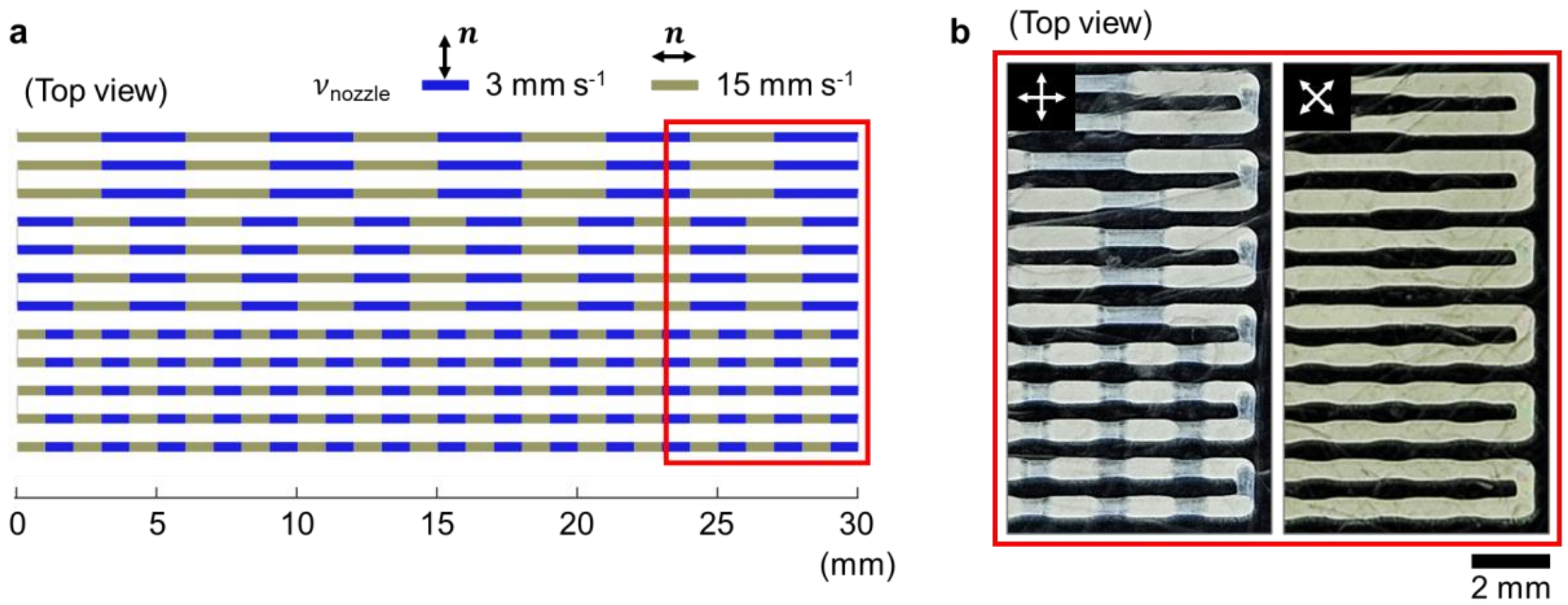

Figure R4. Smectic LCE filaments spatially programmed with alternating director patterns of different section lengths via encoded print speeds (v_{nozzle}). (a) Print path with encoded print speeds. Double arrows indicate the director ($\vec{n}$). (b) Polarized optical image of the printed filament acquired between crossed polarizers aligned (left) parallel to and (right) rotated by 45° with respect to the filament axis. Sections printed at v_{nozzle} = 3 mm s^{-1} and v_{nozzle} = 15 mm s^{-1} exhibit different birefringence intensities. White arrows indicate the orientations of the polarizer and analyzer.

4. The manuscript repeatedly uses "printing speed" and "temperature" as control variables but does not provide a quantitative mapping to actual shear rates inside or at the nozzle exit. To ensure reproducibility and cross-platform generality, the authors should estimate or simulate the shear rate distribution based on nozzle geometry, pressure, and extrusion speed.

Response:

We appreciate the reviewer for the constructive comment. We agree that providing a quantitative mapping of shear rates is essential for reproducibility and cross-platform applicability. In response, we have performed a comprehensive series of analyses, including **(1) shear rate conversion, (2) volumetric flow rate measurements, (3) shear-rate-governed orientation inversion, (4) COMSOL CFD simulations of in-nozzle shear rate distribution, and (5) molecular weight-dependence of smectic ink on shear alignment and actuation**, to establish the relationship between processing conditions and shear-rate-governed director alignment in smectic LCEs. These are described in detail below.

(1) Shear rate conversion and heat map re-plotting (Fig. R5)

We have re-plotted the heat map of maximum actuation strain (ε_a) with shear rate ($\dot{\gamma}$) on the x-axis to improve reproducibility and cross-platform generality (**Fig. R5**). The shear rate was estimated using a simple Couette flow approximation:

$$\dot{\gamma} = \frac{velocity\ difference\ between\ plates}{distance\ seperating\ plates} = \frac{v_{\mathrm{nozzle}}}{H}$$

where v_{nozzle} is the nozzle translation speed and H is the gap distance between the nozzle tip and the substrate (set to $H = 0.2$ mm in this study). The pneumatic pressure applied to the print barrel was fixed at $P = 500$ kPa throughout.

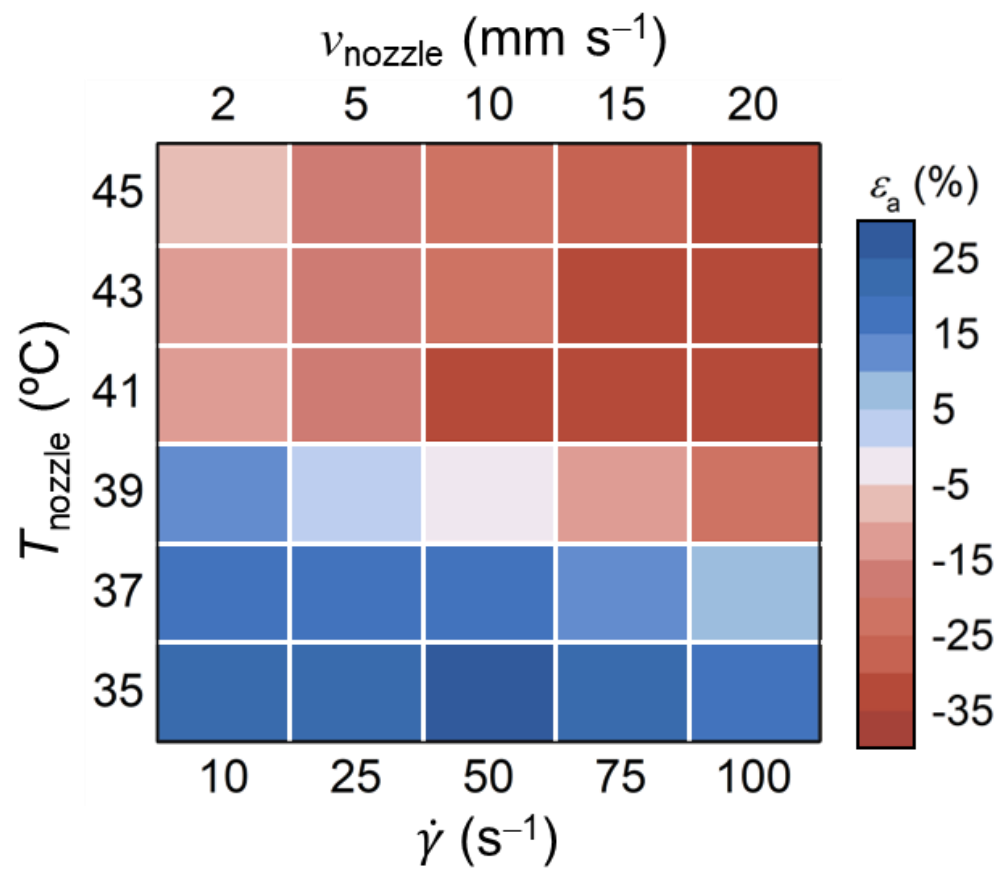

Figure R5. Heat map of actuation strain (ε_a) measured at 100 °C for filaments printed at varying temperatures (T_{nozzle}, left axis) and nozzle translation speeds (v_{nozzle}, top axis). The corresponding shear rates ($\dot{\gamma}$, bottom axis) were calculated as $\dot{\gamma} = v_{\mathrm{nozzle}}/H$, where $H = 0.2$ mm is the gap distance between the nozzle tip and the substrate. Actuation strains were measured by optical microscopy.

(2) Validation of the Couette flow approximation via volumetric flow rate measurements (Fig. R6)

To validate the assumption that director alignment is governed by shear between the nozzle tip and the substrate rather than by extensional flow or stretching of the extrudate, we measured the volumetric flow rate (Q) under two conditions: free extrusion from the nozzle (Q_{nozzle}) and during active printing (Q_{print}) (**Fig. R6a–c**).

At P = 500 kPa, Q_{print} was consistently lower than Q_{nozzle} across the entire range of $v_{\mathrm{nozzle}} = 2$–20 mm s^{-1} (Fig. R6c). Although Q_{print} approaches Q_{nozzle} at higher translation speeds, it remains below Q_{nozzle} throughout the entire range tested, confirming that the overfed regime is maintained under all printing conditions used in this study. This indicates that the volumetric supply from the nozzle exceeds the amount the substrate can carry away under the given printing conditions. As a result, we conclude that the dominant shear experienced by the smectic ink occurs in the gap between the nozzle tip and the substrate, justifying the use of the Couette approximation for shear rate estimation.

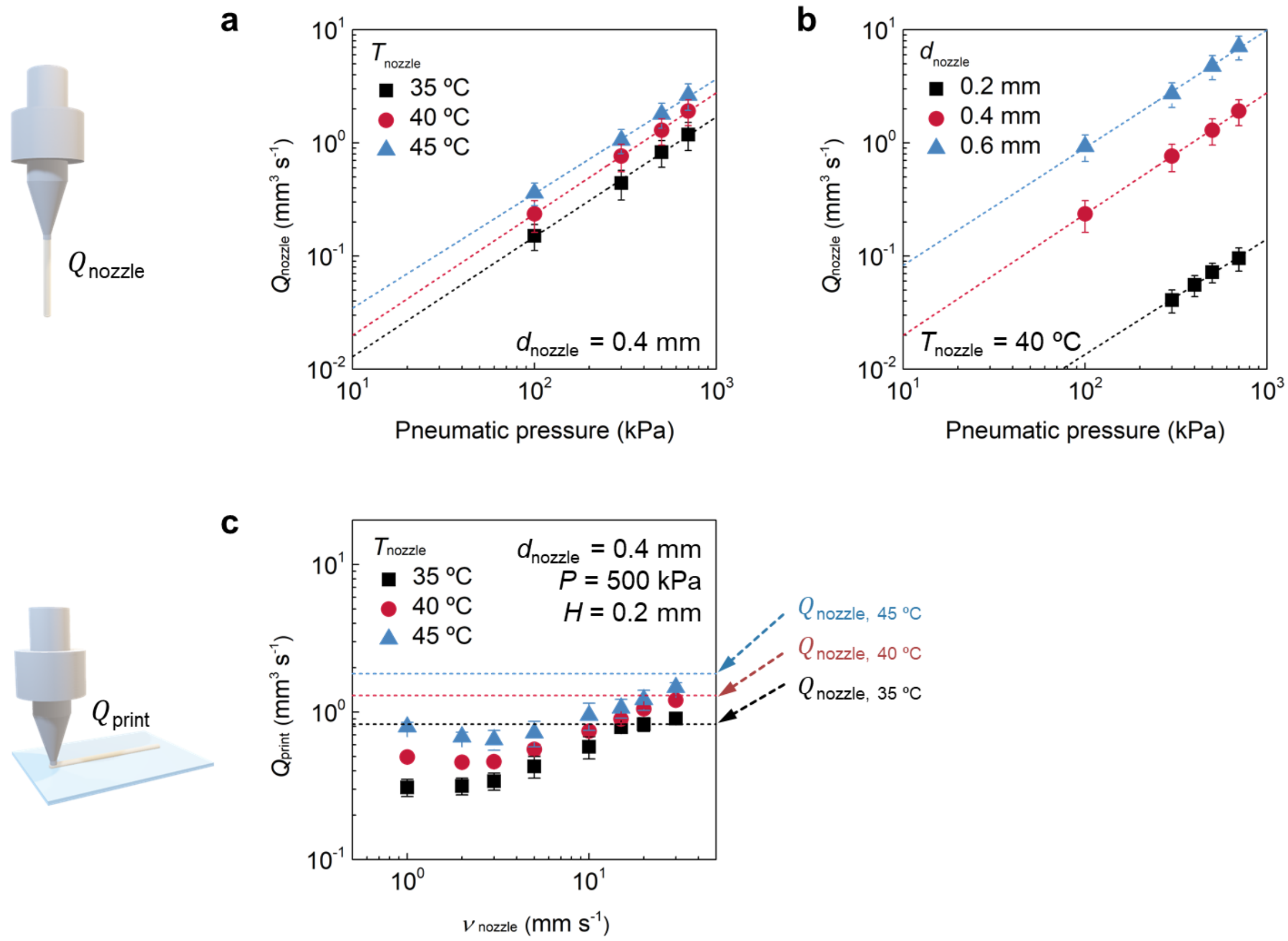

Figure R6. The volumetric flow rate (Q) of smectic ink during free extrusion (Q_{nozzle}) and during printing (Q_{print}). (a) Q_{nozzle} as a function of applied pneumatic pressure (P) at different print temperatures (T_{nozzle}) using a 0.4 mm diameter nozzle. (b) Q_{nozzle} as a function of P for different nozzle diameters (d_{nozzle}). (c) Q_{print} as a function of nozzle translation speed (v_{nozzle}) at different T_{nozzle} under the conditions H = 0.2 mm, P = 500 kPa, and d_{nozzle} = 0.4 mm. Dashed lines indicate the corresponding Q_{nozzle} values at P = 500 kPa for each temperature, shown as reference to highlight that Q_{print} remains below Q_{nozzle} across all tested v_{nozzle} conditions. All Q values were measured using a gravimetric method.

The above discussion has been included in the revised manuscript as follows, and in **Supplementary Note 2** and **Supplementary Fig. 5** of the revised Supplementary Materials.

"Printing was performed using a nozzle with an inner diameter (d_{nozzle}) of 0.4 mm and a print height (H) of ~0.2 mm. Two key parameters, nozzle temperature (T_{nozzle} = 35–45 °C) and nozzle translation speed (v_{nozzle} = 2–30 mm s^{-1}), were systematically varied, while print pressure and bed temperature were maintained constant at 500 kPa and ~25 °C, respectively (Fig. 2c–d). The printing geometry, where d_{nozzle} > H, allows the volumetric supply of the ink from the nozzle to exceed the amount that can be carried away by the substrate under the given printing conditions (Supplementary Note 2 and Supplementary Fig. 5). This ensures that director alignment in the printed filament is primarily controlled by shear flow between the nozzle tip and the substrate, rather than by extensional flow or stretching of the extrudate [25,40]." (**Page 6, lines 128–136**)

"To further elucidate this transition, the alignment states obtained from WAXS measurements were mapped as a function of print temperature and speed (Fig. 2g). The shear rates ($\dot{\gamma}$) applied during

the print process on the x-axis were estimated using a simple Couette flow approximation:

$$\dot{\gamma} = \frac{velocity\ difference\ between\ plates}{distance\ seperating\ plates} = \frac{v_{nozzle}}{H}$$

In this study, $H = 0.2$ mm; therefore, $v_{nozzle} =$ [2, 3, 5, 10, 15, 20, 30] mm s^{-1} generates shear rates of $\dot{\gamma}$ = [10, 15, 25, 50, 75, 100, 150] s^{-1}, respectively." (**Page 7, lines 159–169**)

(3) Shear-rate-governed orientation inversion (Fig. R7)

To further validate that the shear rate, rather than v_{nozzle} or H individually, is the primary parameter governing alignment, we independently varied the nozzle diameter (d_{nozzle}) and gap height (H) while maintaining constant conditions of P = 500 kPa and T_{nozzle} = 40 °C. As shown in the actuation strain heat maps (**Fig. R7**), increasing H shifted the critical v_{nozzle} required for the orientation switching transition to higher values. Importantly, this trend was consistent across both nozzle diameters tested (0.4 mm and 0.6 mm). When expressed in terms of shear rate ($\dot{\gamma}$), the transition from perpendicular to parallel director alignment occurred approximately within $\dot{\gamma}$ = 33–67 s^{-1} across all tested geometric conditions, regardless of the specific combination of v_{nozzle} and H used. This transition range closely corresponds to the onset of the second shear-thinning regime in the steady-shear viscosity curve ($\dot{\gamma} \approx$ 40–60 s^{-1}), **highlighting that shear-induced orientation flipping in the smectic phase is strongly governed by the shear-rate regime associated with the second shear-thinning region**.

.

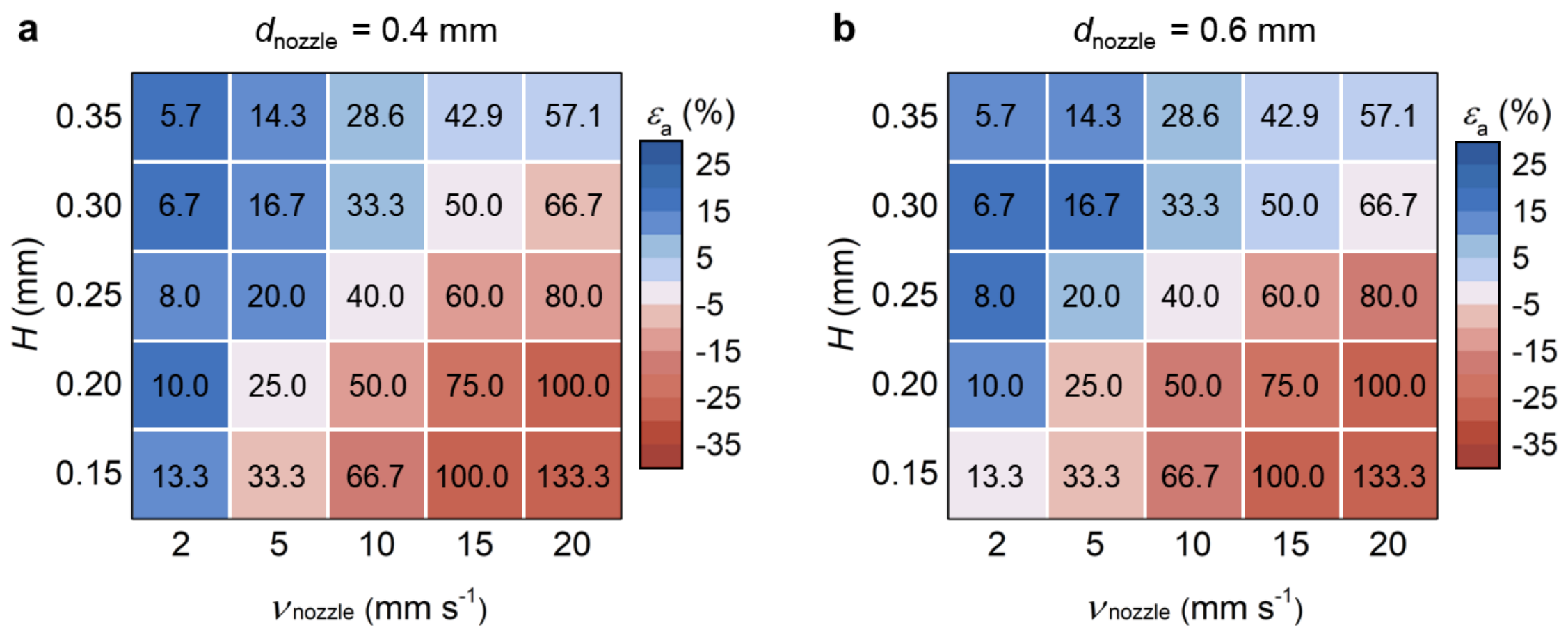

Figure R7. Heat map of actuation strain at 100 °C for filaments printed at varying nozzle translation speeds (v_{nozzle}) and gap distances (H) under constant conditions of P = 500 kPa and T_{nozzle} = 40 °C, using 0.4 mm (a) and 0.6 mm (b) diameter nozzles. The values shown in the map represent the applied shear rates ($\dot{\gamma} = v_{nozzle}/H$) at each condition.

We have added these results and discussions in the revised manuscript on **Page 10, lines 271–283**, and in **Supplementary Fig. 19** of the revised Supplementary Materials.

"Shear rate and temperature are the primary parameters regulating alignment and thermal actuation, both of which are closely associated with the steady-shear viscosity behaviour of the ink. To

validate the cross-platform generality of our approach, we further examined the effects of d_{nozzle}, H, and ink viscosity on alignment and actuation behaviour. Supplementary Fig. 19 presents actuation strain heat maps as a function of v_{nozzle} and H for two nozzle diameters (0.4 and 0.6 mm) under constant conditions of T_{nozzle} = 40 °C and P = 500 kPa. Regardless of d_{nozzle}, increasing H shifted the critical v_{nozzle} required for orientation switching to proportionally higher values, consistent with the Couette flow approximation, $\dot{\gamma} = v_{nozzle} / H$. When expressed in terms of $\dot{\gamma}$, the perpendicular-to-parallel director transition occurred within a consistent range of 33–67 s^{-1} across all tested v_{nozzle} and H combinations, in good agreement with the onset of the second shear-thinning regime in the steady-shear viscosity curve. These results confirm that shear-induced director inversion is governed by the applied $\dot{\gamma}$ rather than by v_{nozzle} or H individually, establishing the cross-platform generality of our processing principle."

(4) COMSOL CFD simulations of in-nozzle shear rate distribution

We conducted computational fluid dynamics (CFD) simulations using COMSOL Multiphysics to elucidate the shear rate distribution experienced by the ink inside the nozzle (**Fig. R8**). The CFD analysis was performed under the representative condition of T_{nozzle} = 45 °C, at which the steady-shear viscosity of the ink exhibits distinct single shear-thinning behavior (**Fig. R3**). We note that accurately describing the in-nozzle shear rate for the smectic ink, which exhibits double shear-thinning behavior, would require a more sophisticated constitutive model; this is beyond the scope of the present study.

Using the internal geometry of the 0.4 mm diameter nozzle (**Fig. R8a**) and the experimentally measured volumetric flow rate of Q_{nozzle} = 1.79 mm^3 s^{-1} at P = 500 kPa, we obtained the velocity magnitude map shown in **Fig. R8b**. One-dimensional velocity and shear rate profiles were extracted as a function of radial position (r_i) at two axial positions: z = 0 mm (nozzle outlet) and z = 0.63 mm (entry to the cylindrical region) (**Fig. R8c–e**). At both positions, the velocity decreases sharply near the nozzle wall, with a corresponding marked increase in local shear rate in this near-wall region.

However, the central region of the nozzle (r_i < 0.15 mm) exhibits shear rates below 50 s^{-1}, which are insufficient to exceed the empirically determined critical shear rate range for orientation switching ($\dot{\gamma}$ ≈ ~40–60 s^{-1}). Furthermore, as these simulations were performed under fully isotropic conditions, where the ink viscosity is substantially lower than that in the smectic phase, the actual shear rates experienced by the smectic ink inside the nozzle are expected to be even lower. Taken together, these results confirm that nozzle extrusion alone, without additional shear imposed in the nozzle-to-substrate gap, is insufficient to uniformly and effectively control director orientation under our printing geometry and conditions.

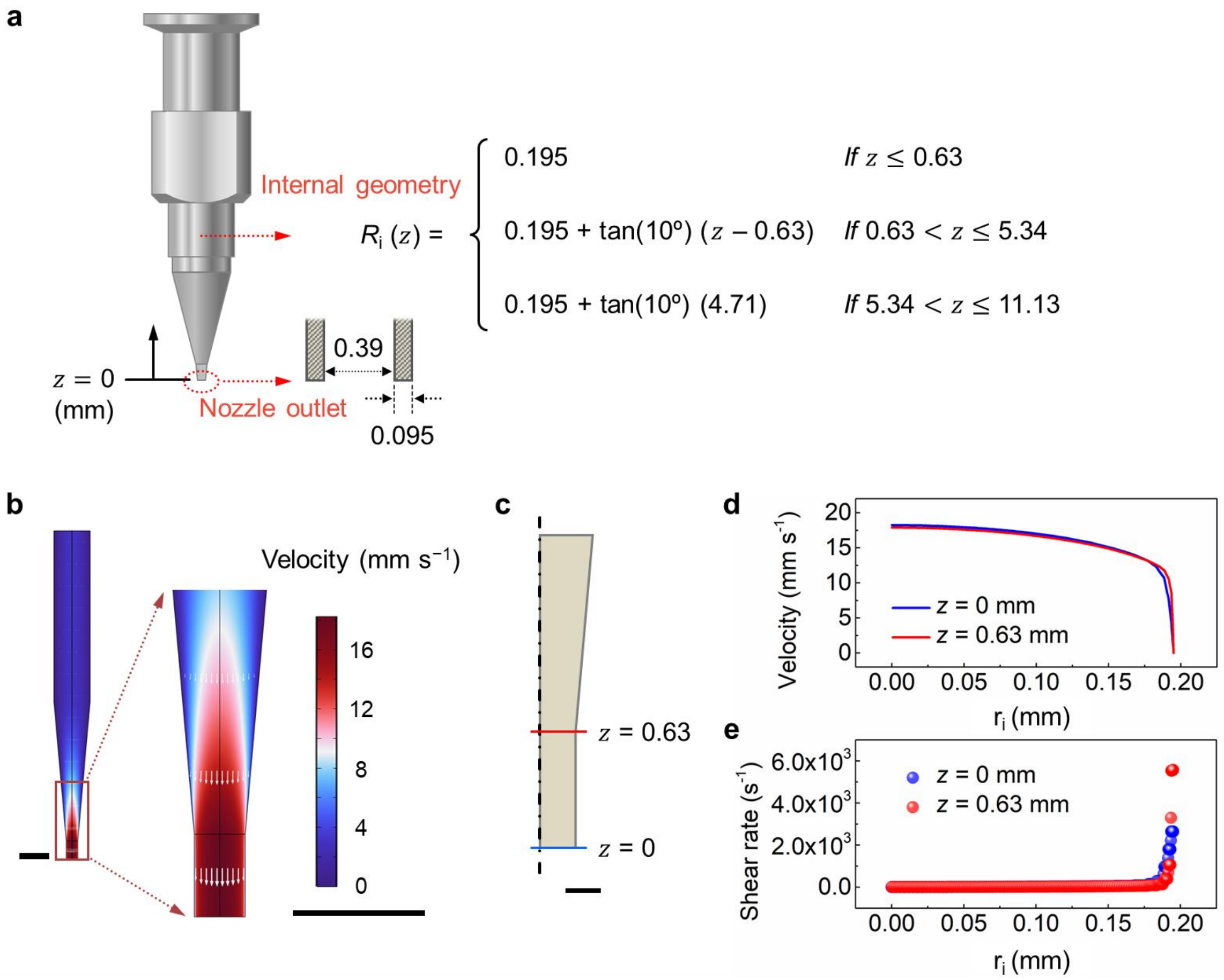

Figure R8. Velocity and shear rate analysis of the ink inside the nozzle under the processing conditions of d_{nozzle} = 0.4 mm, P = 500 kPa, and T_{nozzle} = 45 °C. (a) Internal geometry of the 0.4 mm diameter nozzle defined by a piecewise function $R_i(z)$. (b) Velocity magnitude map of the ink inside the nozzle obtained from COMSOL simulations. Scale bars = 10 mm. (c) Axial locations corresponding to the nozzle outlet (z = 0 mm) and the entry to the cylindrical region (z = 0.63 mm). Scale bar = 0.2 mm. (d) 1D velocity and (e) shear rate profiles as a function of radial position (r_i) extracted at z = 0 mm and z = 0.63 mm.

(5) Molecular weight-dependence of smectic ink on shear alignment and actuation

Finally, we investigated the effects of molecular weight on the alignment and actuation behavior of the smectogenic ink (**Fig. R9–R10**). To this end, we synthesized a smectogenic ink with the same composition and T_i but with a higher molecular weight ($M_n \approx 5.0$ kDa) than the original ink ($M_n \approx 3.8$ kDa) (**Fig. R9 and Fig. R10a**), and characterized its temperature-dependent steady-shear viscosity and alignment behavior. As shown in **Fig. R10b**, the overall steady-shear viscosity increased by approximately 4–5 times compared with that of the original ink, and the double shear-thinning behavior shifted to lower shear rates. In the smectic phase, the viscosity curve exhibited an initial shear-thinning regime at low shear rates ($0.03 < \dot{\gamma} < 1$ s^{-1}), a pseudo-Newtonian regime at intermediate rates ($1 < \dot{\gamma} < 10$ s^{-1}), and a second shear-thinning regime at higher shear rates ($\dot{\gamma} > 10$ s^{-1}). Notably, the critical shear rate for alignment switching at $T_{nozzle} \approx 40$ °C shifted to lower values (10–20 s^{-1}) relative to that of the original ink (50–75 s^{-1}), and this trend was consistently reflected in the actuation strain heat map

(**Fig. R10c**). This behavior can be attributed to the longer polymer chains, which impose greater steric constraints on interlayer slipping than shorter chains, thereby rendering the smectic layer structure more susceptible to shear-induced disruption at lower shear rates. Furthermore, these results demonstrate that the processing window for switchable alignment behavior in smectic LCEs can be identified and predicted from the relationship between the applied shear rate and the steady-shear viscosity profile, providing a general design principle for shear-rate-controlled director programming.

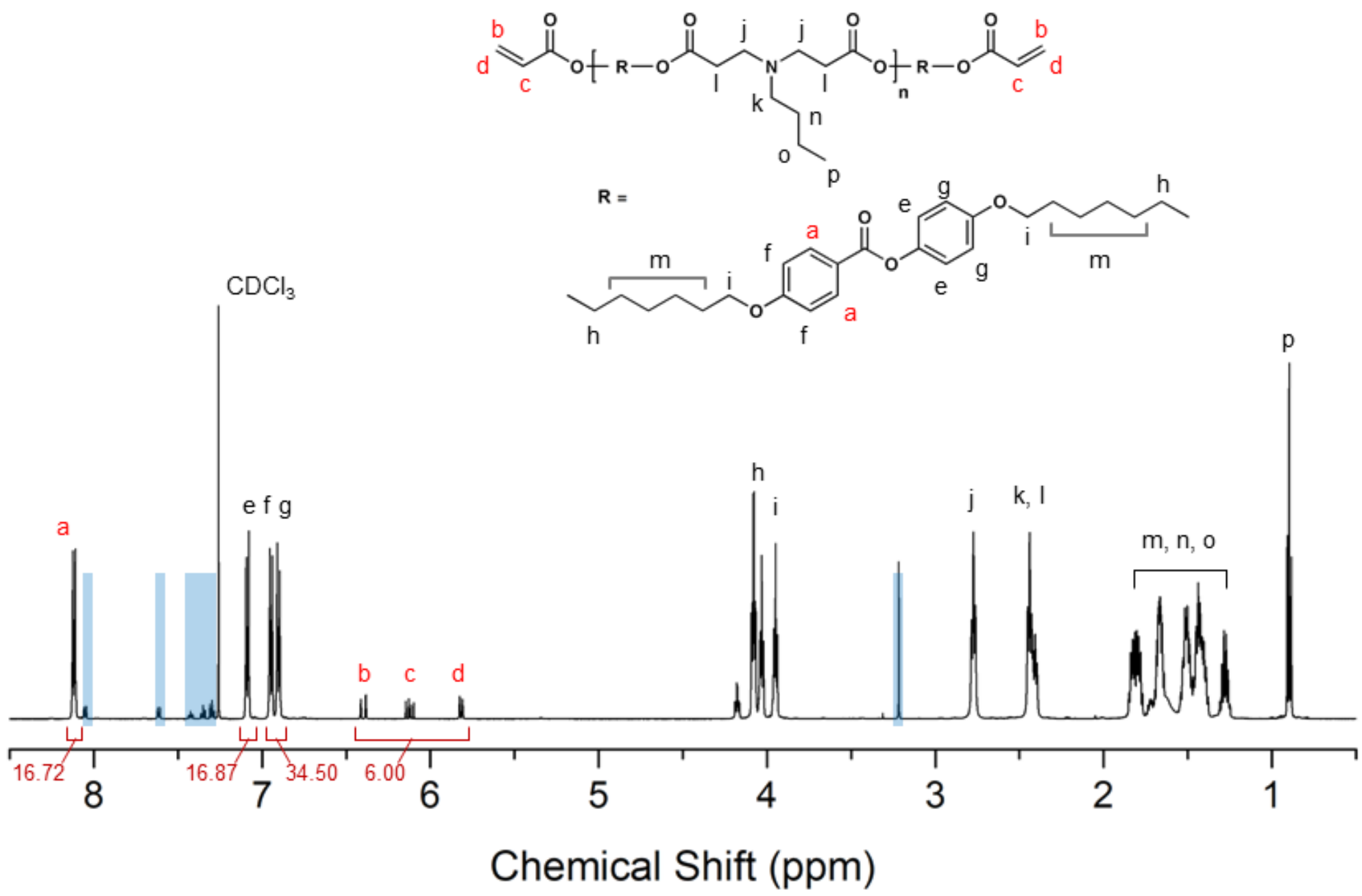

Figure R9. ^{1}H NMR spectra of the smectogenic ink with higher molecular weight (MW) in $CDCl_3$. The increased M_n was achieved by allowing further propagation of the ink via aza-Michael addition for 30 days at room temperature following synthesis. The light blue boxes highlight the peaks related to the photoinitiator (Irgacure 651) in the ink. The three peaks between 5.85 and 6.45 ppm (*b, c, d*) correspond to the six protons in the diacrylate end groups of the oligomer (CH_2=CHCOO–). The integration value of these peaks, set to six, was used to calibrate the other peaks. The peak at 8.12 ppm (*a*) corresponds to the two aromatic protons of the oligomer, with an integration of 16.72. From these values, the degree of polymerization (DP) was calculated as (16.72/2) – 1 = 7.36. The number-average molecular weight was then estimated as M_n = DP × M_n (repeating unit) + M_n (end groups) ≈ 7.36× 611.77 + 538.60 ≈ 5000 Da.

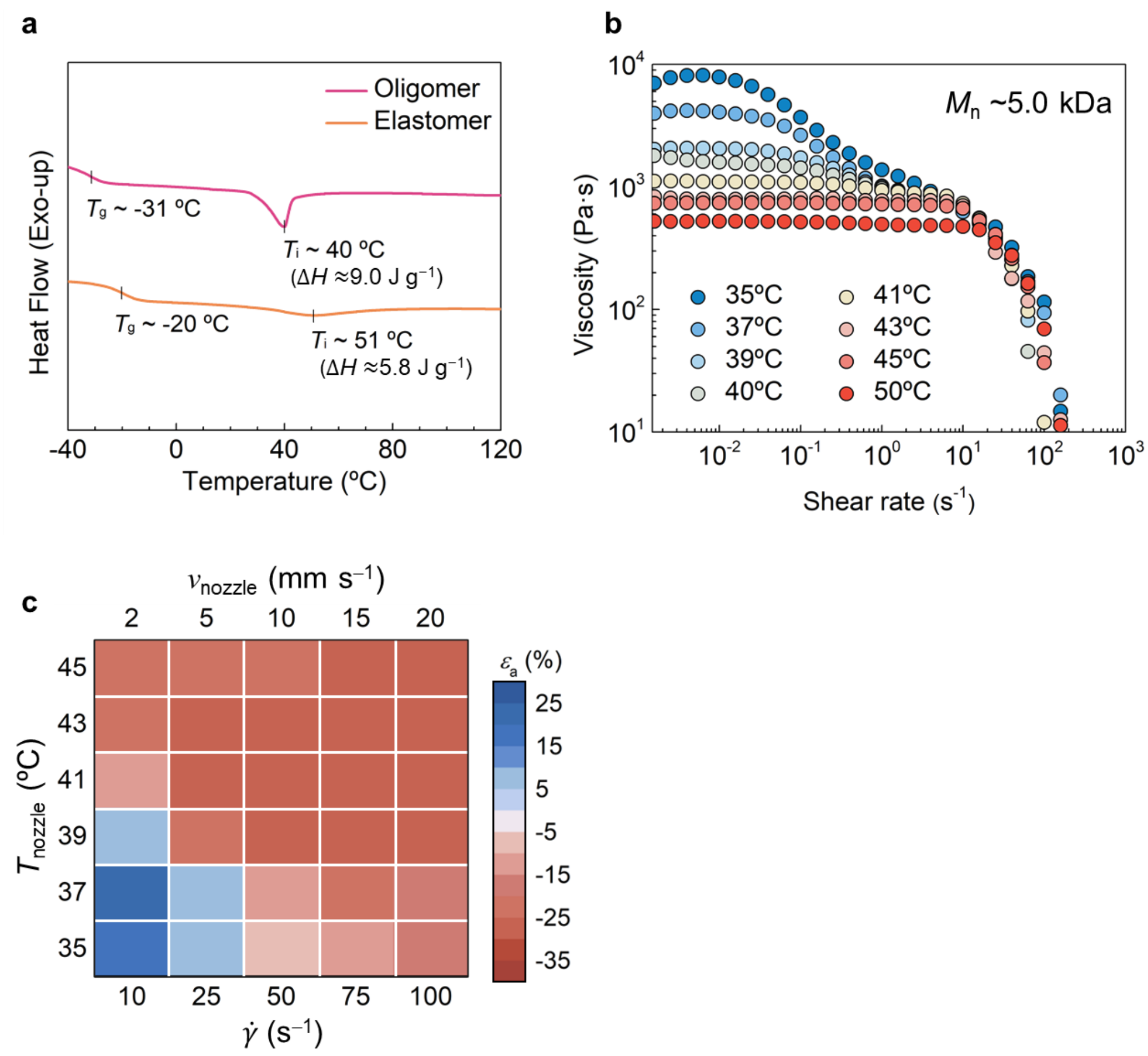

Figure R10. Influence of the molecular weight (MW) of the smectogenic ink on its alignment and actuation behavior. (a) Differential scanning calorimetry (DSC) curves of smectogenic ink (oligomer, M_n ~5.0 kDa) and cured LCE during the second heating cycle. Measurements were performed between –50 °C and 150 °C at a rate of 10 °C min^{-1} under nitrogen. (b) Steady-shear viscosity as a function of shear rate at various temperatures for the oligomer. (c) Heat map of actuation strain at 100 °C for filaments printed at different temperatures and speeds, measured by optical microscopy. The shear rates ($\dot{\gamma}$) on the bottom axis are calculated from the corresponding v_{nozzle} values as $\dot{\gamma} = v_{nozzle}/H$, where H = 0.2 mm. The actuation strain, $\varepsilon_a(\%)$, is defined as $\varepsilon_a = (l - l_0)/l_0 \times 100$, where l_0 and l are the filament length at 30 °C and 100 °C, respectively.

We have added these results and discussions in the revised manuscript on **Page 11, lines 284–298**, and in **Supplementary Figs. 20–21** of the revised Supplementary Materials.

"To examine the effect of ink viscosity, we synthesized a smectogenic ink with the same composition and T_i but higher molecular weight (M_n ≈ 5.0 kDa) than the original ink (M_n ≈ 3.8 kDa) (Supplementary Figs. 20–21). As shown in Supplementary Fig. 21, the steady-shear viscosity increased approximately 4–5 folds, and the double shear-thinning behaviour shifted to lower shear rates. In the smectic phase, the viscosity curve showed initial shear thinning at low shear rates ($0.03 < \dot{\gamma} < 1$ s^{-1}), a pseudo-Newtonian regime at intermediate rates ($1 < \dot{\gamma} < 10$ s^{-1}), and a second shear-thinning regime at higher rates ($\dot{\gamma} > 10$ s^{-1}). Accordingly, the critical shear rate for alignment switching shifted to lower values

(10–25 s^{-1}) relative to that of the original ink (50–75 s^{-1}), as reflected in the actuation strain heat map (Supplementary Fig. 21). This shift is attributed to the longer polymer chains, which impose greater constraints on interlayer slipping and render the smectic layers more susceptible to shear-induced disruption, leading to shear thinning at lower shear rates. [48,49] Taken together, these results demonstrate that the processing window for switchable alignment can be predicted from the relationship between the applied shear rate and the steady-shear viscosity profile, providing a generalizable design principle for shear-rate-controlled director programming."

5. The "mixed" alignment regime near the phase transition is attributed to orientation gradients across the filament thickness, but this remains speculative. Please provide spatially resolved characterization, such as microbeam WAXS mapping, polarized optical/ Raman microscopy, or cross-sectional analysis to directly confirm the through-thickness or lateral director variation.

Response:

We thank the reviewer for this insightful comment. To directly characterize the alignment distribution across the film thickness, we performed grazing-incidence wide-angle X-ray scattering (GI-WAXS) on printed LCE films (**Fig. R11**). Three representative samples were selected: (1) printed at 35 °C and 5 mm s^{-1} (T35V5, perpendicular alignment), (2) 40 °C and 10 mm s^{-1} (T40V10, mixed state), and (3) 45 °C and 20 mm s^{-1} (T45V20, parallel alignment).

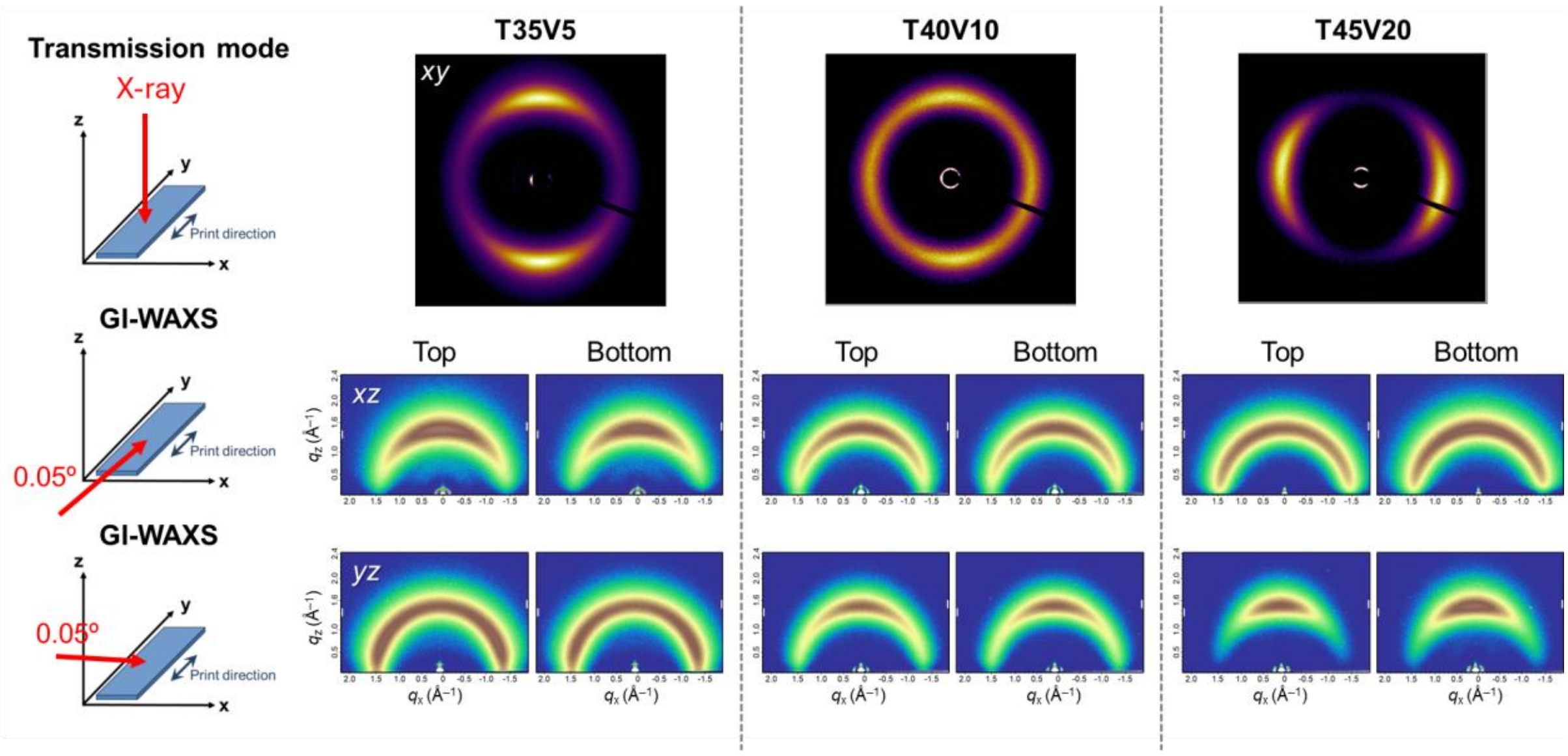

Figure R11. 2D WAXS images obtained from transmission mode and grazing-incidence mode for three representative samples: (1) printed at 35 °C and 5 mm s^{-1} (T35V5, perpendicular alignment), (2) 40 °C and 10 mm s^{-1} (T40V10, mixed state), and (3) 45 °C and 20 mm s^{-1} (T45V20, parallel alignment). 2D GI-WAXS patterns were acquired at a grazing incidence angle of 0.05°.

The GI-WAXS patterns provide molecular orientation information near the top and bottom surfaces of each film. The orientation trends observed by GI-WAXS are consistent with the transmission-mode WAXS results, confirming that the bulk alignment state is representative of both surfaces. Notably, for the mixed-orientation sample (T40V10), no significant difference in alignment

direction was observed between the top and bottom surfaces. This result indicates that our original interpretation, namely that the mixed orientation arose from differential alignment across the film thickness due to velocity or temperature gradients, was not supported by the spatially resolved data, and we have revised this discussion accordingly in the manuscript.

We now propose a revised interpretation: because the shear rate is effectively uniform across the film thickness and the relaxation time of the LC oligomer is considerably slower than that of low-molecular-weight liquid crystals, the observed mixed-orientation pattern likely originates from a transient orientational state. That is, near the transition regime between perpendicular and parallel alignment, the slow relaxation dynamics of the oligomeric LC result in an intermediate, kinetically trapped orientation rather than a discrete through-thickness gradient.

Accordingly, we have revised the manuscript as follows:

Original sentence:

~~"The emergence of mixed patterns arises from differential alignment across the film thickness, where the director orientation near the phase transition becomes highly sensitive to subtle shear and temperature gradients."~~

Revised sentence:

"We speculate that the emergence of mixed patterns near the transition regime arises from the slow relaxation dynamics of the LC oligomer compared to low–molecular weight liquid crystals. In this regime, the orientation distribution appears as a coexistence of perpendicular and parallel alignment rather than a purely discrete orientation." (**Page 7, lines 159–162**)

6. The presented actuation tests lack long-term cycling data. For assessing functional robustness, please report strain retention or hysteresis after multiple (e.g., >100) heating–cooling cycles.

Response:

We thank the reviewer for this constructive comment. To evaluate actuation durability, we conducted cyclic actuation tests on a representative smectic LCE film printed at 35 °C and a speed of 5 mm s^{-1} (**Figure R12**). The film was subjected to 100 thermal actuation cycles over a temperature range of 10–100 °C. After 100 cycles, the maximum actuation strain showed only a marginal change of approximately 1.5% (**Figure R12a**). As the cycle number increased, the slope of the actuation curves became slightly steeper (**Figure R12b**). This change in cycling test may be attributed to structural relaxation or minor reorganization of crosslinking points during repeated thermal cycling, thereby progressively reducing constraints on mesogen motion within the network. Importantly, the actuation strain remained stable throughout all 100 cycles, with no significant degradation in performance. Overall, these results confirm good actuation durability of the printed smectic LCE under repeated thermal actuation.

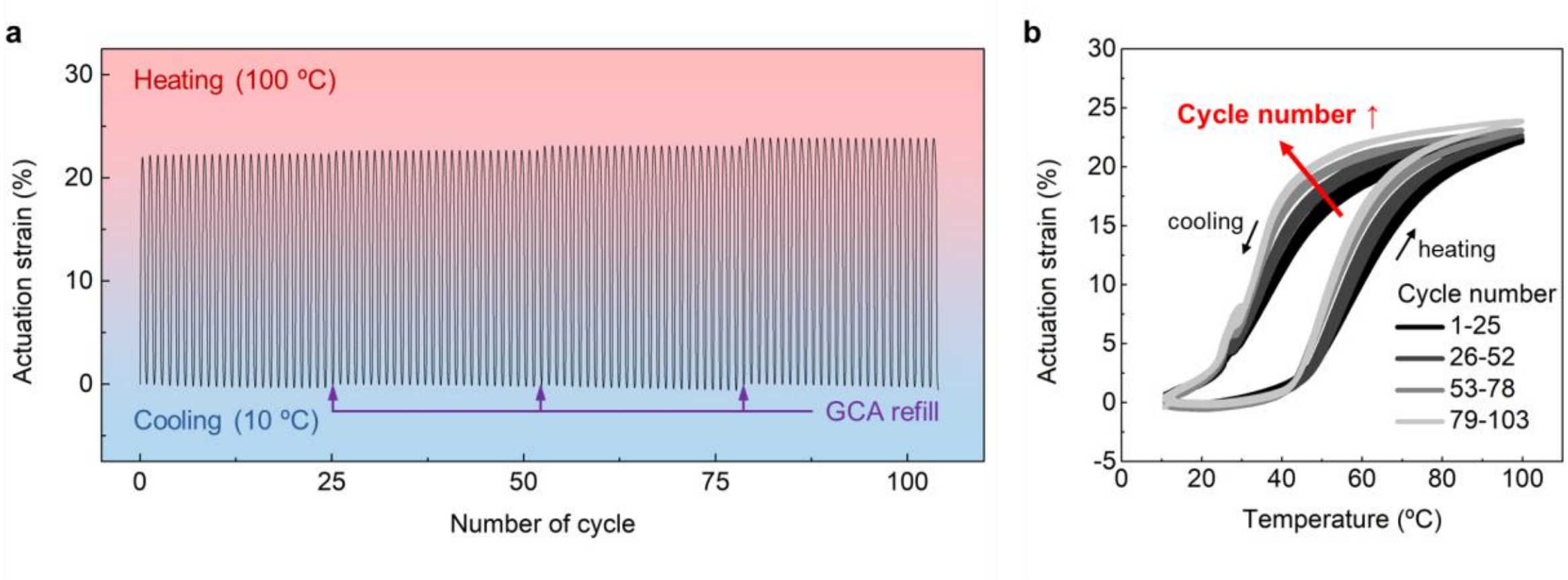

Figure R12. Actuation stability of the LCE film printed at T_{nozzle} = 35 °C and v_{nozzle} = 3 mm s^{-1}. (a) Thermal actuation strain measured over 103 heating–cooling cycles between 10 and 100 °C at a rate of 5 °C min^{-1} under a constant applied stress of 1 kPa. Approximately 25 cycles could be performed with a fully filled gas cooling accessory (GCA). The GCA was refilled with liquid nitrogen before the 26th, 53rd, and 78th cycles to enable testing over 100 heating–cooling cycles. (b) Thermal actuation strain as a function of temperature during cyclic test. As the cycle number increased, the slope of the actuation curves became steeper, while the maximum actuation strain changed only slightly, from 22.3% to 23.9% after 103 cycles.

These results and the relevant discussion are included in **Fig. 4h, Supplementary Fig. 23**, and the revised manuscript (**Page 11, lines 303–307**).

“The cyclic stability of the printed LCE was assessed under an isostress mode using a representative elongating LCE printed at T_{nozzle} = 35 °C and v_{nozzle} = 3 mm s^{-1} (Fig. 4h and Supplementary Fig. 23). The printed LCE was subjected to 100 thermal actuation cycles over 10–100 °C, during which the actuation strain remained stable, showing only a marginal change of approximately 1.5% and no significant performance degradation.”

Reviewer #2 (Remarks to the Author):

This study reports a DIW strategy that enables shear- and temperature-dependent orientation switching in smectic LCE inks. By tuning the print temperature and speed, the mesogen orientation can be switched between perpendicular and parallel alignment within a single filament, which allows both contractile and elongational actuation modes to coexist within a single material system. The approach successfully demonstrates the printing of LCE structures capable of complex shape transformations.
I have the following questions and comments:

1. It remains unclear how the non-equilibrium molecular dynamics (NEMD) simulation results contribute to understanding the DIW mechanisms. The simulations operate at vastly different spatial and temporal scales from the experiments, and there is no direct comparison between the simulated outcomes and the experimental measurements. The authors are suggested to clarify the intended purpose of the simulations, whether they are primarily conceptual or quantitatively predictive, and to discuss the limitations associated with the significant disparity in scales. Without clear validation or correlation, it is difficult to assess how the simulated insights can directly guide experimental understanding or process optimization.

Response:

We agree with the reviewer that the NEMD simulations operate at different spatial and temporal scales than the experiments. However, the purpose of the NEMD simulations is to gain insight into the reorientation of mesogens under shear flow and to elucidate the molecular mechanisms underlying the observed orientation flipping; thus, we expect only qualitative agreement. Nonetheless, we take this opportunity to present additional simulation results that explicitly demonstrate qualitatively similar behavior to that observed in the WAXS and rheology experiments.

In the NEMD simulations mapped to the WAXS experiment, when both the beam direction and the velocity-gradient direction are along $\hat{z}$, the in-plane structure appears in the flow–vorticity (x–y) plane. This contrasts with the experimental orientation, where the print direction lies along $\hat{y}$ rather than $\hat{x}$ as in the simulations (see **Supplementary Fig. 7**). Here, we focus on the similarities between the two and adopt the coordinate system used in the NEMD simulations.

If we consider only the mesogen end-groups as scatterers, the scattering function detects the in-plane structures as beads arranged within a plane. To obtain these scattering functions, we averaged over 100 independent simulations. The 3D scattering function is computed from the Fourier transform of the end-group density:

$$S(\mathbf{q}) = S(q_x, q_y, q_z) = \frac{1}{N}\left\langle \sum_{j=1}^{N}\sum_{k=1}^{N} exp\left(i\,\boldsymbol{q}\cdot(\boldsymbol{r}_j - \boldsymbol{r}_k)\right)\right\rangle,$$

where $\mathbf{q} = (q_x, q_y, q_z)$, $\mathbf{r}_j$ is the position of the j-th mesogen end-group, and $\langle\cdots\rangle$ denotes an ensemble average over the 100 simulation snapshots.

To compare with the WAXS geometry, in which the beam is along $\hat{z}$ and the detector records

the in-plane q_x–q_y components, the 3D scattering intensity is projected onto the detector plane by evaluating:

$$S(q_x, q_y) = S(q_x, q_y, q_z = 0),$$

i.e., the slice of reciprocal space perpendicular to the beam direction. This yields the 2D scattering function in the flow–vorticity plane.

We observe similar behavior in the resulting scattering patterns as the shear rate increases. At low shear rates, higher intensities appear at $(0, \pm q^*, 0)$, indicating a perpendicular orientation of the smectic layers. With increasing shear, a transition and coexistence of perpendicular and parallel orientations emerges, followed at higher shear rates by a predominantly parallel orientation, evidenced by strong intensities at $(\pm q^*, 0, 0)$.

It should be noted that the X-ray measurements capture only the final structure formed after shear application, followed by cooling and curing, during which the smectic layers are re-established. Therefore, the experimental WAXS images show smectic layer structures in the printed samples even under high-shear-rate printing conditions. By contrast, the simulations track the instantaneous configurations of individual mesogens under flow, revealing the loss of smectic layering at high shear rates. When these smectic layers disappear, the characteristic scattering ring with its high-intensity lobes also vanishes and is replaced by the scattering signature of a nematic state: two elongated, vertically oriented intensity maxima centered at $(\pm q^*, 0, 0)$, accompanied by diffuse scattering along the q_y direction. This pattern reflects strong alignment of mesogens along the flow direction with no remaining positional layer order.

We have added the lines to **Supplementary Note 3** of the revised Supplementary Materials:

"The sequence of scattering functions in the (q_x, q_y) *plane in* ***Figure R13 (Supplementary Figure 16)*** *shows the evolution of mesogen ordering under increasing shear rate in the NEMD simulations. At the lowest shear rate (leftmost panel), the pattern displays a nearly isotropic ring of intensity with weak maxima at* $(0, \pm q^*)$*, characteristic of a perpendicular smectic orientation, where the layer normal lies along the vorticity direction. As the shear rate increases, these perpendicular-orientation maxima grow in intensity and become more distinct (second panel).*

With further shear (third and fourth panels), the system enters a coexistence regime: the original peaks at $(0, \pm q^*)$*, associated with the perpendicular smectic state; persist, while new intensity maxima emerge at* $(\pm q^*, 0)$*, indicating the onset of a parallel orientation in which the smectic layer normal aligns with the flow direction. The coexistence of both sets of peaks reflects a shear-driven reorientation transition between perpendicular and parallel smectic organizations.*

At the highest shear rate (rightmost panel), the circular ring associated with smectic layering disappears entirely, signaling the loss of positional layer order. The scattering collapses into two strong, elongated vertical lobes centered at $(\pm q^*, 0)$*, producing a pattern characteristic of a nematic state with strong mesogen alignment along the flow direction and no remaining smectic periodicity."*

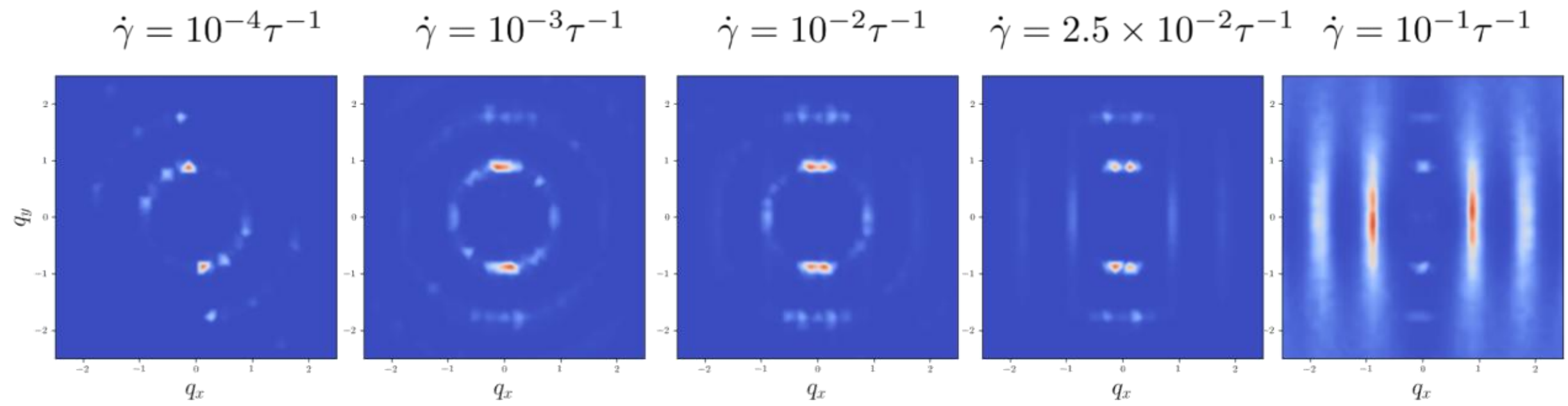

Figure R13. Scattering functions in the (q_x, q_y) plane from NEMD simulations across increasing shear rates. Left to right: low to high shear rate. At low shear, the scattering displays a ring with intensity maxima at $(0, \pm q^*)$, characteristic of a perpendicular smectic orientation. As shear increases, additional maxima appear at $(\pm q^*, 0)$, indicating the coexistence of perpendicular and parallel smectic orientations. At the highest shear rate, the smectic ring disappears and the scattering transitions to two elongated intensity lobes at $(\pm q^*, 0)$, reflecting a nematic state with strong flow-aligned mesogens and loss of smectic layer order.

Furthermore, we added calculations of the shear-dependent viscosity obtained from the NEMD simulations to provide additional comparison with the rheology experiments. The following lines have been added to the **Supplementary Note 3**:

"***Figure R14 (Supplementary Figure 17)*** *shows the shear-dependent viscosity* $\eta(\dot{\gamma})$ *obtained from NEMD simulations under steady simple shear. For each shear rate* $\dot{\gamma}$*, the viscosity is computed from the averaged shear stress,*

$$\eta(\dot{\gamma}) = \frac{\langle \tau_{xz} \rangle}{\dot{\gamma}},$$

where $\langle \tau_{xz} \rangle$ *is the time- and ensemble-averaged* xz *component of the stress tensor, averaged over 100 independent simulations. The resulting viscosity curve exhibits two shear-thinning regimes, similar to the rheology experiments, each corresponding to a distinct structural transition observed in the scattering patterns.*

At low shear rates, the system displays a perpendicular smectic orientation, resulting in relatively high viscosity. As the shear rate increases, the viscosity decreases smoothly, marking the first shear-thinning regime, coinciding with the transition from perpendicular orientation to a coexistence of perpendicular and parallel smectic domains. At higher shear rates, a second shear-thinning regime emerges, associated with the system adopting a predominantly parallel orientation and ultimately a nematic state with fully flow-aligned mesogens. This structural reorganization reduces resistance to flow, producing the characteristic double-sloped shear-thinning behavior shown in the ***Figure 2b****.*"

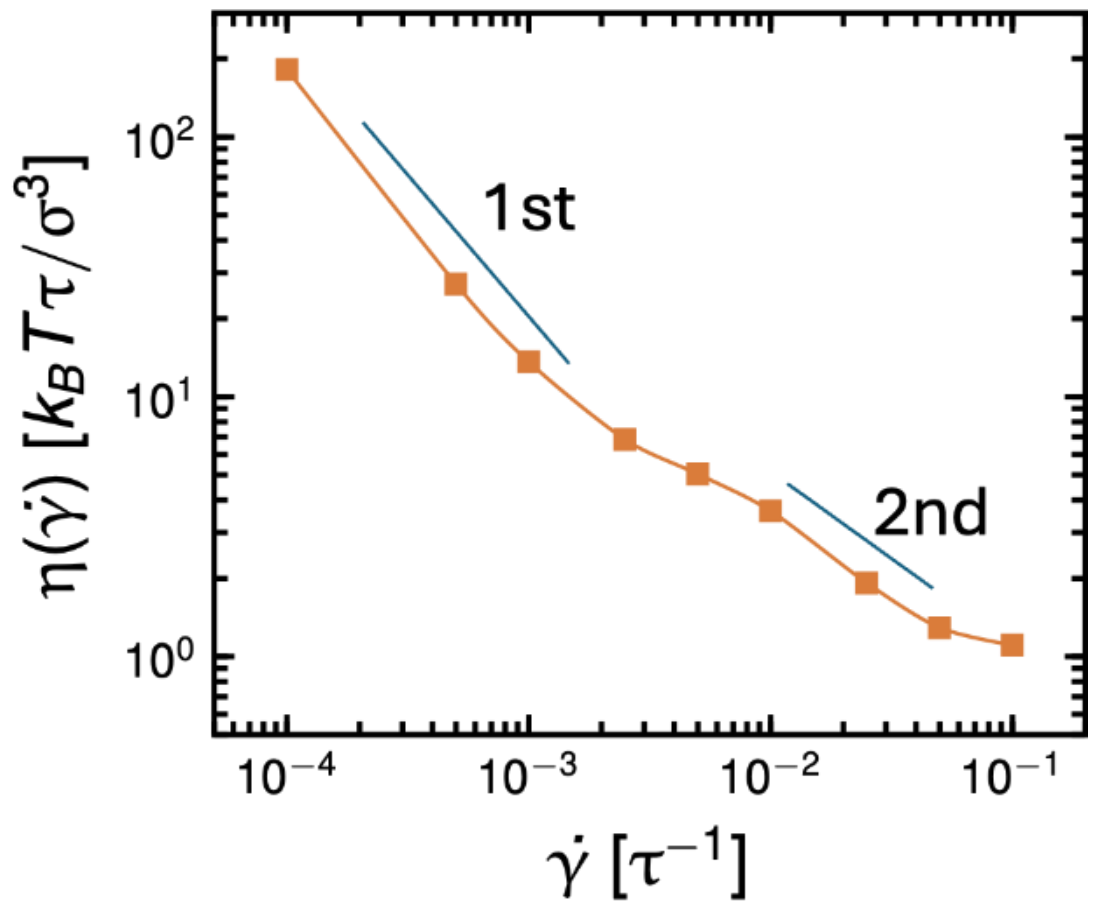

Figure R14. Shear-dependent viscosity $\eta(\dot{\gamma})$obtained from NEMD simulations under steady simple shear. For each shear rate $\dot{\gamma}$, the viscosity is calculated from the averaged shear stress according to $\eta(\dot{\gamma}) = \langle \tau_{xz} \rangle / \dot{\gamma}$, where $\langle \tau_{xz} \rangle$ is the time- and ensemble-averaged xz component of the stress tensor, averaged over 100 independent simulations. The resulting viscosity curve displays two distinct shear-thinning regimes, similar to the behavior observed experimentally in rheology measurements. Each shear-thinning regime corresponds to a structural transition identified in the scattering patterns, from perpendicular smectic alignment to coexistence of orientations, and ultimately to a predominantly parallel (nematic) orientation at high shear rates.

Collectively, these results and the relevant discussion are included in the revised manuscript on **Page 9, lines 233–242** as follows:

*"2D scattering functions in the flow–vorticity plane and viscosity plots as a function of shear rate are obtained from NEMD simulations (**Supplementary Figs. 16–17**). Detailed descriptions of the simulations are provided in **Supplementary Note 3**. The simulation results qualitatively capture the key experimental trends observed in WAXS and rheology measurements: the shear-rate-dependent transition from perpendicular to mixed and finally to parallel orientation relative to the flow direction, and the corresponding double shear-thinning behaviour. Although the NEMD simulations operate at spatial and temporal scales different from those of the experiments, they provide qualitative mechanistic insight into how the rheological behaviour of the smectic ink, particularly the double shear-thinning response, governs the shear-rate-dependent director switching observed during printing."*

2. The proposed orientation switching relies on adjusting either the print speed or the print temperature. While varying the print speed is practical, temperature control may limit the scalability or continuity of this method. Frequent temperature adjustments, particularly cooling, during printing could be time-consuming and may affect the homogeneity and interfacial bonding of printed filaments. In Figure 5, the printed structures appear to exhibit domain boundaries among regions printed at different speeds or temperatures. The authors should discuss how these transition regions affect the mechanical integrity and actuation uniformity of the printed samples. Do these boundary regions maintain uniform thickness? Furthermore, what is the smallest achievable size of independently controlled mesogen domains?

Response:

We thank the reviewer for this insightful comment. We agree that temperature modulation during printing may limit process continuity, as thermal equilibration, particularly cooling, is inherently time-consuming and could affect filament homogeneity and interfacial bonding. Print speed modulation (i.e., shear-rate modulation), by contrast, enables nearly instantaneous director switching without thermal lag, making it a more practical approach for continuous and scalable fabrication. To further highlight this practicality, we demonstrated speed-mediated orientation programming on shape-memory polymer (SMP) substrates (**Figs. R1 and R2**). For more details, please refer to our response to **Reviewer #1, Q2**.

As pointed out by the reviewer, domain boundaries are visible in **Figure 5** between regions printed at different speeds. These apparent boundaries may arise from (1) differences in filament overlap across print speeds and (2) optical contrast variations due to differences in light scattering between perpendicularly and parallelly aligned domains. The print speeds (3, 5, and 10 mm s^{-1} at 40 °C) produced filament widths of 0.78, 0.66, and 0.56 mm (**Supplementary Fig. 6**), with corresponding thicknesses of 0.36, 0.24, and 0.15 mm, respectively. Interface bonding between adjacent filaments was ensured by defining the infill densities based on the smallest filament width (0.56 mm at v_{nozzle} = 10 mm s^{-1}). This inevitably resulted in greater filament overlap in regions printed at 3 and 5 mm s^{-1}; however, infill densities ranging from 100% to 200% led to only marginal differences in actuation strain (~1.1%) as shown in **Figure R15**. Overall, local variations in film thickness and filament overlap at transition regions did not significantly compromise the mechanical integrity or actuation uniformity of the printed structures.

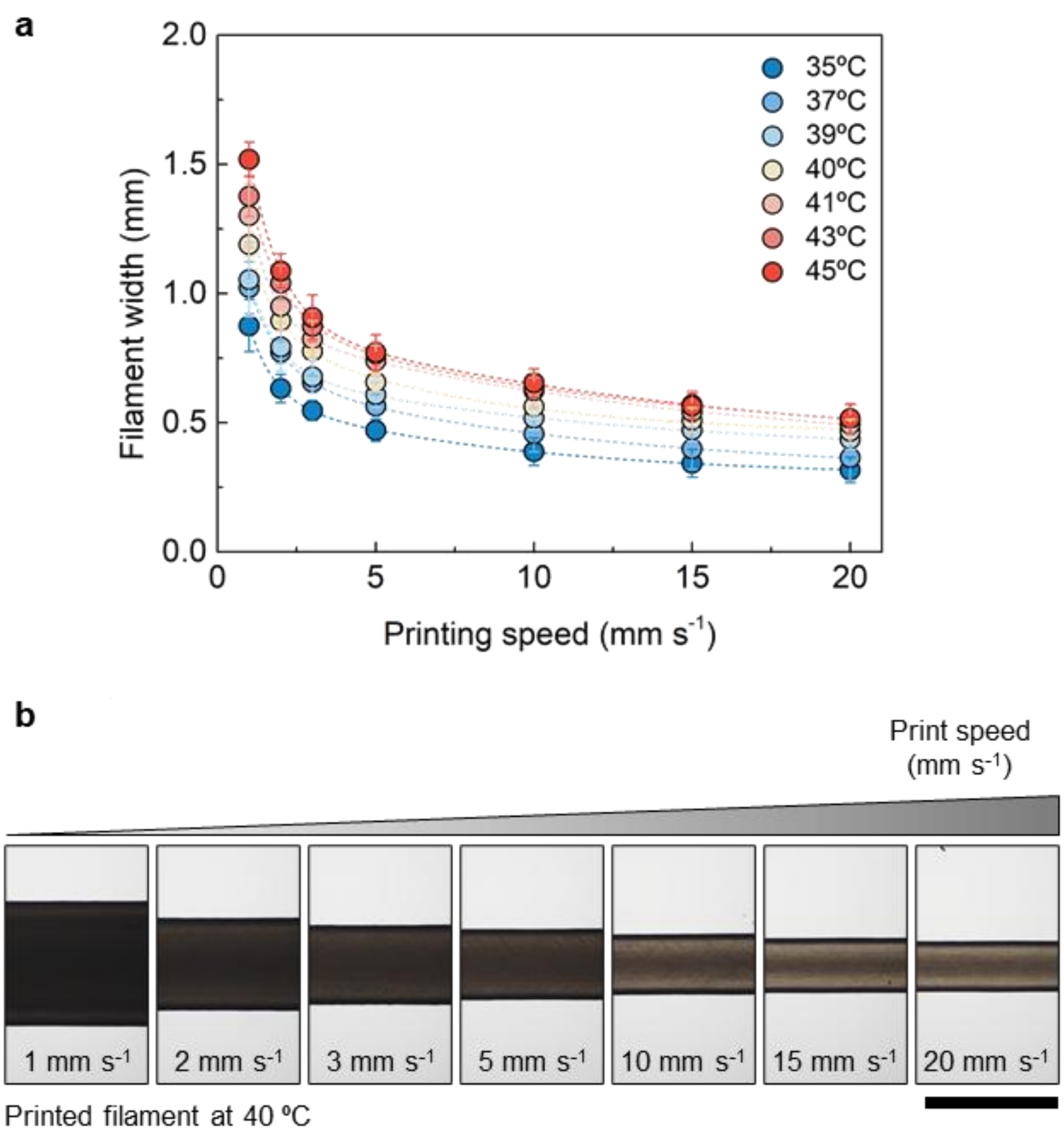

Supplementary Figure 6 | Printed filament width measured using optical microscopy. a, Filament width printed at various temperatures as a function of print speed. Solid markers represent the mean values, error bars indicate standard deviations ($N = 3$), and dashed lines show exponential decay fits to the mean values. **b,** Representative optical microscope images of filaments printed at 40 °C with varying print speeds. Scale bar = 500 μm.

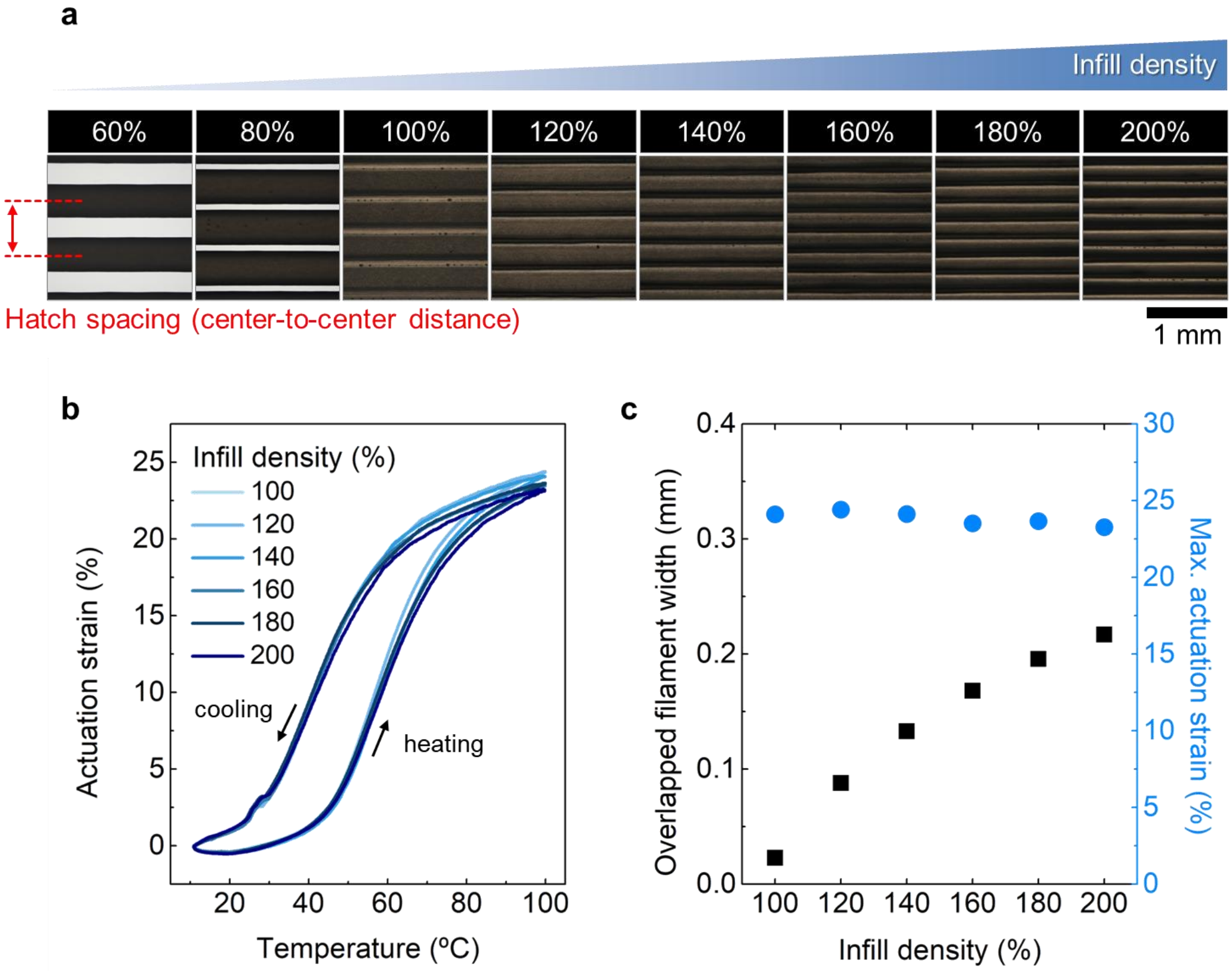

Figure R15. Effect of filament overlap on the thermal actuation of printed smectic LCE films. (a) Optical microscope images of LCE films printed at T_{nozzle} = 35 °C and v_{nozzle} = 5 mm s^{-1} under a pneumatic pressure of 500 kPa using a 0.4 mm-diameter nozzle, with varying infill density (defined as hatch spacing / nozzle diameter × 100%). The actual width of a single printed filament was measured to be ~416.4 μm and infill densities were controlled by adjusting the hatch spacing. (b) Reversible actuation strain of printed LCE films with different infill densities as a function of temperature. Each curve corresponds to the second heating–cooling cycle between 10 and 100 °C at a rate of 5 °C min^{-1}, measured by dynamic mechanical analysis. (c) Overlapped filament width (defined as actual filament width minus hatch spacing) and maximum actuation strain of printed LCE films as a function of infill density.

To address the reviewer's question regarding the smallest achievable domain size, we investigated the spatial resolution of print-speed-mediated director programming by alternating v_{nozzle} between 3 and 15 mm s^{-1} within a single continuous filament (**Fig. R4**). This produced periodic alternation between perpendicular and parallel director orientations, generating distinct local actuation modes along the filament upon thermal stimulation (**Fig. R1**). Independently controlled director

domains as short as 1 mm were reliably achieved using a 0.4 mm-diameter nozzle. The spatial resolution is primarily governed by nozzle diameter, and further miniaturization is expected to be achievable with smaller nozzle configurations.

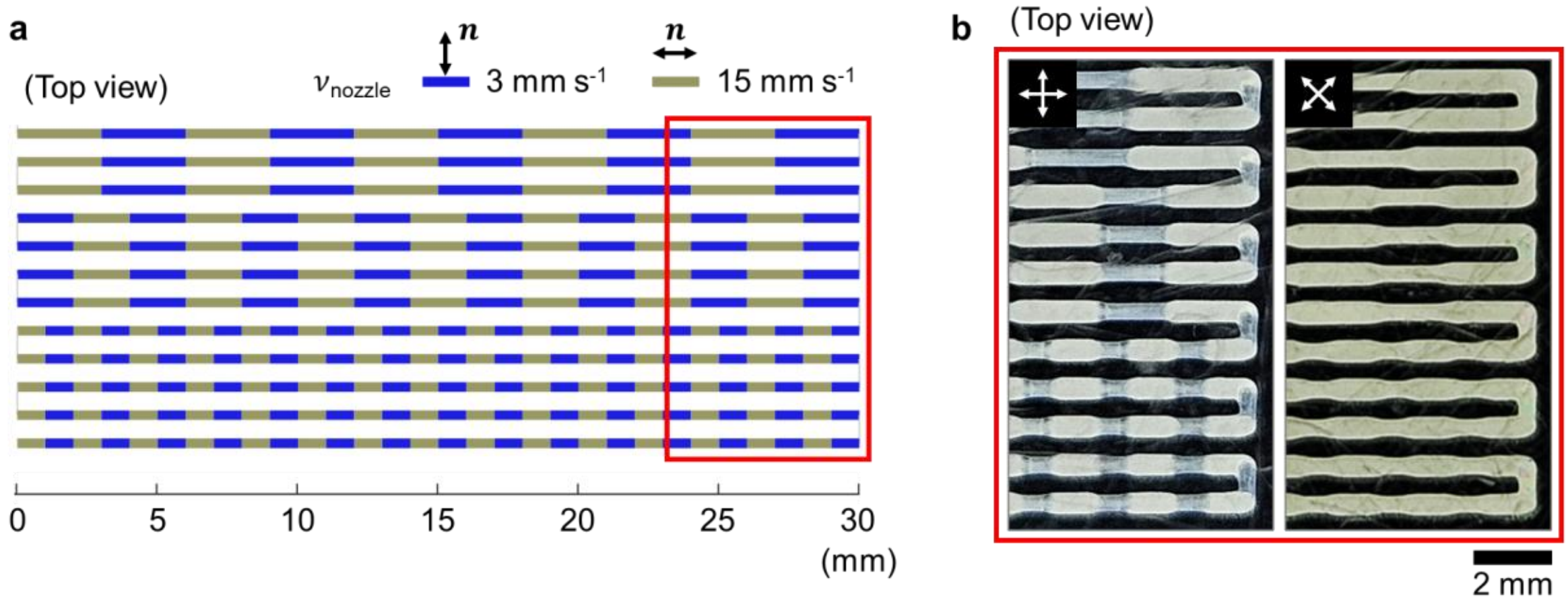

Figure R4. Smectic LCE filaments spatially programmed with alternating director patterns of different section lengths via encoded print speeds (v_{nozzle}). (a) Print path with encoded print speeds. Double arrows indicate the director ($\vec{n}$). (b) Polarized optical image of the printed filament acquired between crossed polarizers aligned (left) parallel to and (right) rotated by 45° with respect to the filament axis. Sections printed at v_{nozzle} = 3 mm s^{-1} and v_{nozzle} = 15 mm s^{-1} exhibit different birefringence intensities. White arrows indicate the orientations of the polarizer and analyzer.

3. In the finite element simulations, it is stated that the transverse thermal expansion coefficients are set to –αxx/2. What is the rationale behind this choice? Does this relationship ensure incompressibility of the material, where the product of stretch ratios along the x, y, and z directions equals 1?

In addition, how does the model ensure that each finite element continues to contract or elongate along its local filament direction during large deformation, given that the filament direction itself may rotate as deformation progresses? More explanation of these modeling assumptions and their physical implications would strengthen the analysis.

Response:

We thank the reviewer for pointing this out.

Regarding the assumption $\alpha_{yy} = -\frac{1}{2}\,\alpha_{xx}$ used in our numerical simulation, the dimensional response of LCEs upon temperature change is dominated by the liquid crystal–isotropic phase transition [Tajbakhsh & Terentjev, Eur. Phys. J. E 6, 181, 2001]. This transition-driven deformation proceeds at essentially constant volume due to the incompressibility of the rubber matrix [Warner & Terentjev, Liquid Crystal Elastomers, 2nd ed., Oxford University Press, 2007]. The near-incompressibility of the material under mechanical deformation is implemented by assigning a Poisson's ratio of 0.49. Similar assumptions have been adopted in previous studies of LCE thermal response [Wang et al., Sci. Adv. 6, eabc0034 (2020); Feng et al., Sci. Adv. 10, eadk3854 (2024)].

Regarding the material orientation, the filament direction in each finite element is defined via a user-specified local material orientation in ABAQUS/Standard [Abaqus Analysis User's Guide, Version 2023. Dassault Systèmes Simulia Corp., Providence, RI, USA (2023)]. Under large-displacement analysis, this local coordinate system rotates with the average rigid body motion of the material point, ensuring that the prescribed thermal expansion, whether contraction or elongation, is always applied along the current (deformed) filament direction rather than remaining fixed in the initial (reference) configuration.

This explanation has been added to **Supplementary Note 4** of the revised Supplementary materials.

Reviewer #3 (Remarks to the Author):

The manuscript reports a DIW strategy for smectic liquid crystal elastomers that enables switching between perpendicular and parallel molecular orientation within a single printed filament. This is an interesting contribution to the field of programmable soft materials. The idea of exploiting shear- and temperature-dependent alignment of smectic layers is novel and potentially impactful. However, the manuscript in its current form requires major revision before it can be considered for publication.

1. The manuscript would benefit from a clearer definition of the limitations in current DIW-based LCE alignment methods. In particular, outlining how existing single-mode strategies constrain programmability—and briefly comparing with previously reported multi-mode alignment approaches—would help better establish the research gap and highlight the contribution of this work.

Response:

We thank the reviewer for this thoughtful comment. In response, we have revised the Introduction to more clearly outline the limitations of existing DIW-based LCE alignment strategies and to better establish the research gap addressed by this work.

To date, DIW-based LCE fabrication has relied predominantly on flow-induced alignment of nematic mesogens, in which the director aligns uniformly along the printing direction regardless of processing conditions. While this approach is well-established, it is inherently limited to a single actuation mode, **namely contraction along the print direction,** within each printed filament. Recent studies have demonstrated that printing parameters such as print speed, temperature, nozzle geometry, and flow rate can be used to spatially modulate the degree of director alignment [Wang, Z. *et al. Sci. Adv*. **6**, eabc0034 (2020); Telles, R. *et al*. *Proc. Natl. Acad. Sci. U.S.A*. **122**, e2414960122 (2025); Ren, L. *et al*. *Addit. Manuf*. **61**, 103376 (2023)] or to generate through-thickness orientation gradients for bending actuation. [Zhang, C. *et al*. *ACS Appl. Mater. Interfaces* **11**, 44774–44782 (2019)] Nevertheless, in all these approaches, the director orientation within each filament remains fixed parallel to the print path, and achieving both contractile and elongational responses within a single printed filament has not been realized in DIW.

The present work addresses this limitation by exploiting the shear-rate-dependent director inversion of smectic LCs in a DIW platform. Unlike nematic systems, smectic LCEs exhibit a transition between perpendicular and parallel director orientations depending on the applied shear rate or printing temperature. In particular, shear rate control at constant temperature is advantageous because director orientation can be switched in real time through the nozzle translation speed. This enables spatially programmable director inversion within a single printed filament, providing a new degree of freedom for shape programming that is structurally inaccessible in nematic DIW.

To highlight the contribution of our work, we have revised the Introduction as follows:

Revised sentence (Introduction, Page 3, lines 58–89):

"*In particular, LCEs have emerged as promising candidates for extrusion-based direct-ink-writing (DIW) because their molecular orientation can be programmed during printing, enabling large and reversible anisotropic deformations. [23–26] LCEs are lightly crosslinked polymer networks containing rod-like mesogens. [27–31] The mesogen orientational order, described by the director ($\hat{n}$), couples to the polymer backbone, enabling reversible contraction along the director and expansion perpendicular to the director through order–disorder transitions. [32–39] In DIW, LC prepolymers are extruded through a nozzle and aligned by shear or extensional forces. [26,40] Subsequent in-situ crosslinking fixes the director orientation, enabling the fabrication of LCE structures with spatially programmed anisotropy. To date, DIW of LCEs has typically relied on nematic inks, in which mesogens exhibit only directional order and align uniformly along the print path regardless of processing conditions. [23–26,41,42] Thus, while this approach is well-established, it is inherently limited to a single actuation mode, namely contraction along the print direction, within each printed filament. Recent studies have demonstrated that printing parameters such as print speed, temperature, extrusion pressure, and nozzle geometry can be used to spatially modulate the degree of director alignment [25,26,43–45] or to generate through-thickness orientation gradients for bending actuation. [46] Nevertheless, in all these approaches, the director orientation within each filament remains fixed parallel to the print path, and achieving both contractile and elongational responses within a single printed filament has not been realized in DIW.*

In this work, we address this limitation by employing the switchable alignment behaviour of smectic LCs in DIW. Unlike nematic LCs, smectic LCs, which feature layered structures with directional order, offer two distinct alignment modes: (1) layer alignment in the smectic phase at low shear rates, producing a director orientation perpendicular to the print path, and (2) flow alignment of mesogens once the layers collapse at high temperatures or shear rates, yielding a parallel orientation (Fig. 1a).[47–51] By tuning printing parameters that govern the shear rate and temperature applied to the ink, smectic DIW enables switching of the director between perpendicular and parallel orientations within a single continuous print path. Given that main-chain LCEs contract parallel and expand perpendicular to the director, this switching provides access to both contractile and elongational shape changes within the same filament. Smectic DIW thus overcomes the limitation of existing DIW strategies, enabling diverse actuation programming within a single print path and complex director patterning within individual filaments or lattice structures that are inaccessible with nematic DIW (Figs. 1b–d)."

2. It would strengthen the manuscript to provide a more systematic analysis of the printing processing window. At present, only a speed–temperature map is shown, but key factors such as nozzle diameter, ink viscosity range, and shear-rate quantification are not discussed. In addition, the manuscript does not clarify whether the same alignment behavior holds at different printing heights or under varying filament overlaps, both of which may affect director orientation and the resulting actuation strain. A more comprehensive evaluation of printing robustness would improve the clarity and reproducibility of the proposed method.

Response:

We appreciate the reviewer's thorough and constructive comments. As suggested, we first quantified our printing processing window in terms of shear rate, calculated using the Couette flow approximation:

$$\dot{\gamma} = \frac{velocity\ difference\ between\ plates}{distance\ seperating\ plates} = \frac{v_{\text{nozzle}}}{H}$$

where v_{nozzle} is the nozzle translation speed and H is the gap distance between the nozzle tip and the substrate. The reconstructed heat map of maximum actuation strain (ϵ_a) versus shear rate ($\dot{\gamma}$), with $\dot{\gamma}$ on the bottom x-axis and v_{nozzle} on the top x-axis, is shown in **Fig. R16**. The validation of this approximation, confirmed by volumetric flow rate measurements and CFD simulations, is discussed in detail in our response to Reviewer #1, Q4.

Our results indicate that the applied shear rate ($\dot{\gamma}$) is the key processing parameter governing director orientation switching in smectic DIW. Specifically, the critical shear rate for orientation switching (from perpendicular to parallel director alignment) corresponds to the onset of the second shear-thinning regime in the steady-shear viscosity curve of the smectic ink, and is therefore an intrinsic material property independent of specific nozzle geometry or translation speed. To provide a more comprehensive evaluation of printing robustness, we conducted additional experiments addressing the effects of (1) ink molecular weight (viscosity), (2) nozzle diameter (d_{nozzle}), (3) printing height (H), and (4) filament overlap on the alignment and actuation properties of the printed smectic LCEs, as detailed below.

(1) Effect of ink viscosity on alignment and actuation behavior

To examine the effect of ink viscosity on director alignment, we compared two smectogenic inks with different molecular weights: (1) the original ink ($M_n \approx 3.8$ kDa, **Fig. R16a**) and (2) a higher molecular

weight ink ($M_n \approx 5.0$ kDa, Fig. **R16b**). Both inks show identical thermotropic behavior (T_i ~40 °C) as confirmed by GPC and DSC measurements (**Figs. R9 and R10a**). As shown in **Fig. R16b,** increasing M_n resulted in an approximately 4–5-fold increase in overall steady-shear viscosity, and the double shear-thinning behavior shifted to lower shear rates.

Accordingly, the onset of the second shear-thinning regime shifted from $\dot{\gamma} \approx 40–60\ s^{-1}$ (original ink) to $\dot{\gamma} \approx 10–20\ s^{-1}$ (higher M_n ink), and the critical shear rate for orientation switching near T_i, determined from the actuation strain heat map, shifted correspondingly from ~50 s^{-1} to ~10 s^{-1}. These results confirm that the onset of the second shear-thinning regime serves as a reliable indicator of the critical shear rate for alignment switching, and that the processing window for director inversion can be systematically tuned by adjusting ink molecular weight.

(2) Effect of nozzle diameter (d_{nozzle}) and (3) printing height (*H*) on alignment and actuation behavior

To decouple the individual contributions of d_{nozzle} and H to the applied shear rate, we systematically varied these parameters while maintaining $T_{nozzle} = 40$ °C and P = 500 kPa constant. **Figure R7** presents actuation strain heat maps as a function of v_{nozzle} and H for two nozzle diameters (0.4 mm and 0.6 mm). Regardless of d_{nozzle}, increasing H shifted the critical v_{nozzle} required for orientation switching to proportionally higher values, consistent with the Couette flow approximation $\dot{\gamma} = v_{nozzle} / H$. When the data are expressed in terms of $\dot{\gamma}$, the transition from perpendicular to parallel director alignment occurred within a consistent range of 33–67 s^{-1} across all tested combinations of d_{nozzle} and H. This range aligns well with the onset of the second shear-thinning regime in the steady-shear viscosity curve, further confirming that shear-induced director inversion is governed by the applied shear rate rather than by v_{nozzle} or H individually. These results demonstrate that our processing principle is robust across a range of nozzle geometries and printing heights, provided that the nozzle tip shear is effectively applied to the extruded ink.

(4) Effect of filament overlap on alignment and actuation behavior

To evaluate the effect of filament overlap on director alignment and actuation performance, we printed smectic LCE films with varying infill densities, defined as (hatch spacing / nozzle diameter) × 100%, and characterized their thermal actuation properties (**Fig. R15**). As infill density increases, the degree of overlap between adjacent filaments increases accordingly. Nevertheless, the maximum actuation strain of the resulting films remained nearly invariant across all tested infill densities, with a variation of only ~1.1%. These results indicate that the director alignment established during printing is robust against filament overlap under the conditions examined, and that infill density can be adjusted within the tested range without compromising actuation performance.

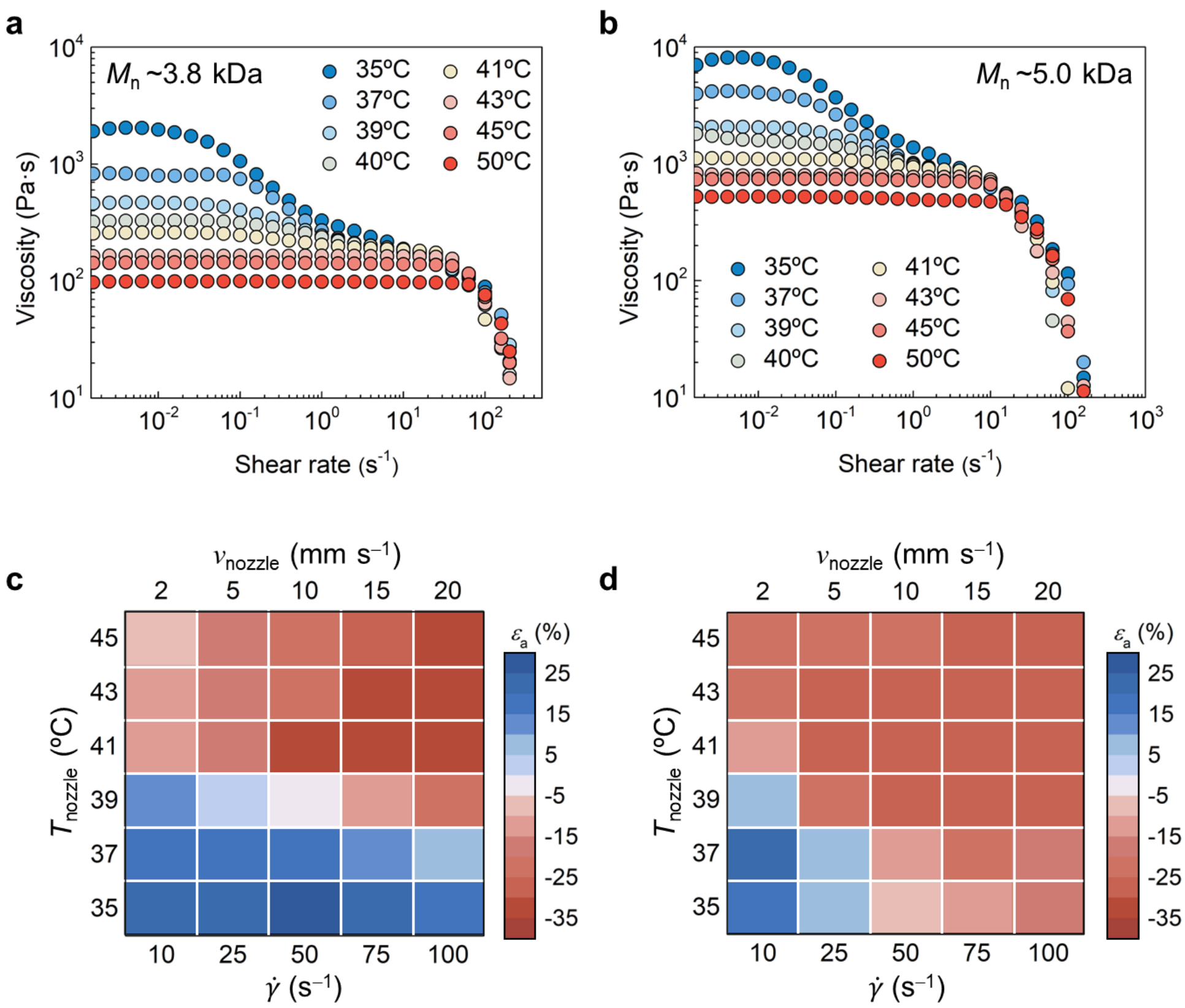

Figure R16. Effect of molecular weight on the rheological and actuation behaviors of 3D-printable smectic inks. Steady-shear viscosity as a function of shear rate at various temperatures for inks with (a) $M_n \approx 3.8$ kDa and (b) $M_n \approx 5.0$ kDa. Heat maps of actuation strain (ε_a) at 100 °C for filaments printed at varying nozzle translation speeds (v_{nozzle}) and temperatures (T_{nozzle}) under constant conditions of P = 500 kPa and d_{nozzle} = 0.4 mm for inks with (c) $M_n \approx 3.8$ kDa and (d) $M_n \approx 5.0$ kDa.

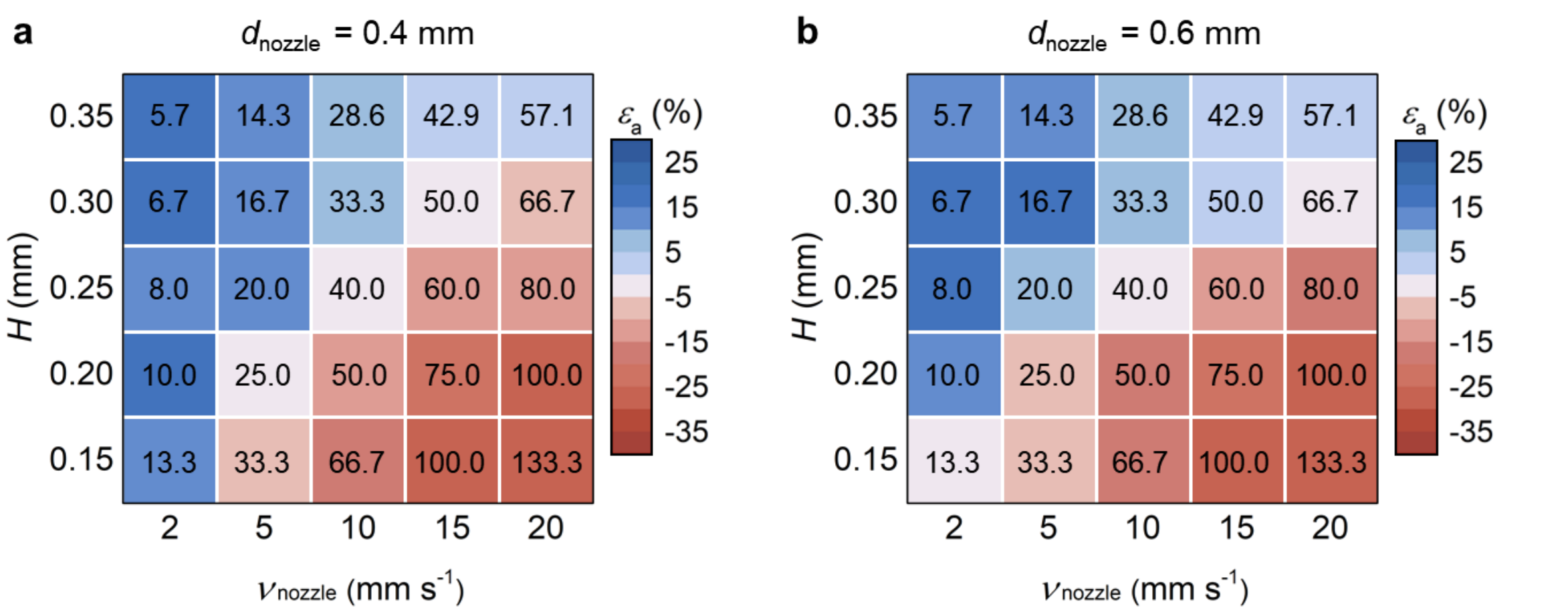

Figure R7. Effect of nozzle height (H) and nozzle diameter (d_{nozzle}) on alignment and actuation behavior. Heat maps of actuation strain (ε_a) at 100 °C for filaments printed at varying nozzle translation speeds (v_{nozzle}) and gap distances (H) under constant conditions of P = 500 kPa and T_{nozzle} = 40 °C, using 0.4 mm (a) and 0.6 mm (b) diameter nozzles. The values shown in each cell represent the applied shear rates ($\dot{\gamma} = v_{nozzle}/H$) at each condition.

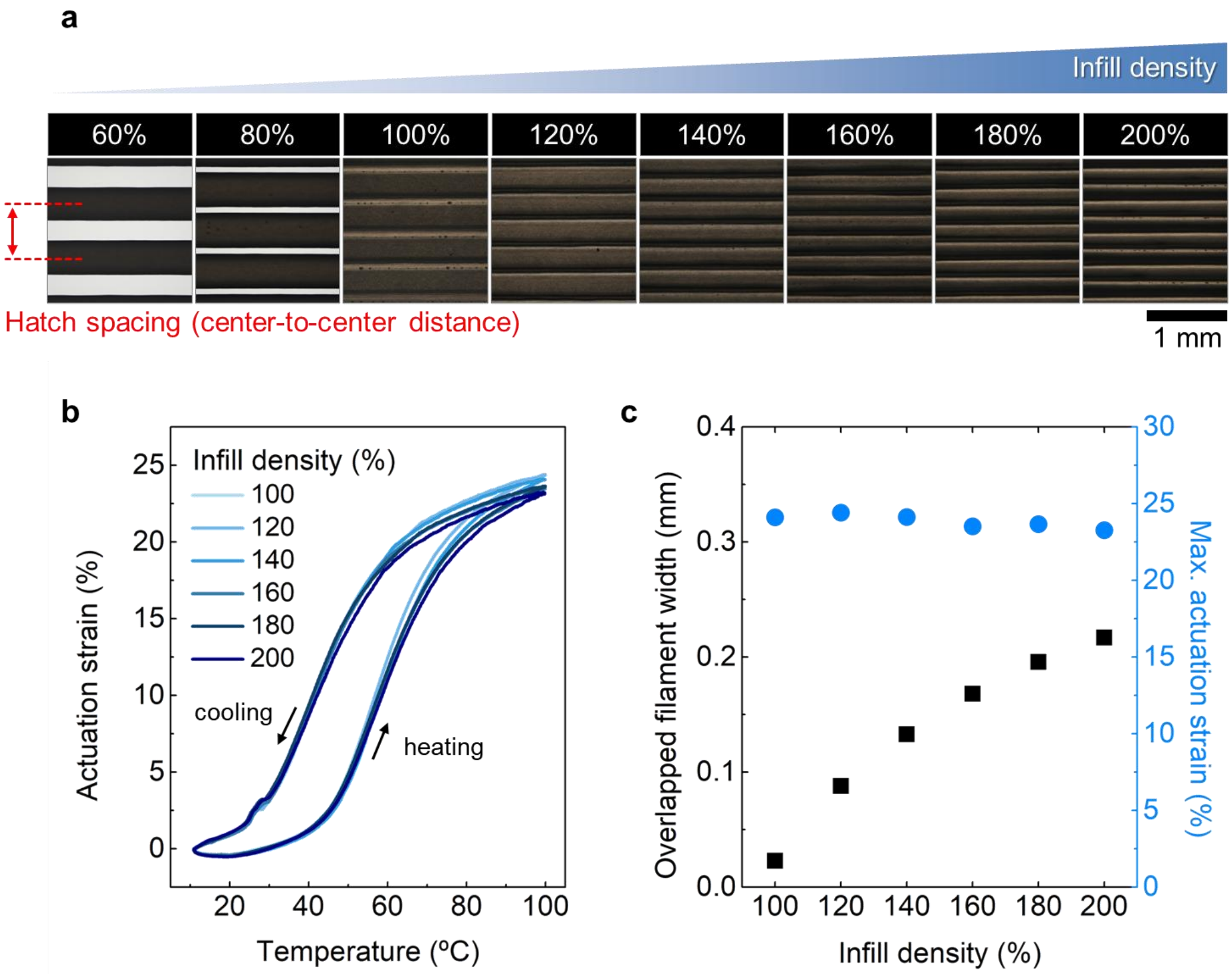

Figure R15. Effect of filament overlap on the thermal actuation of printed smectic LCE films. (a) Optical microscope images of LCE films printed at T_{nozzle} = 35 °C and v_{nozzle} = 5 mm s^{-1} under a pneumatic pressure of 500 kPa using a 0.4 mm-diameter nozzle, with varying infill density (defined as hatch spacing / nozzle diameter × 100%). The actual width of a single printed filament was measured to be ~416.4 μm and infill densities were controlled by adjusting the hatch spacing. (b) Reversible actuation strain of printed LCE films with different infill densities as a function of temperature. Each curve corresponds to the second heating–cooling cycle between 10 and 100 °C at a rate of 5 °C min^{-1}, measured by dynamic mechanical analysis. (c) Overlapped filament width (defined as actual filament width minus hatch spacing) and maximum actuation strain of printed LCE films as a function of infill density.

Taken together, these results demonstrate that the alignment behavior of smectic inks is fully governed by the applied shear rate and temperature, independent of specific nozzle geometry, printing height, or filament overlap. This shear-rate-based framework provides a generalizable and reproducible guideline for translating alignment behavior across different processing platforms and ink formulations.

3. While the manuscript claims "significantly expanded design freedom" and "diverse programmed shape changes," the demonstrations focus mainly on relatively simple 2D/3D structures. Including more complex or multi-step morphing examples—such as sequential folding, hierarchical deformation, or spatially coupled transformations—would better substantiate these statements and more convincingly showcase the advantages of the proposed approach.

Response:

We appreciate the reviewer's valuable comment and suggestion. We agree that more complex morphing demonstrations would better substantiate the claimed design freedom of the smectic DIW approach. To address this, we have added two new demonstrations in the revised manuscript.

First, we demonstrate spatial director programming within single filaments printed onto flexible SMP substrates (**Fig. R1**). By varying v_{nozzle} across different sections within a single filament, the resulting bilayer structures can be programmed to deform into either convex or concave geometries with tunable curvature ($\kappa \approx 0.1$–$0.3\ mm^{-1}$) (**Fig. R1b**), or to generate periodic undulations with tunable wavelength within a single continuous filament (**Fig. R1c**). These shape transformations arise from spatially distinct director orientations encoded within a single print path, a capability that is fundamentally inaccessible in DIW with nematic LCEs.

Second, we fabricated reversibly switchable surface topography actuators that demonstrate programmable, multi-region shape morphing (**Fig. R2**). Rectilinear print paths were encoded with different v_{nozzle} values section by section, producing complex surface topographies upon NIR irradiation, including (1) wavy stripes, (2) golf ball dimples, and (3) a human face pattern (**Fig. R2d**). These structures reversibly switch between near flat and topographic states (**Fig. R2e**), and by combining the shape-fixing capability of the SMP substrate with the reversible actuation of the LCE layer, the actuated topographies can be locked and unlocked on demand (**Fig. R2f–g** and **Supplementary Movie 8–9**). We consider these multi-region, programmable topographic transformations to represent the spatially coupled deformation the reviewer suggests, achieved here through a simple rectilinear print path without the need for complex alignment strategies.

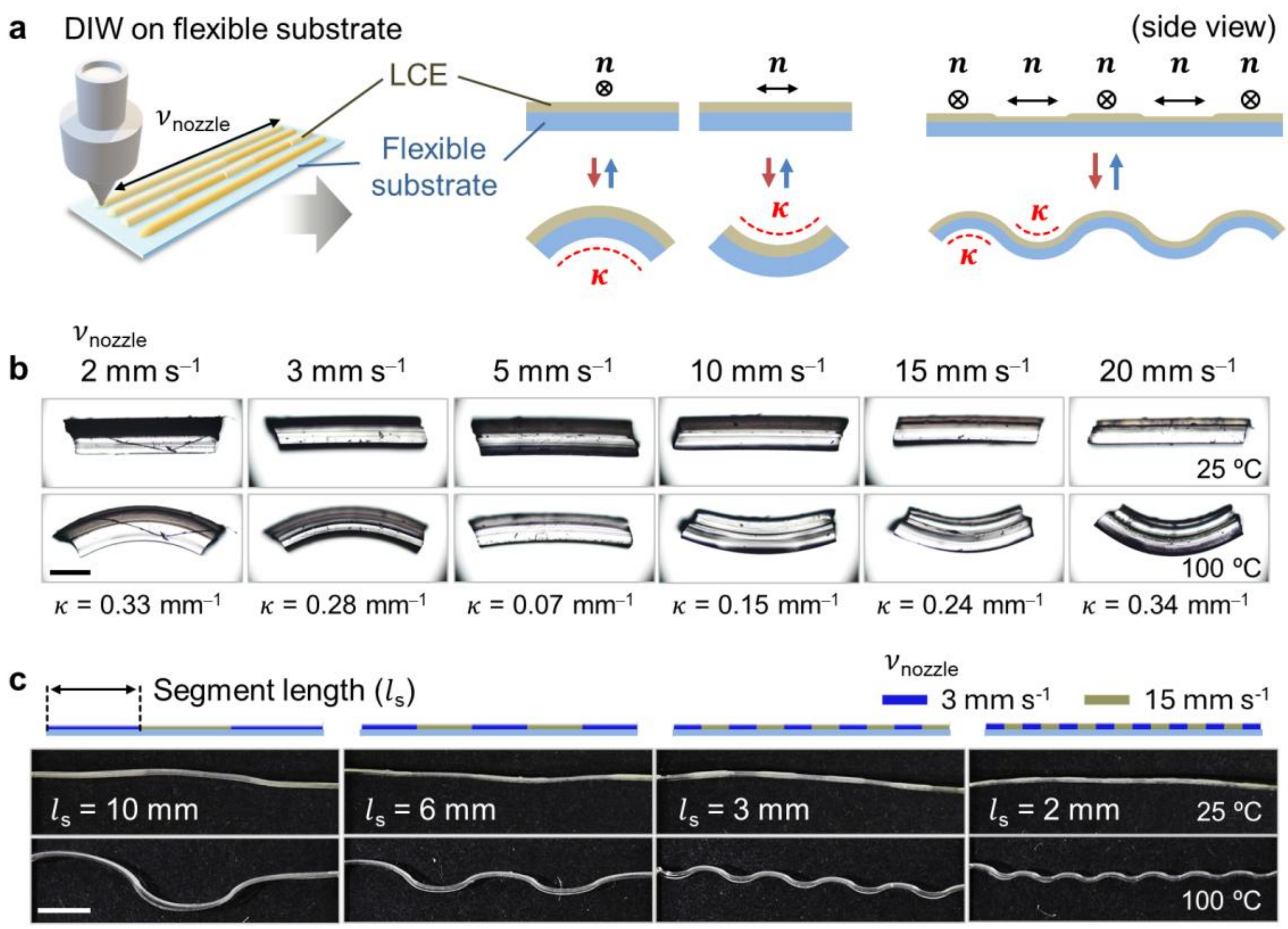

Figure R1. (a) Schematic illustration of shear rate controlled direct-ink-writing (DIW) of smectic liquid

crystal elastomers (LCEs) onto a shape memory polymer (SMP) substrate, poly(*tert*-butyl acrylate-*co*-*n*-butyl acrylate). Double arrows indicate the director ($\vec{n}$). (b) Optical microscope images showing side views of bilayer filament segments at 25 °C (unactuated) and 100 °C (actuated), printed at different nozzle translation speeds (v_{nozzle}). The curvature, κ (mm^{-1}), is defined as $1/R$, where R is the radius of curvature. Scale bar = 500 μm. (c) Optical images showing side views of bilayer filaments at 25 °C (unactuated) and 100 °C (actuated), printed with alternating v_{nozzle} of different periodicities. Scale bar = 5 mm.

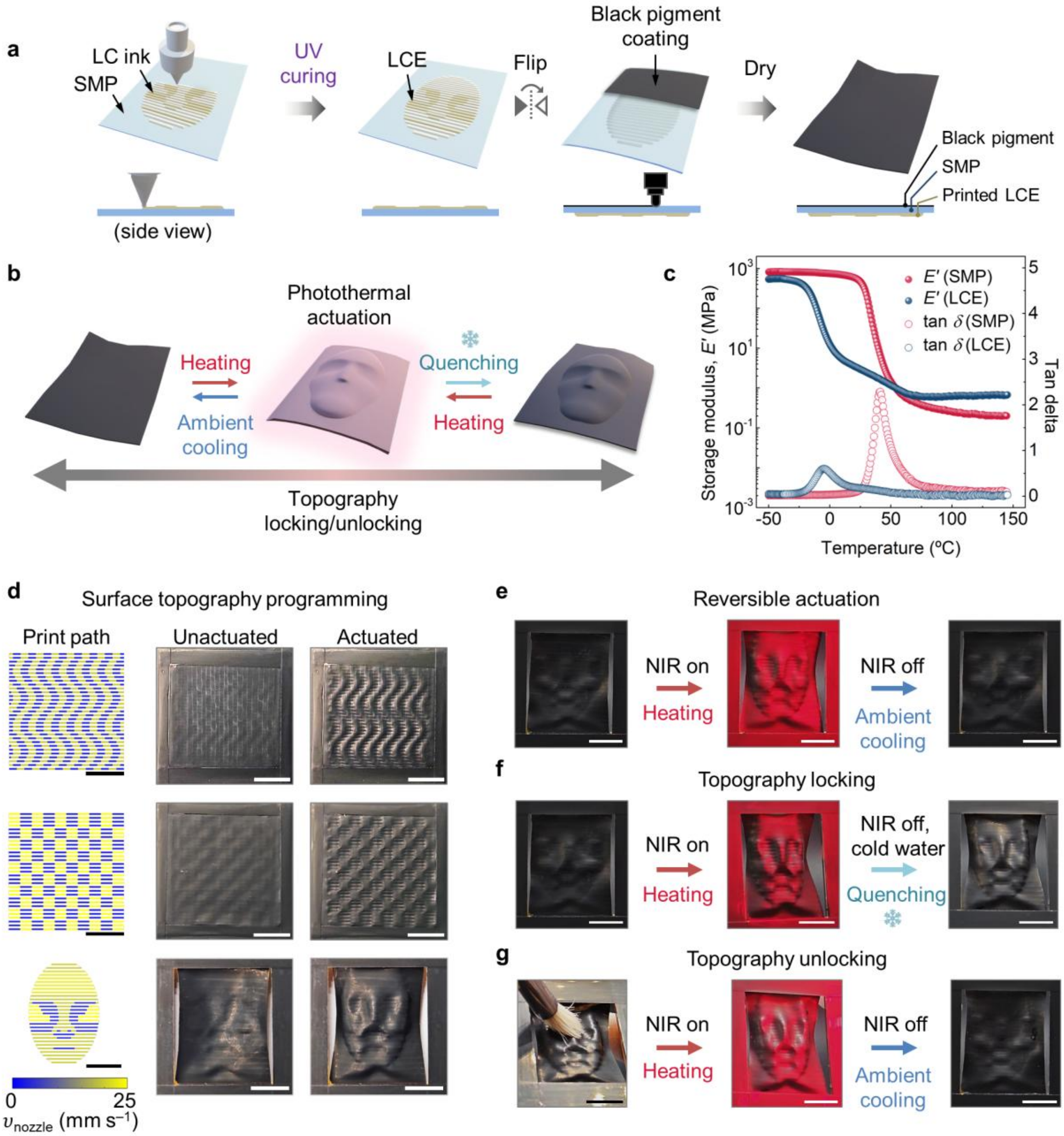

Figure R2. Reversibly switchable surface-topography actuator fabricated via shear rate controlled DIW of smectic LCEs onto SMP substrates. (a) Schematic illustration of fabrication process. Smectic LC filaments were printed onto SMP substrates at varying print speeds at 40 °C and subsequently UV-cured for 10 min. The opposite side of the printed surface was then coated with a black pigment to enable

photothermal actuation and dried for 10 min under ambient conditions. (b) Schematic illustration of reversible surface-topography switching of the actuator, arising from the actuation of the LCE layer and the shape-fixing capability of the SMP layer. (c) Viscoelastic properties of the smectic LCE and SMP measured by DMA. (d) Photographs of programmed surface-topography patterns, including wavy stripes (top), golf ball dimples (middle), and a human face (bottom), generated from rectilinear print paths with spatially encoded print speeds. Scale bars, 1 cm. (e–g) Snapshots of the transformable topography actuator programmed with a human-face pattern, showing reversible actuation (e), topography locking (f), and topography unlocking (g). Scale bars, 1 cm.

4. The manuscript would benefit from additional characterization of actuator performance. In particular, data on cyclic stability (fatigue behavior), response time, and mechanical output (e.g., generated force or stress) are not provided. These metrics are essential for evaluating the robustness and practical applicability of the printed LCE actuators, and including them would substantially strengthen the work.

Response:

We thank the reviewer for raising these important points. The cyclic stability of the printed smectic LCEs is discussed in detail in our response to Reviewer #1, Q6; please see our response therein. In short, the printed film was subjected to 100 thermal actuation cycles over a temperature range of 10–100 °C, and the actuation strain remained stable, showing only a marginal change of approximately 1.5% throughout all cycles, with no significant degradation in performance (**Fig.R12**).

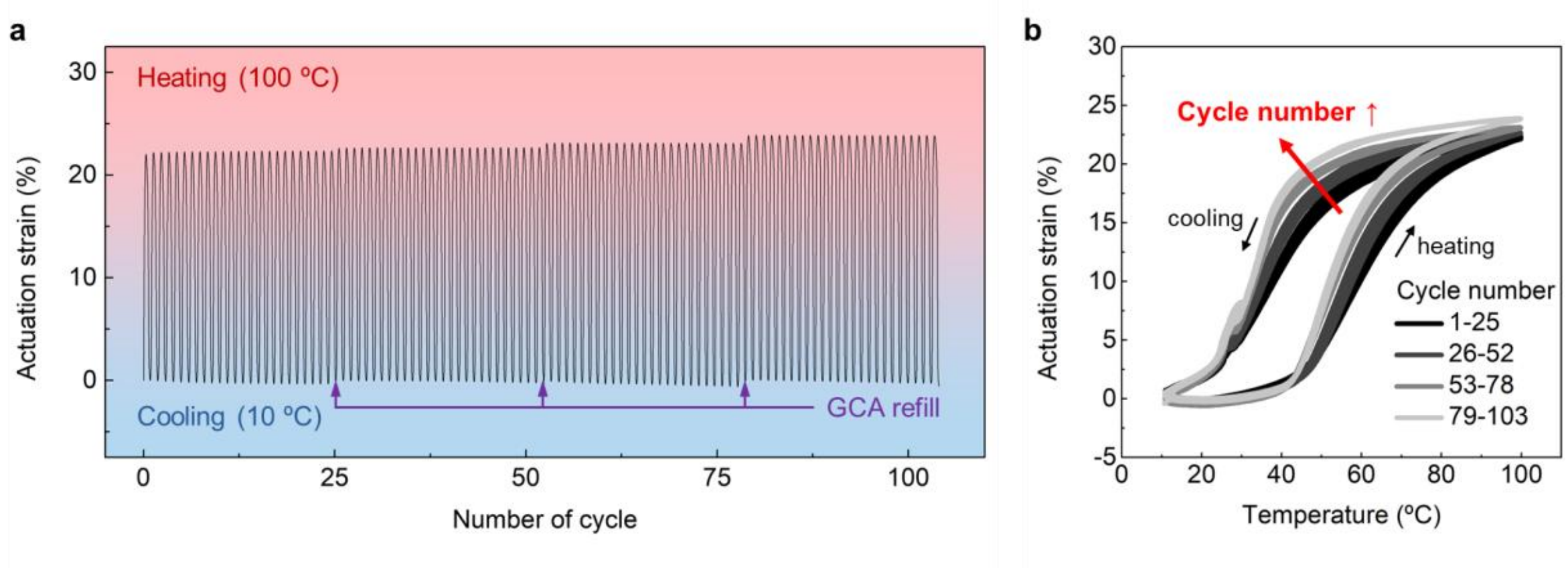

Figure R12. Actuation stability of the LCE film printed at T_{nozzle} = 35 °C and v_{nozzle} = 3 mm s^{-1}. (a) Thermal actuation strain measured over 103 heating–cooling cycles between 10 and 100 °C at a rate of 5 °C min^{-1} under a constant applied stress of 1 kPa. Approximately 25 cycles could be performed with a fully filled gas cooling accessory (GCA). The GCA was refilled with liquid nitrogen before the 26th, 53rd, and 78th cycles to enable testing over 100 heating–cooling cycles. (b) Thermal actuation strain as a function of temperature during the cyclic test. As the cycle number increased, the slope of the actuation curves became slightly steeper, while the maximum actuation strain changed only marginally, from 22.3% to 23.9% after 103 cycles.

We further evaluated the mechanical output of the printed smectic LCEs exhibiting elongational and contractile thermal actuation using isostrain tests on a dynamic mechanical analyzer

(**Fig. R17**). A compression-mode isostrain test was performed to measure the elongational actuation stress of an 8 (*w*) × 8 (*l*) × 8 (*t*) mm^3 cube (inset) printed at T_{nozzle} = 35 °C and v_{nozzle} = 10 mm s^{-1} (**Fig. R17a**). A cube geometry was adopted to prevent undesirable buckling during thermal elongation. In contrast, a tensile-mode isostrain test was conducted to measure the contractile actuation stress of a 5 (*w*) × 15 (*l*) × 0.2 (*t*) mm^3 film printed at T_{nozzle} = 45 °C and v_{nozzle} = 20 mm s^{-1} (**Fig R17b**). Blocking stresses of +0.30 MPa and −0.51 MPa were obtained for the elongational and contractile LCEs, respectively, where the positive and negative signs denote elongational and contractile actuation (**Fig R17c,d**).

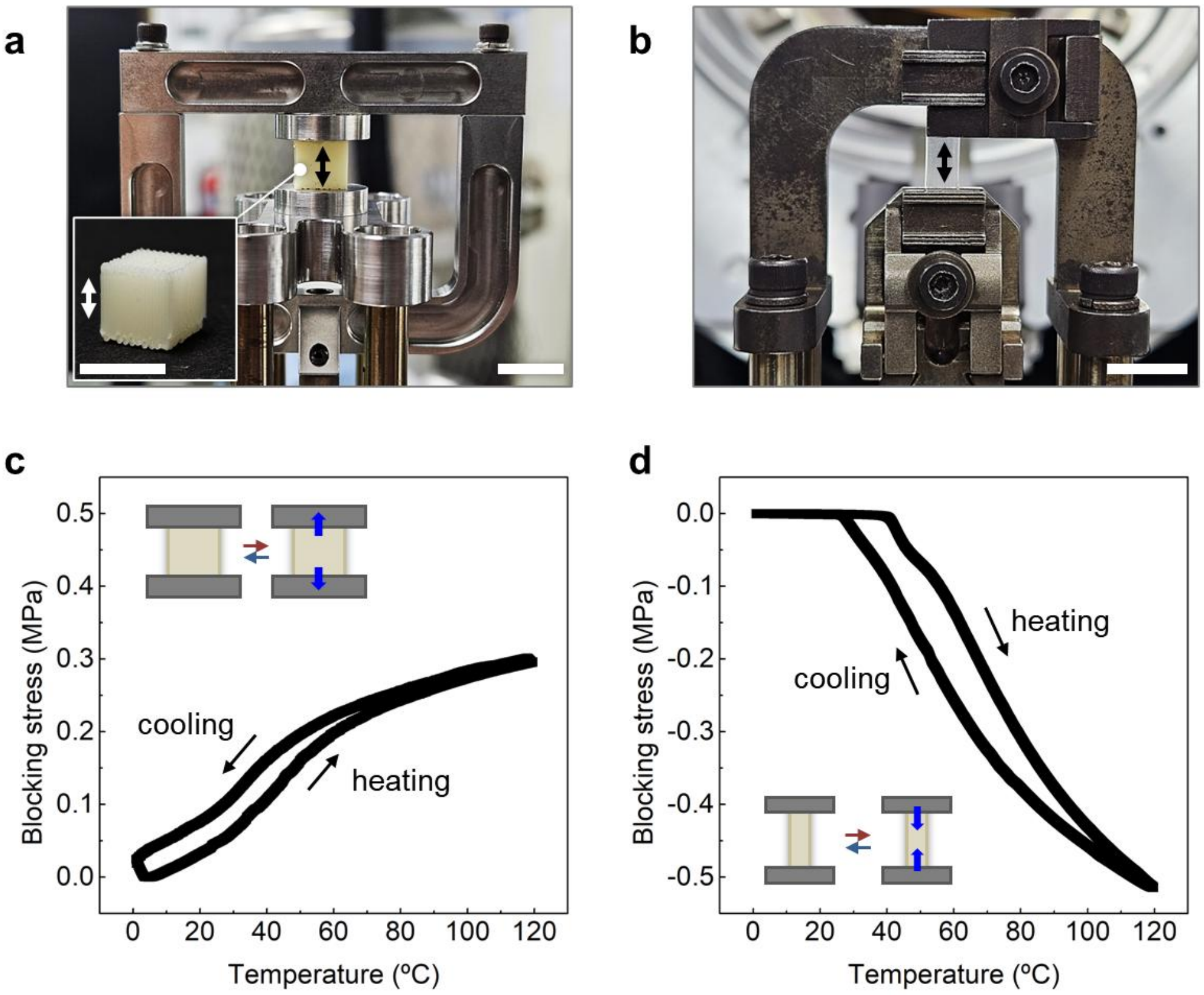

Figure 17. Isostrain test of printed LCEs. (a) Compression-mode dynamic mechanical analysis used to measure the elongational blocking stress of an 8 × 8 × 8 mm^3 cube (inset) printed at T_{nozzle} = 35 °C and v_{nozzle} = 10 mm s^{-1}. (b) Tensile-mode dynamic mechanical analysis used to measure the contractile blocking stress of a 15 × 5 × 0.2 mm^3 film printed at T_{nozzle} = 45 °C and v_{nozzle} = 20 mm s^{-1}. Bidirectional arrows indicate the print direction. Scale bars = 10 mm. (c, d) Blocking stress during elongational and contractile thermal actuation for the printed samples shown in (a) and (b), respectively, as a function of temperature. The blocking stress was measured under 0.1% isostrain during heating–cooling cycles between 0 and 120 °C at a rate of 3 °C min^{-1}. The second cycle is shown.

The actuation performances have been discussed in the revised manuscript on **Page 11, lines 299–307**, **Fig 4f–h**, and **Supplementary Figs. 22–23** of the Supplementary materials.

"The actuation performance of the printed smectic LCEs was evaluated using a dynamic mechanical analyser (Fig. 4f-g and Supplementary Fig. 22). Compression- and tensile-mode isostrain tests were conducted on representative elongating and contracting actuators printed at T_{nozzle} = 35 °C,

v_{nozzle} = 10 mm s^{-1} and T_{nozzle} = 45 °C, v_{nozzle} = 20 mm s^{-1}, revealing blocking stresses of +0.30 MPa and −0.51 MPa, respectively. The cyclic stability of the printed LCE was assessed under an isostress mode using a representative elongating LCE printed at T_{nozzle} = 35 °C and v_{nozzle} = 3 mm s^{-1} (Fig. 4h and Supplementary Fig. 23). The printed LCE was subjected to 100 thermal actuation cycles over 10–100 °C, during which the actuation strain remained stable, showing only a marginal change of approximately 1.5% and no significant performance degradation."

Response time was not the primary focus of this study, as it strongly depends on sample dimensions and the method of heat application, both of which influence thermal diffusion. Nevertheless, the reversibly switchable surface topography actuators shown in **Fig. R2** exhibited a response time of approximately 10–20 s for both actuation and recovery under NIR irradiation.

5. The manuscript would benefit from clearer reporting of the UV-curing conditions (intensity, exposure time, distance), as these parameters can influence director relaxation and final alignment.

Response: We thank the reviewer for this comment. We have revised the manuscript to provide a clearer description of the UV-curing conditions, as follows:

Original sentence:

~~"Single-layer structures were printed onto glass slides and crosslinked in a custom UV chamber equipped with a 400 W metal-halide lamp (CLEO HPA, iSOLde, λ = 300–400 nm) for 10 min."~~

Revised sentence:

"*Single-layer structures were printed onto glass slides and subsequently crosslinked in a custom UV chamber equipped with a 400 W metal-halide lamp (CLEO HPA 400/30 SD L, iSOLde, λ = 315–400 nm) for 10 min. The distance between the lamp and the samples was set to approximately 45 cm, at which the light intensity was measured to be 85 mW cm^{-2}.*" (**Page 16, lines 440–444**)

6. The origin of the "mixed alignment" state is mentioned but not fully clarified. More discussion or supportive data would help readers understand the transition regime between perpendicular and parallel alignment.

Response:

We thank the reviewer for this insightful comment. We have substantially revised the discussion of the mixed-orientation state in the manuscript, incorporating both additional experimental characterization and a revised physical interpretation.

As described in detail in our response to **Reviewer #1 (Q5)**, we performed GI-WAXS measurements on representative printed LCE films to spatially characterize the alignment distribution across the film thickness. Notably, for the mixed-orientation sample (T40V10), no significant difference in alignment direction was observed between the top and bottom surfaces, indicating that the mixed state does not arise from a through-thickness director gradient as originally speculated.

Based on these findings, we now propose a revised physical interpretation. Because the shear rate is effectively uniform across the film thickness and the relaxation time of the LC oligomer is considerably slower than that of low-molecular-weight liquid crystals, the observed mixed-orientation

pattern likely originates from a transient orientational state near the transition regime. That is, the slow relaxation dynamics of the oligomeric LC result in an intermediate, kinetically trapped orientation rather than a discrete perpendicular or parallel alignment.

Accordingly, we have revised the relevant discussion in the manuscript as follows:

Original sentence:

~~"The emergence of mixed patterns arises from differential alignment across the film thickness, where the director orientation near the phase transition becomes highly sensitive to subtle shear and temperature gradients."~~

Revised sentence:

"We speculate that the emergence of mixed patterns near the transition regime arises from the slow relaxation dynamics of the LC oligomer compared to low–molecular weight liquid crystals. In this regime, the orientation distribution appears as a coexistence of perpendicular and parallel alignment rather than a purely discrete orientation." (**Page 7, lines 159–162**)

7. Although NEMD simulations qualitatively reproduce the alignment transition, a more explicit quantitative comparison between simulated and experimental shear-rate thresholds would strengthen the mechanistic interpretation.

Response:

We thank the reviewer for this suggestion. As described in our response to **Reviewer 2, Q4**, we have presented additional comparisons between NEMD simulations, WAXS measurements (see **Supplementary Note 3** and **Supplementary Fig. 16**), and rheological experiments (see **Supplementary Note 3** and **Supplementary Fig. 17**), which together further strengthen the mechanistic interpretation of the alignment transition.

We note, however, that a strict quantitative comparison of absolute shear-rate thresholds between simulation and experiment is inherently limited by the coarse-grained nature of the model. In this model, mesogens are represented by simplified bead-spring chains that capture generic smectic behavior, rather than the specific molecular architecture and viscoelastic response of the experimental ink. Accordingly, the NEMD simulations are intended to provide mechanistic insight into the shear-induced evolution and collapse of smectic layering, namely, the molecular-level origin of director inversion, rather than quantitative prediction of absolute shear-rate thresholds.

Accordingly, we have included following sentences in the revised manuscript on **Page 9, lines 233–242**:

*"2D scattering functions in the flow–vorticity plane and viscosity plots as a function of shear rate are obtained from NEMD simulations (**Supplementary Figs. 16–17**). Detailed descriptions of the simulations are provided in **Supplementary Note 3**. The simulation results qualitatively capture the key experimental trends observed in WAXS and rheology measurements: the shear-rate-dependent transition from perpendicular to mixed and finally to parallel orientation relative to the flow direction, and the corresponding double shear-thinning behaviour. Although the NEMD simulations operate at spatial and temporal scales different from those of the experiments, they provide qualitative mechanistic insight into how the rheological behaviour of the smectic ink, particularly the double shear-thinning*

response, governs the shear-rate-dependent director switching observed during printing.”
