# Supplementary material for: Alignment switching in 3D-printed smectic liquid crystal elastomers
Source: Nat Commun. 2026 Jul 10;17:8530. doi: 10.1038/s41467-026-75368-z (PMC13483228; doi:10.1038/s41467-026-75368-z)
Supplement: Supplementary file 2 [file 41467_2026_75368_MOESM1_ESM.pdf]

Supplementary Information

Alignment Switching in 3D-Printed Smectic Liquid Crystal Elastomers

Jin-Hyeong Lee,[1] Kyeong Pyo Kim,[2] Lijie Ding,[3] Michael Li,[4,5] Min Chan Kim,[1] Kyu Hyun,[1] Ji Hoon Kim[2], Jan-Michael Y. Carrillo,[4,6]* Suk-kyun Ahn[1,7,8,9]*

[1]School of Chemical Engineering, Pusan National University, Busan, Republic of Korea

[2]School of Mechanical Engineering, Pusan National University, Busan, Republic of Korea

[3]Neutron Scattering Division, Oak Ridge National Laboratory, Oak Ridge, TN, USA

[4]Center for Nanophase Materials Sciences, Oak Ridge National Laboratory, Oak Ridge, TN, USA

[5]Department of Electrical and Computer Engineering, University of Maryland, College Park, MD, USA

[6]Computational Sciences and Engineering Division, Oak Ridge National Laboratory, Oak Ridge, TN, USA

[7]Department of Polymer Science and Engineering, Pusan National University, Busan, Republic of Korea

[8]Research Institute of Industrial Technology, Pusan National University, Busan, Republic of Korea

[9]Research Institute for Convergence of Biomedical Science and Technology, Pusan National University Yangsan Hospital, Yangsan, Republic of Korea

Email: skahn@pusan.ac.kr

Email: carrillojy@ornl.gov

Supplementary Information

The PDF file includes:

Supplementary Notes 1–4

Supplementary Figures 1–29

Supplementary Tables 1–2

References

Supplementary Note 1

^{1}H nuclear magnetic resonance (NMR) end-group analysis for determining the number-average molecular weight (M_n) of the smectogenic ink

The ^{1}H NMR spectrum of the smectogenic ink (Supplementary Fig. 2b) shows three peaks in the range of 5.85–6.45 ppm (b–d) corresponding to the six protons of the diacrylate end groups (CH_2=CHCOO–), with integration set to six for calibration of other peaks. The peak at 8.12 ppm (a) corresponds to the two aromatic protons of the oligomer, with an integration of 12.60. From these values, the degree of polymerisation (DP) was calculated as (12.60/2) – 1 = 5.30. The number-average molecular weight was then estimated as M_n = DP × M_n (repeating unit) + M_n (end groups) ≈ 5.30 × 611.77 + 538.60 ≈ 3800 Da.

Supplementary Note 2

Validation of the Couette flow approximation via volumetric flow rate measurements

To validate the assumption that director alignment is governed by Couette flow between the nozzle tip and the substrate rather than by extensional flow or stretching of the extrudate, we measured the volumetric flow rate (Q) under two conditions: free extrusion from the nozzle (Q_{nozzle}) and during active printing (Q_{print}) (Supplementary Fig. 5a–c).

At P = 500 kPa, Q_{print} was consistently lower than Q_{nozzle} across the entire range of v_{nozzle} = 2–20 mm s^{-1} (Supplementary Fig. 5c). Although Q_{print} approaches Q_{nozzle} at higher translation speeds, it remains below Q_{nozzle} throughout the entire range tested, confirming that the overfed regime is maintained under all printing conditions used in this study. This indicates that the volumetric supply from the nozzle exceeds the amount the substrate can carry away under the given printing conditions. As a result, we conclude that the dominant shear experienced by the smectic ink occurs in the gap between the nozzle tip and the substrate.

Accordingly, the shear rate applied to the printed filament was estimated using a simple Couette flow approximation:

$$\dot{\gamma} = \frac{velocity\ difference\ between\ plates}{distance\ seperating\ plates} = \frac{v_{nozzle}}{H} \quad (1)$$

where v_{nozzle} is the nozzle translation speed and H is the gap distance between the nozzle tip and the substrate.

Supplementary Note 3

Non-equilibrium molecular dynamics (NEMD) simulations

We performed non-equilibrium molecular dynamics (NEMD) simulations of smectic-A-forming mesogens composed of eight connected Lennard-Jones (LJ) beads, based on the model proposed by Milchev.[1] This model was chosen because the experiments were conducted near the smectic A-isotropic transition temperature, where entropy is the dominant driver for the transition from smectic-A to nematic during 3D printing at shear rates above a critical value, and enthalpic interactions play a minimal role. This is consistent with Milchev's model, which considers only the repulsive part of the LJ potential, best described by the Weeks-Chandler-Andersen (WCA) potential[2] — a shifted and truncated LJ potential with a cutoff of $2^{1/6}$ σ.

The bonds between beads were maintained using finitely extensible nonlinear elastic (FENE) bonds,[3] and the mesogens were kept rigid and rod-like by imposing a bending potential between two neighbouring bonds. The bending potential energy,

$$U_{bend} = K_{bend}(1 - \hat{\mathbf{n}}_i \cdot \hat{\mathbf{n}}_{i+1}), \tag{2}$$

was set to $K_{bend} = 96\ k_B T$ in reduced LJ units, where $\hat{\mathbf{n}}_i$ is the i-th unit bond vector. The simulation box is composed of 12,696 mesogens (101,568 LJ beads) at a number density of $\rho = 0.8125\sigma^{-3}$. As in Milchev's work, the temperature was fixed at $T = 0.5$; however, in this study, a dissipative particle dynamics (DPD) thermostat was used instead of the Langevin thermostat employed by Milchev. The DPD thermostat implemented in LAMMPS enables NEMD simulations under simple shear deformation,[4] consistent with previous studies.[5,6]

To allow the system to tilt, we employed a triclinic simulation box. Independent Nosé-Hoover barostats were applied to P_{xy}, P_{xz}, and P_{yz}, maintaining these pressure components at zero. This allowed the smectic layers to orient correctly by minimising the cross-components of the pressure tensor. Prior to the NEMD runs, equilibrium molecular dynamics (MD) simulations were performed to confirm the formation of the smectic-A phase. These simulations were run for up to $40{,}000\,\tau$ with an integration timestep $\Delta t = 0.002\ \tau$, where τ is the LJ reduced time unit. Structural characterisation was carried out by computing the smectic translational order parameter (τ_1),[7,8] the orientational order parameter (S) and the tilt angle (θ) of smectic layers. The inputs for characterising the smectic layers were the set of mesogen centre-of-mass vectors $\mathbf{r}_i$ and orientational unit vectors $\hat{\mathbf{u}}_i$ within the simulation box. The orientational order parameter was obtained from the Q-tensor,

$$\mathbf{Q} = \frac{1}{N}\sum_{i=1}^{N}\left(\frac{3}{2}\,\hat{\mathbf{u}}_i\hat{\mathbf{u}}_i^T - \frac{1}{2}\,\mathbf{I}\right) \tag{3}$$

where the S is the largest eigenvalue of the eigenpair $\mathbf{Q}\hat{\mathbf{n}} = S\hat{\mathbf{n}}$.

The smectic translational order parameter was obtained via reciprocal-space analysis of the mesogen distribution. By performing a fast Fourier Transform (FFT) of the set of $\mathbf{r}_i$ vectors,

$$\tilde{\rho}(\mathbf{k}) = \sum_{i=1}^{N} e^{-i\,\mathbf{k}\cdot\mathbf{r}_i}. \tag{4}$$

where $\mathbf{k}_{m_x m_y m_z} = \left(\frac{2\pi m_x}{L_x}, \frac{2\pi m_y}{L_y}, \frac{2\pi m_z}{L_z}\right)$, the layer normal vector was identified as

$$\hat{\mathbf{k}} = \frac{\mathbf{k}^*}{||\mathbf{k}^*||}, \text{ where } \mathbf{k}^\star = \arg\max_{\mathbf{k}\neq 0} |\tilde{\rho}(\mathbf{k})|. \tag{5}$$

The translational order along $z_i = \mathbf{r}_i \cdot \hat{\mathbf{k}}$ was then computed as

$$\tau_1(q) = \left|\frac{1}{N}\sum_{i=1}^{N} e^{iqz_i}\right|, \tag{6}$$

where $q_0 = \arg\max_q[\tau_1(q)]$, $\tau_1 \equiv \tau_1(q_0)$ and the characteristic layer spacing is $d = \frac{2\pi}{q_0}$. The tilt angle between the director and the layer normal is

$$\theta = \arccos\left(\left|\hat{\mathbf{n}} \cdot \hat{\mathbf{k}}\right|\right). \tag{7}$$

All calculations were performed after remapping the triclinic box into an orthogonal representation.

Subsequently, NEMD simulations were conducted by applying simple shear deformation to the simulation box at variable shear rates ($\dot{\gamma}$), with the flow, velocity gradient, and vorticity oriented along the $\hat{\mathbf{x}}$, $\hat{\mathbf{z}}$, and $\hat{\mathbf{y}}$ directions, respectively. Structural characterisation was carried out on the trajectories, and results are reported once the system reached steady state or after $4000\ \tau$.

In the experiments, wide-angle X-ray scattering (WAXS) and rheology measurements report ensemble-averaged properties, implying that several smectic-A domains may be averaged. During the 3D printing at low shear rates, the deposition surface biases the orientation of both the smectic layers and the director ($\hat{\mathbf{n}}$) to be perpendicular to the surface normal and the flow direction ($\hat{\mathbf{x}}$). To account for this, we performed ~100 independent simulations starting from various initial layer orientations, described by the second Legendre polynomial,

$$P_2(\cos(\psi)) = \frac{1}{2}(3\cos^2(\psi) - 1), \tag{8}$$

where ψ is the angle between the director and the flow direction, defined as $\cos(\psi) = \hat{\mathbf{n}} \cdot \hat{\mathbf{x}}$. The simulations were initialised with different values of $P_2(cos(\psi))$, biased toward $P_2(cos(\psi)) \approx -0.5$ (see Supplementary Figure 13).

<u>NEMD-derived 2D scattering analysis for comparison with WAXS experiment</u>

If we consider only the mesogen end-groups as scatterers, the scattering function detects the in-plane structures as beads arranged within a plane. To obtain these scattering functions, we averaged over 100 independent simulations. The 3D scattering function is computed from the Fourier transform of the end-

group density:

$$S(\mathbf{q}) = S(q_x, q_y, q_z) = \frac{1}{N}\left\langle \sum_{j=1}^{N} \sum_{k=1}^{N} exp\left(i\, \mathbf{q} \cdot (\mathbf{r}_j - \mathbf{r}_k)\right)\right\rangle, \quad (9)$$

where $\mathbf{q} = (q_x, q_y, q_z)$, $\mathbf{r}_j$ is the position of the j-th mesogen end-group, and $\langle \cdots \rangle$ denotes an ensemble average over the 100 simulation snapshots.

To compare with the WAXS geometry, in which the beam is along $\hat{\mathbf{z}}$ and the detector records the in-plane q_x–q_y components, the 3D scattering intensity is projected onto the detector plane by evaluating:

$$S(q_x, q_y) = S(q_x, q_y, q_z = 0), \quad (10)$$

i.e., the slice of reciprocal space perpendicular to the beam direction. This yields the 2D scattering function in the flow–vorticity plane.

The sequence of scattering functions in the (q_x, q_y) plane in Supplementary Figure 16 shows the evolution of mesogen ordering under increasing shear rate in the NEMD simulations. At the lowest shear rate (leftmost panel), the pattern displays a nearly isotropic ring of intensity with weak maxima at $(0, \pm q^*)$, characteristic of a perpendicular smectic orientation, where the layer normal lies along the vorticity direction. As the shear rate increases, these perpendicular-orientation maxima grow in intensity and become more distinct (second panel).

With further shear (third and fourth panels), the system enters a coexistence regime: the original peaks at $(0, \pm q^*)$, associated with the perpendicular smectic state; persist, while new intensity maxima emerge at $(\pm q^*, 0)$, indicating the onset of a parallel orientation in which the smectic layer normal aligns with the flow direction. The coexistence of both sets of peaks reflects a shear-driven reorientation transition between perpendicular and parallel smectic organizations.

At the highest shear rate (rightmost panel), the circular ring associated with smectic layering disappears entirely, signaling the loss of positional layer order. The scattering collapses into two strong, elongated vertical lobes centered at $(\pm q^*, 0)$, producing a pattern characteristic of a nematic state with strong mesogen alignment along the flow direction and no remaining smectic periodicity.

<u>NEMD-derived shear-dependent viscosity for comparison with steady-shear experiment</u>

Supplementary Figure 17 shows the shear-dependent viscosity $\eta(\dot{\gamma})$ obtained from NEMD simulations under steady simple shear. For each shear rate $\dot{\gamma}$, the viscosity is computed from the

averaged shear stress,

$$\eta(\dot{\gamma}) = \frac{\langle \tau_{xz} \rangle}{\dot{\gamma}}, \tag{11}$$

where $\langle \tau_{xz} \rangle$ is the time- and ensemble-averaged xz component of the stress tensor, averaged over 100 independent simulations. The resulting viscosity curve exhibits two shear-thinning regimes, similar to the rheology experiments, each corresponding to a distinct structural transition observed in the scattering patterns.

At low shear rates, the system displays a perpendicular smectic orientation, resulting in relatively high viscosity. As the shear rate increases, the viscosity decreases smoothly, marking the first shear-thinning regime, coinciding with the transition from perpendicular orientation to a coexistence of perpendicular and parallel smectic domains. At higher shear rates, a second shear-thinning regime emerges, associated with the system adopting a predominantly parallel orientation and ultimately a nematic state with fully flow-aligned mesogens. This structural reorganization reduces resistance to flow, producing the characteristic double-sloped shear-thinning behavior shown in the Figure 2b.

Supplementary Note 4

Finite element simulations of printed LCEs

Regarding the assumption $\alpha_{yy} = -½\ \alpha_{xx}$ used in our numerical simulation, the dimensional response of LCEs upon temperature change is dominated by the liquid crystal–isotropic phase transition.[9] This transition-driven deformation proceeds at essentially constant volume due to the incompressibility of the rubber matrix.[10] The near-incompressibility of the material under mechanical deformation is implemented by assigning a Poisson's ratio of 0.49. Similar assumptions have been adopted in previous studies of LCE thermal response.[11,12]

Regarding the material orientation, the filament direction in each finite element is defined via a user-specified local material orientation in ABAQUS/Standard [Abaqus Analysis User's Guide, Version 2023. Dassault Systèmes Simulia Corp., Providence, RI, USA (2023)]. Under large-displacement analysis, this local coordinate system rotates with the average rigid body motion of the material point, ensuring that the prescribed thermal expansion, whether contraction or elongation, is always applied along the current (deformed) filament direction rather than remaining fixed in the initial (reference) configuration.

To simulate the spontaneous thermal deformation of LCEs, the non-linear thermal expansion coefficient (CTE) of the materials was determined using the ABAQUS subroutine UEXPAN.

Experimental data on dimensional changes along the axis parallel to the print direction of LCEs printed at different speeds (3, 5, 10 mm s^{-1}) were obtained via dynamic mechanical analysis (DMA) (Fig. 4a) The deformed lengths $L(T)$ as a function of temperature were fitted using the following empirical equation, which describes the material's thermal expansion behaviour:

$$L(T) = L_1 + \frac{1}{2}L_2(\tanh(L_3(T - L_4)) + 1) \quad (12)$$

where L_1, L_2, L_3, and L_4 are constants determined through fitting (Supplementary Table 2). The true strain $\varepsilon(T)$ was calculated using:

$$\varepsilon(T) = \ln\left(\frac{L(T)}{L(T_0)}\right) \quad (13)$$

where $L(T_0)$ is the initial length at the baseline temperature T_0= 20 ºC. The CTE along the print direction, α_{xx}, was then determined from the difference in strain $\varepsilon(T)$ at the elevated temperature T and the reference strain ε_0, divided by the corresponding temperature difference:

$$\alpha_{xx} = \frac{\varepsilon(T) - \varepsilon_0}{T - T_0} \quad (14)$$

where $\varepsilon_0 = \varepsilon(T_0)$.

Thermal simulations of LCE discs printed along concentric paths using these α_{xx} values showed good agreement with experimental observations. (Supplementary Fig. 27)

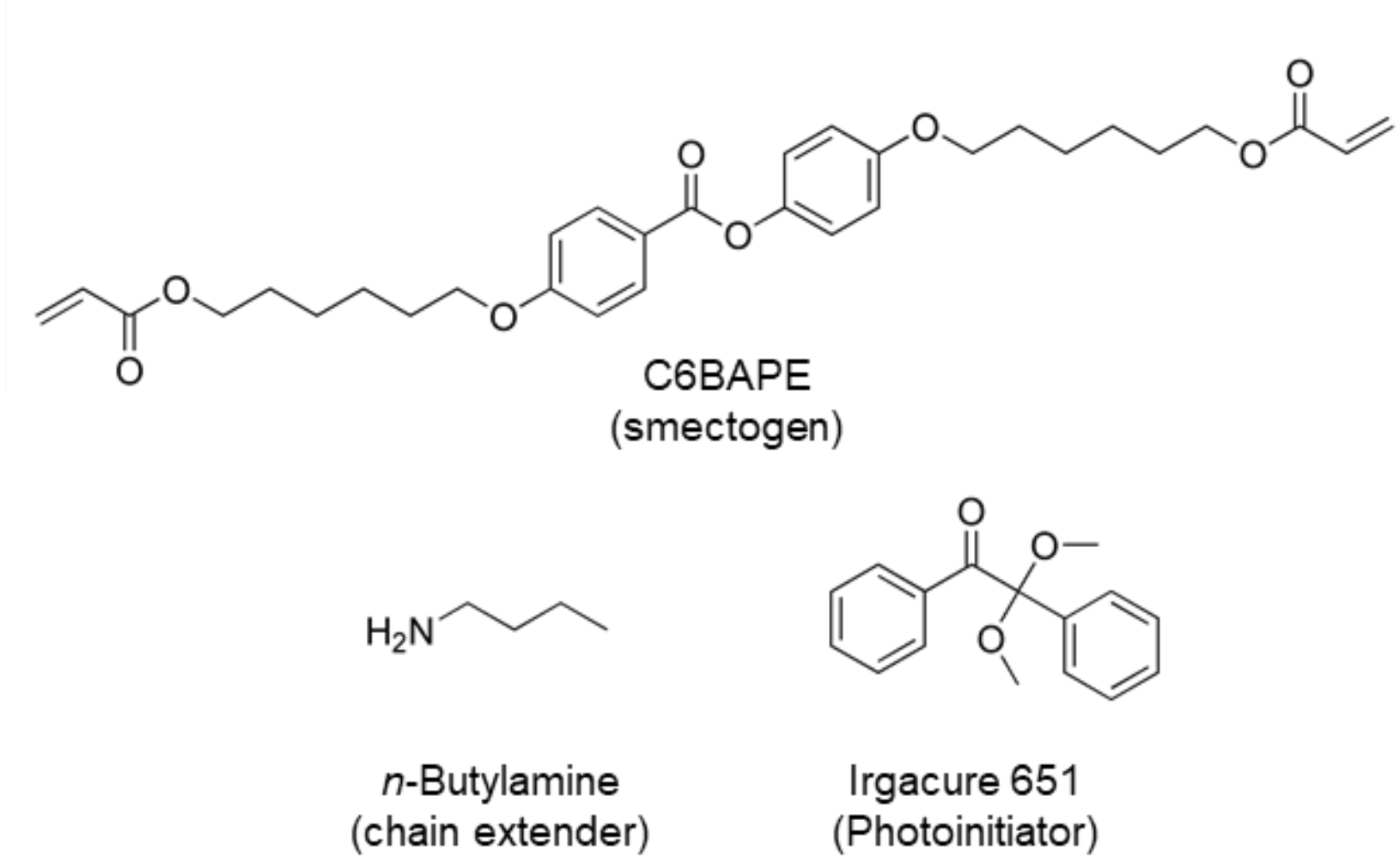

Supplementary Figure 1 | Molecular structures of the chemical components. C6BAPE (smectogen), *n*-butylamine (chain extender), and Irgacure 651 (photoinitiator) used to synthesise the 3D printable smectogenic ink.

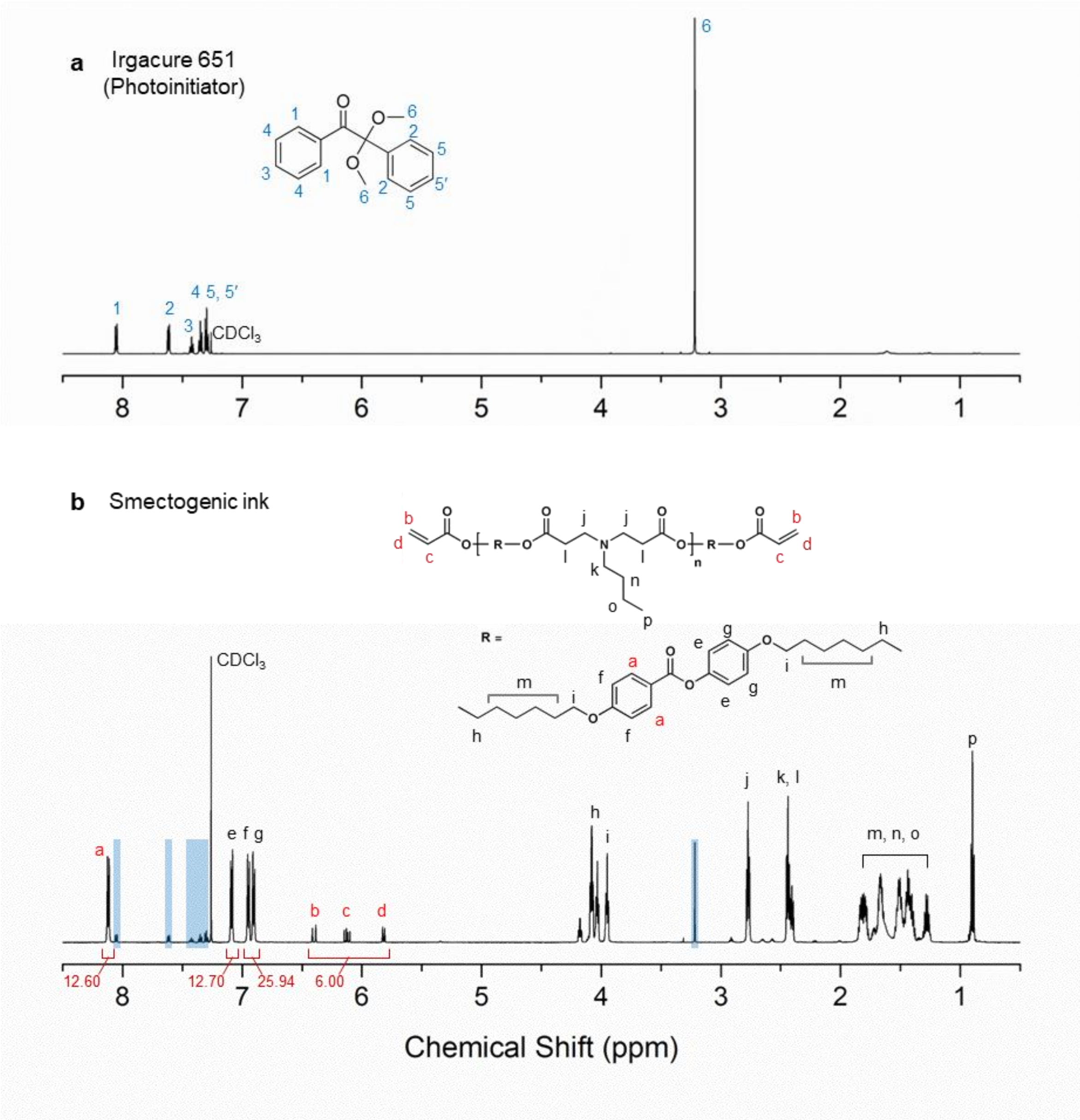

Supplementary Figure 2 | ^{1}H NMR spectra of photoinitiator and smectogenic ink recorded at 600 MHz in $CDCl_3$. a, ^{1}H NMR spectra of the photoinitiator (Irgacure 651). **b,** ^{1}H NMR spectra of the smectogenic ink. The light blue boxes highlight the peaks related to the photoinitiator in the ink. The three peaks between 5.85 and 6.45 ppm (b, c, d) correspond to the six protons in the diacrylate end groups of the oligomer (CH2=CHCOO–). The integration value of these peaks, set to six, was used to calibrate the other peaks.

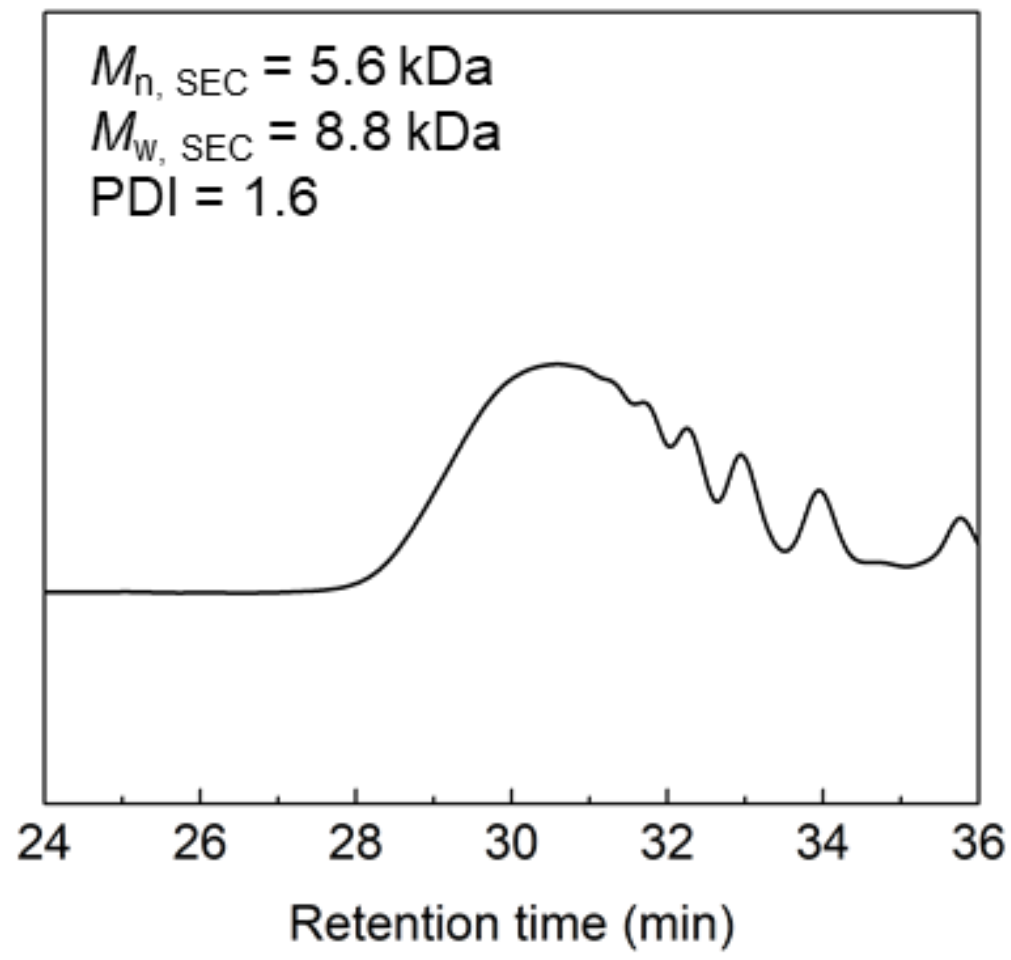

Supplementary Figure 3 | Size exclusion chromatography (SEC) trace of smectogenic ink. SEC trace recorded at a flow rate of 0.5 mL min^{-1} using tetrahydrofuran (THF) as the eluent. The calibration curve was constructed using polystyrene standards.

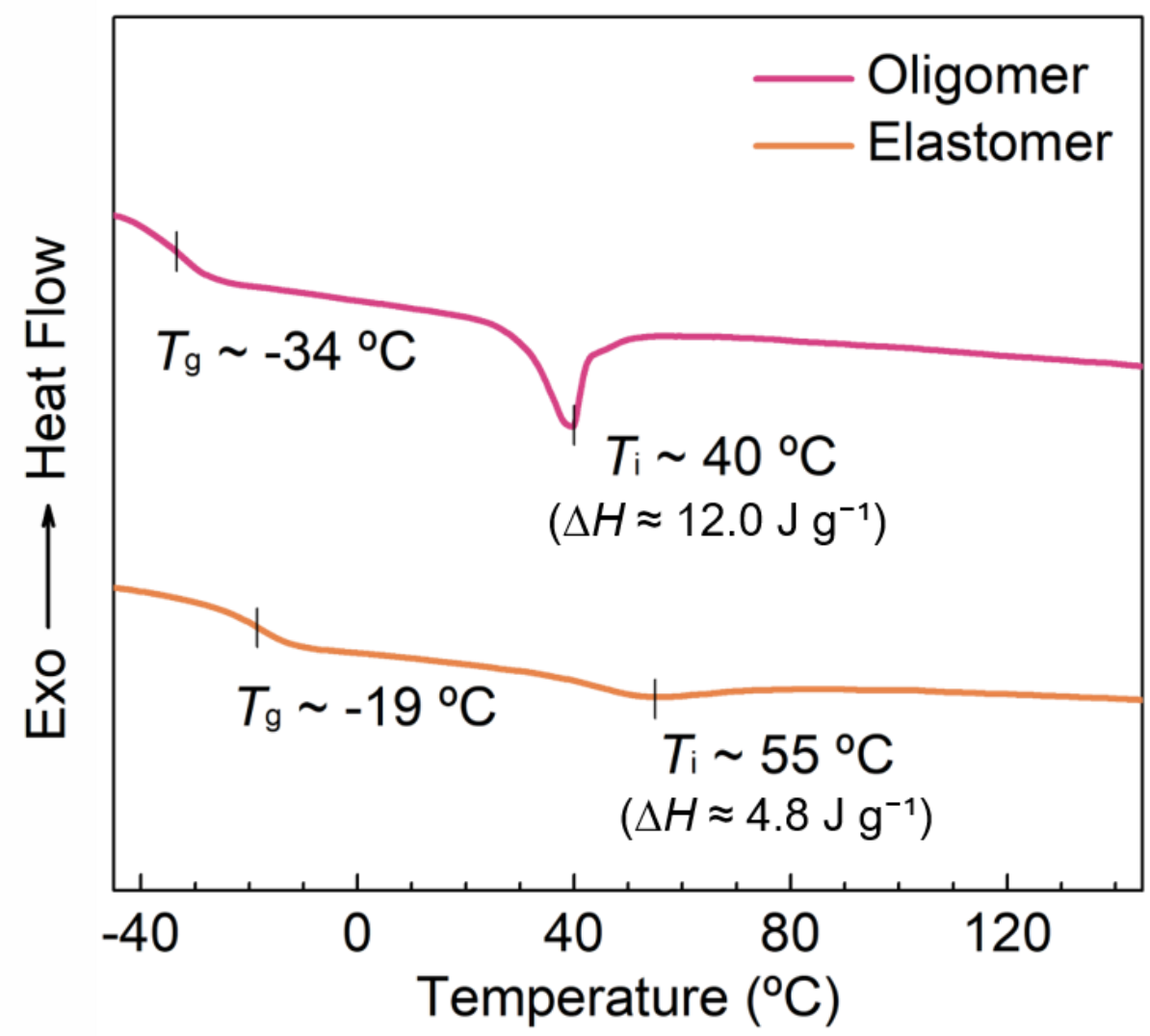

Supplementary Figure 4 | Differential scanning calorimetry (DSC) curves of smectogenic ink (oligomer) and cured LCE (elastomer) during the second heating cycle. Measurements were performed between -50 ºC and 150 ºC at a rate of 10 ºC min^{-1} under nitrogen.

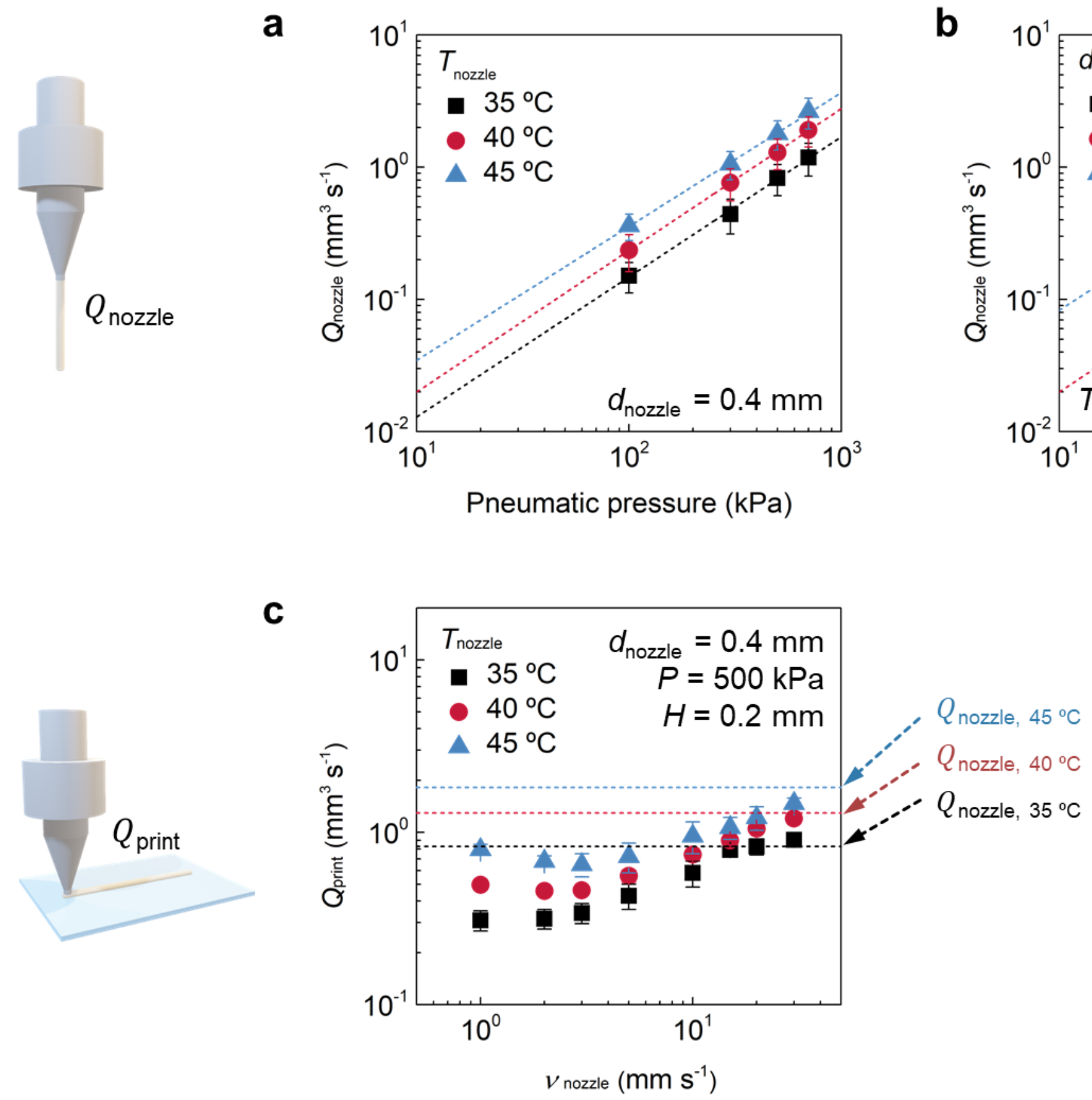

Supplementary Figure 5 | The volumetric flow rate (Q) of smectic ink during free extrusion (Q_{nozzle}) and during printing (Q_{print}). a, Q_{nozzle} as a function of applied pneumatic pressure (P) at different print temperatures (T_{nozzle}) using a 0.4 mm diameter nozzle. **b,** Q_{nozzle} as a function of P for different nozzle diameters (d_{nozzle}). **c,** Q_{print} as a function of nozzle translation speed (v_{nozzle}) at different T_{nozzle} under the conditions H = 0.2 mm, P = 500 kPa, and d_{nozzle} = 0.4 mm. Dashed lines indicate the corresponding Q_{nozzle} values at P = 500 kPa for each temperature, shown as reference to highlight that Q_{print} remains below Q_{nozzle} across all tested v_{nozzle} conditions. All Q values were measured using a gravimetric method. The values and the error bars represent the mean and the standard deviation, respectively ($n = 3$).

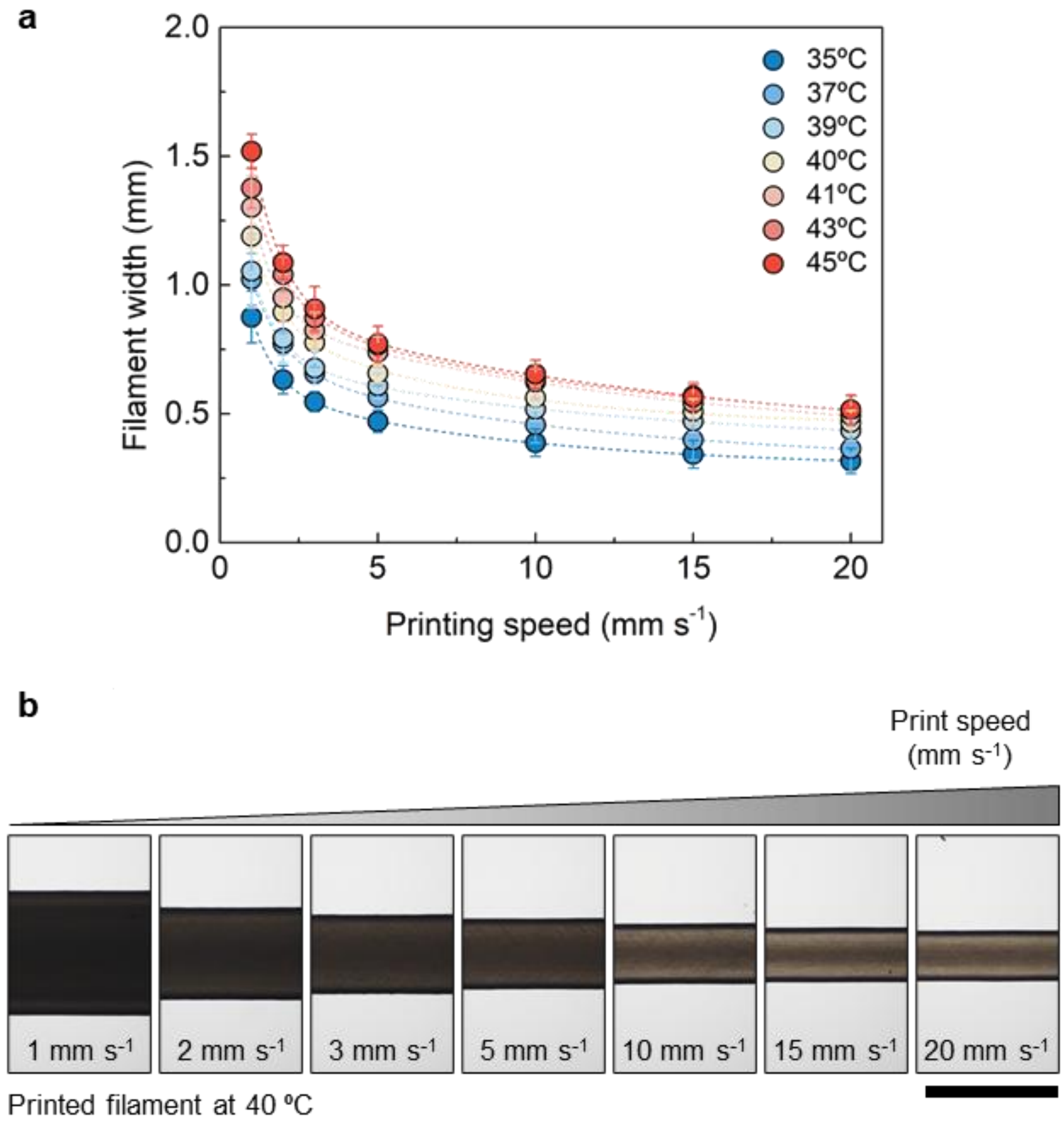

Supplementary Figure 6 | Printed filament width measured using optical microscopy. a, Filament width printed at various temperatures as a function of print speed. Solid markers represent the mean values, error bars indicate standard deviations (*n* = 3), and dashed lines show exponential decay fits to the mean values. **b,** Representative optical microscope images of filaments printed at 40 ºC with varying print speeds. Scale bar = 500 µm.

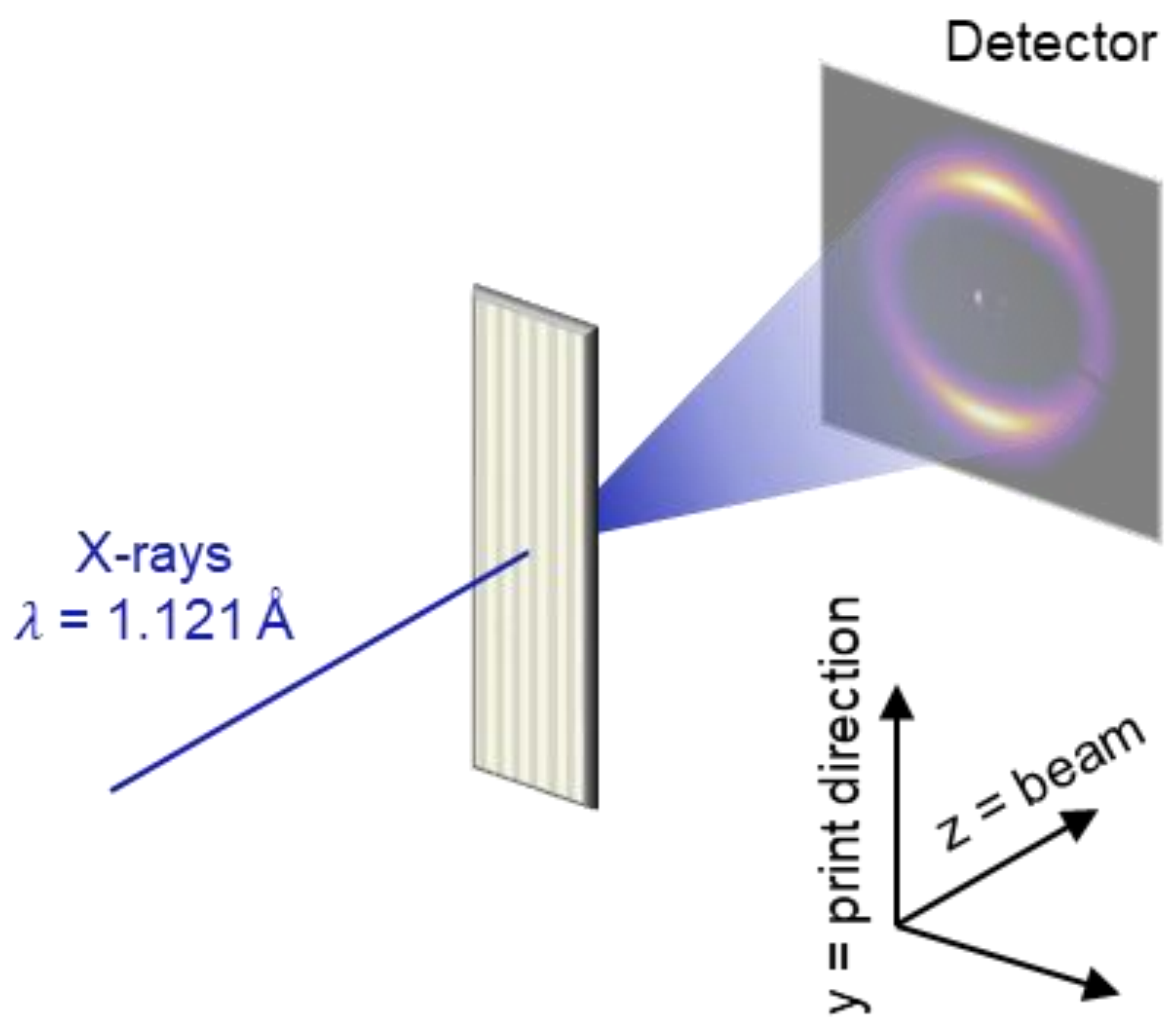

Supplementary Figure 7 | Schematic of the wide-angle X-ray scattering (WAXS) setup. The printed LCE films were mounted with the print direction aligned vertically, and X-rays were applied along the surface normal of the films.

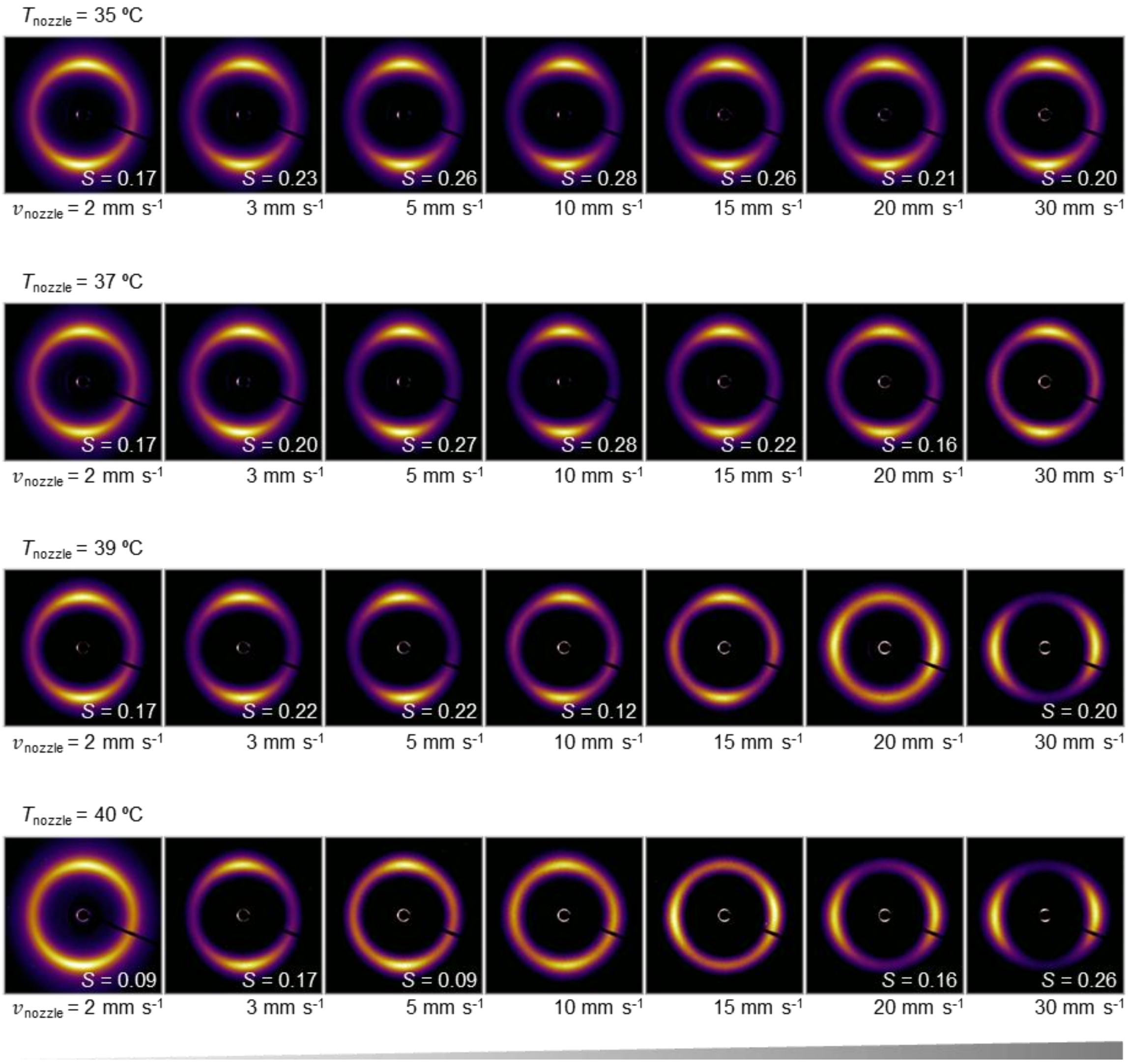

Supplementary Figure 8 | 2D wide-angle X-ray scattering (WAXS) patterns of LCEs printed at 35, 37, 39, and 40 ºC with different print speeds. T_{nozzle}, v_{nozzle}, and S represent print temperature, speed, and order parameter, respectively. The vertical direction corresponds to the print direction of LCEs, and X-ray irradiation was applied along the surface normal of the LCE films.

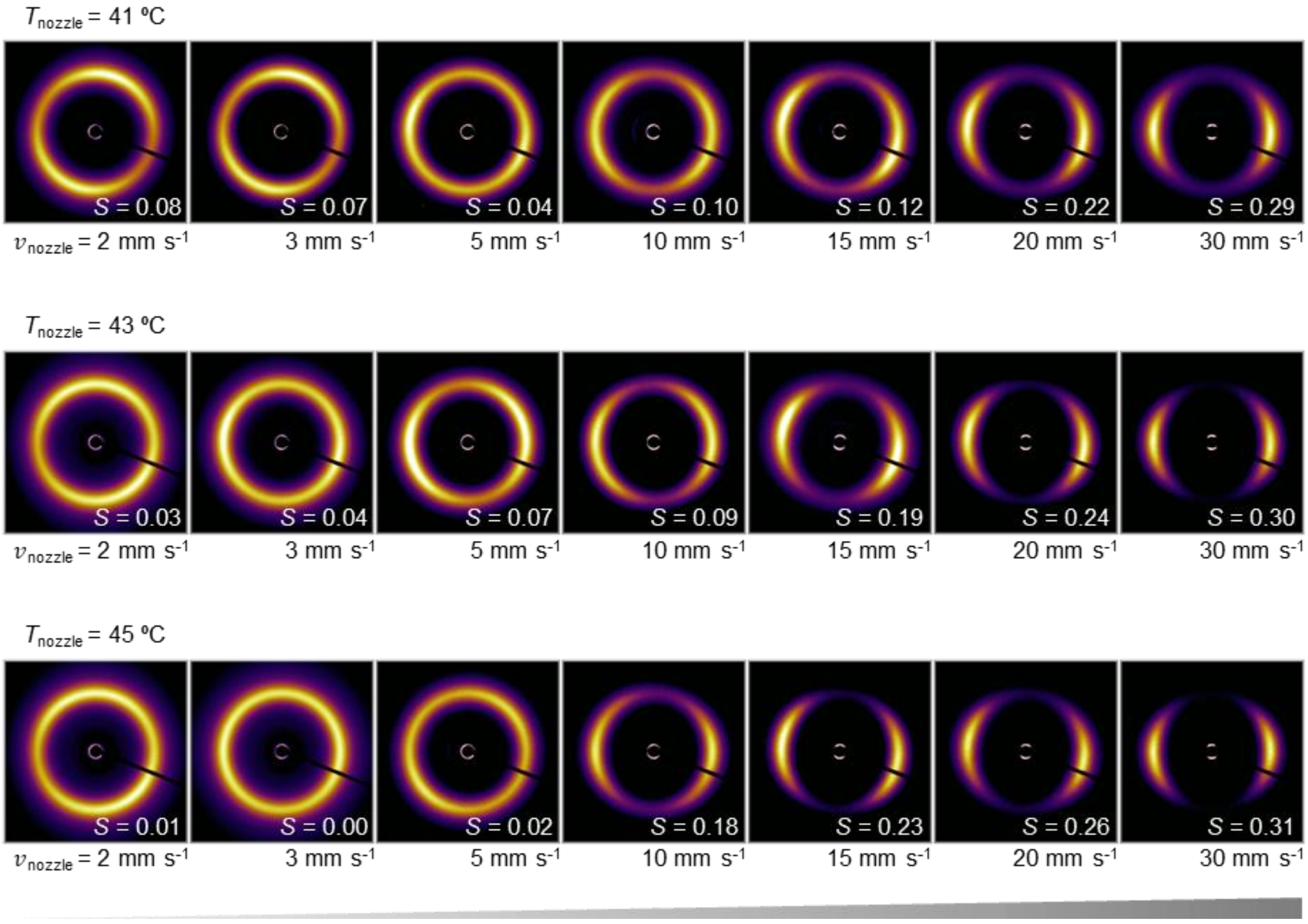

Supplementary Figure 9 | 2D WAXS patterns of LCEs printed at 41, 43, and 45 ºC with different print speeds. T_{nozzle}, v_{nozzle}, and S represent print temperature, speed, and order parameter, respectively. The vertical direction corresponds to the print direction of LCEs, and X-ray irradiation was applied along the surface normal of the LCE films.

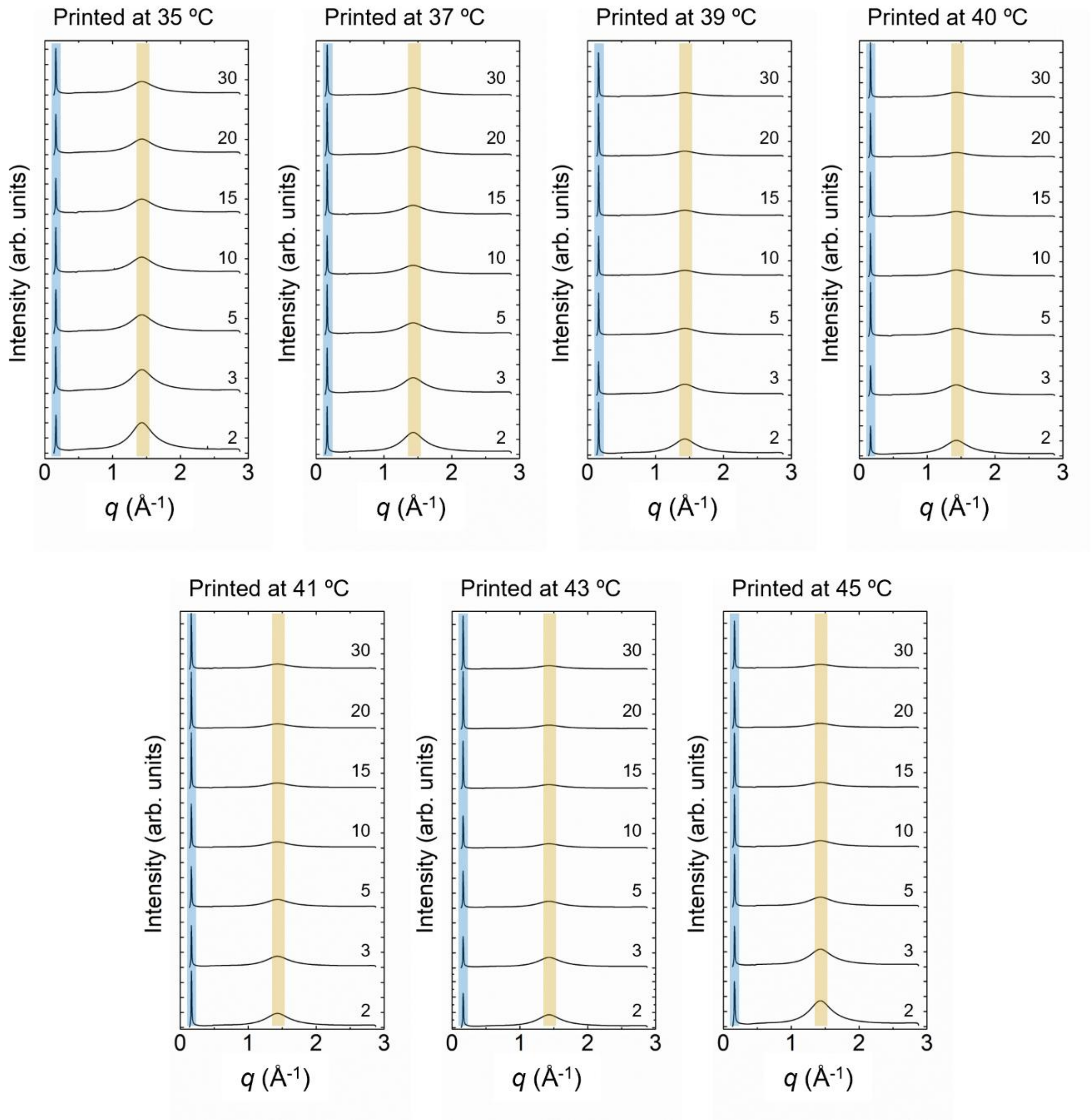

Supplementary Figure 10 | Scattering intensity of LCEs printed at varying temperatures and print speeds as a function of *q* vector, averaged over azimuthal angles. Each graph shows seven 1D profiles for samples printed at 2, 3, 5, 10, 15, 20, and 30 mm s^{-1}, from bottom to top. All samples exhibit a sharp peak at $q \approx 0.17$ $Å^{-1}$ corresponding to smectic layer spacing (~37.0 Å, blue box) and a broad peak at $q \approx 1.43$ $Å^{-1}$ corresponding to inter-mesogen spacing (~4.4 Å, yellow box).

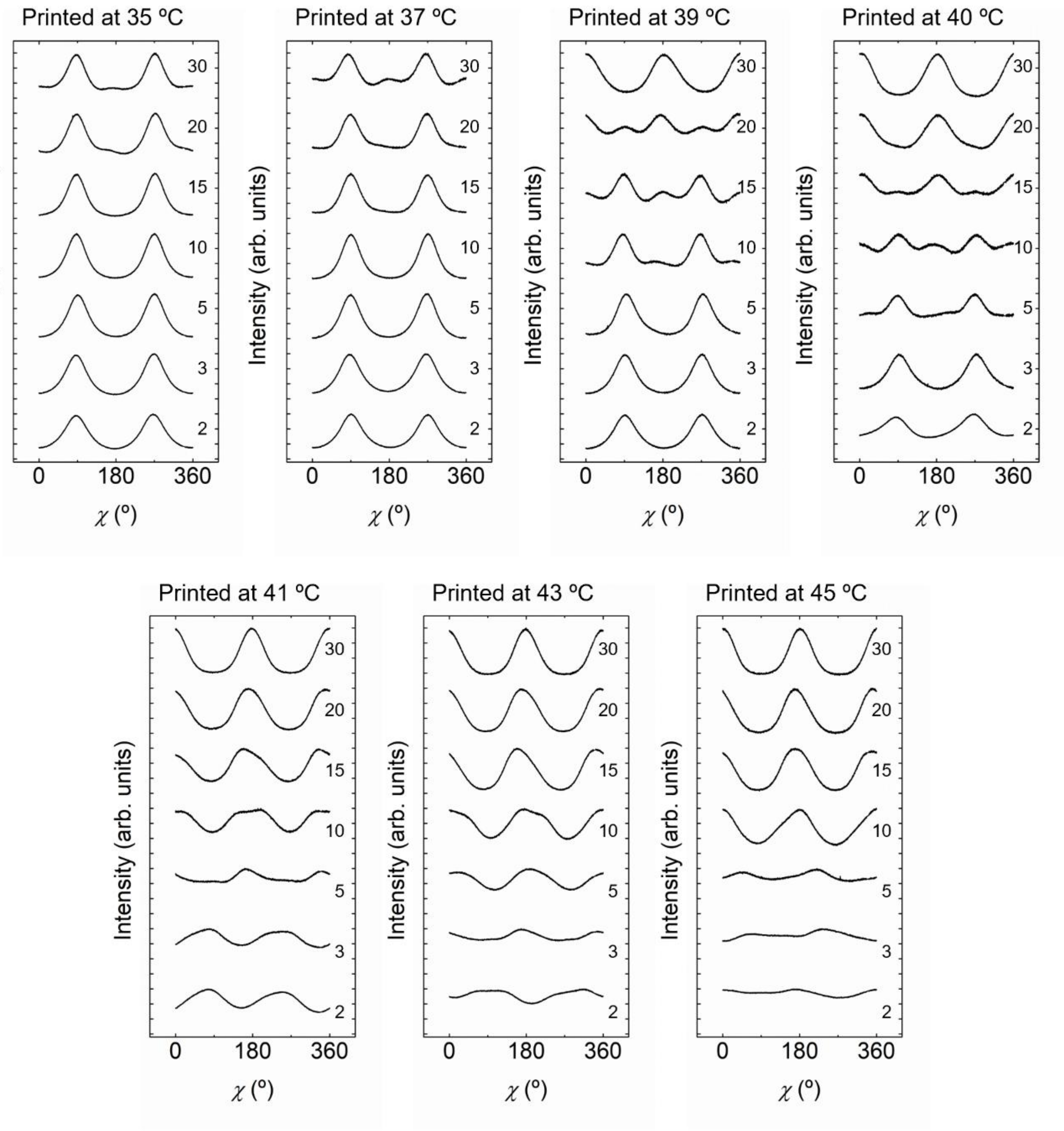

Supplementary Figure 11 | Azimuthal scan profiles of LCEs printed at varying temperatures and print speeds, corresponding to WAXS measurements. Scans were conducted at $q \approx 1.43$ Å^{-1}. Each graph shows seven azimuthal intensity profiles, representing samples printed at 2, 3, 5, 10, 15, 20, and 30 mm s^{-1}, from bottom to top.

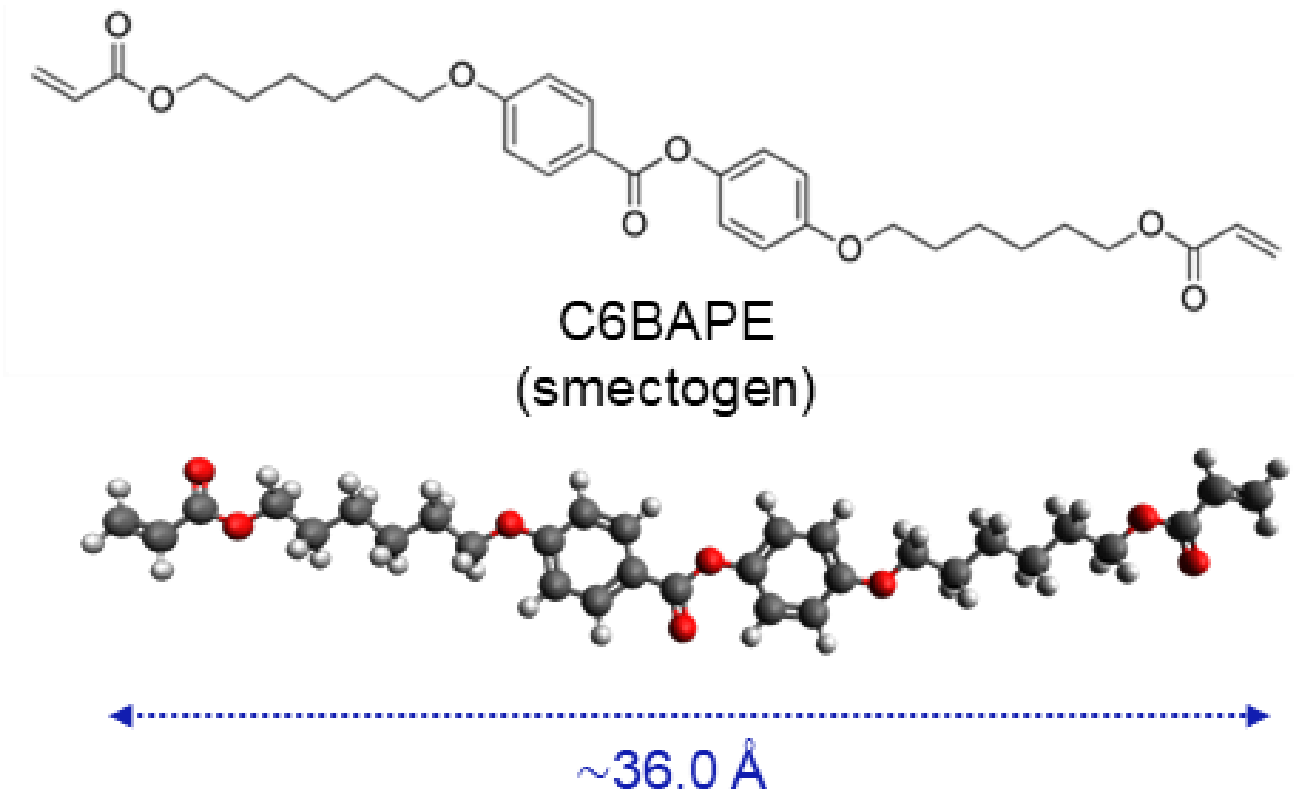

Supplementary Figure 12 | Molecular simulation of the smectogen. The molecular structure of C6BAPE was built using Avogadro-1.2.0n-win32 and subjected to energy minimisation. The molecular length was calculated based on the straight-line distance between the two terminal carbon atoms.

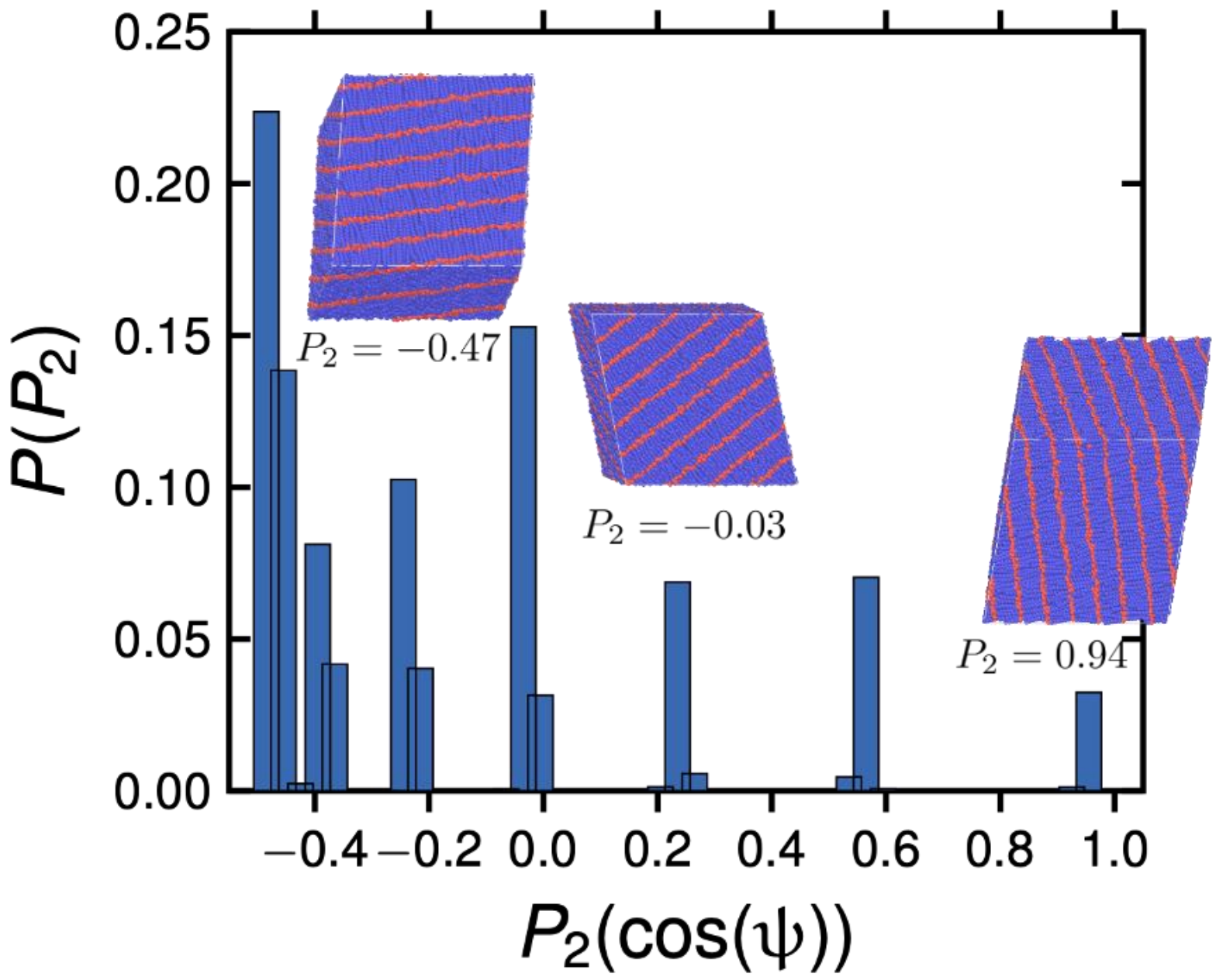

Supplementary Figure 13 | Distributions of $P_2(\cos(\psi))$ obtained from equilibrated MD simulations, used as the starting configurations for the NEMD simulations. Snapshots illustrate representative configurations corresponding to different $P_2(\cos(\psi))$ values.

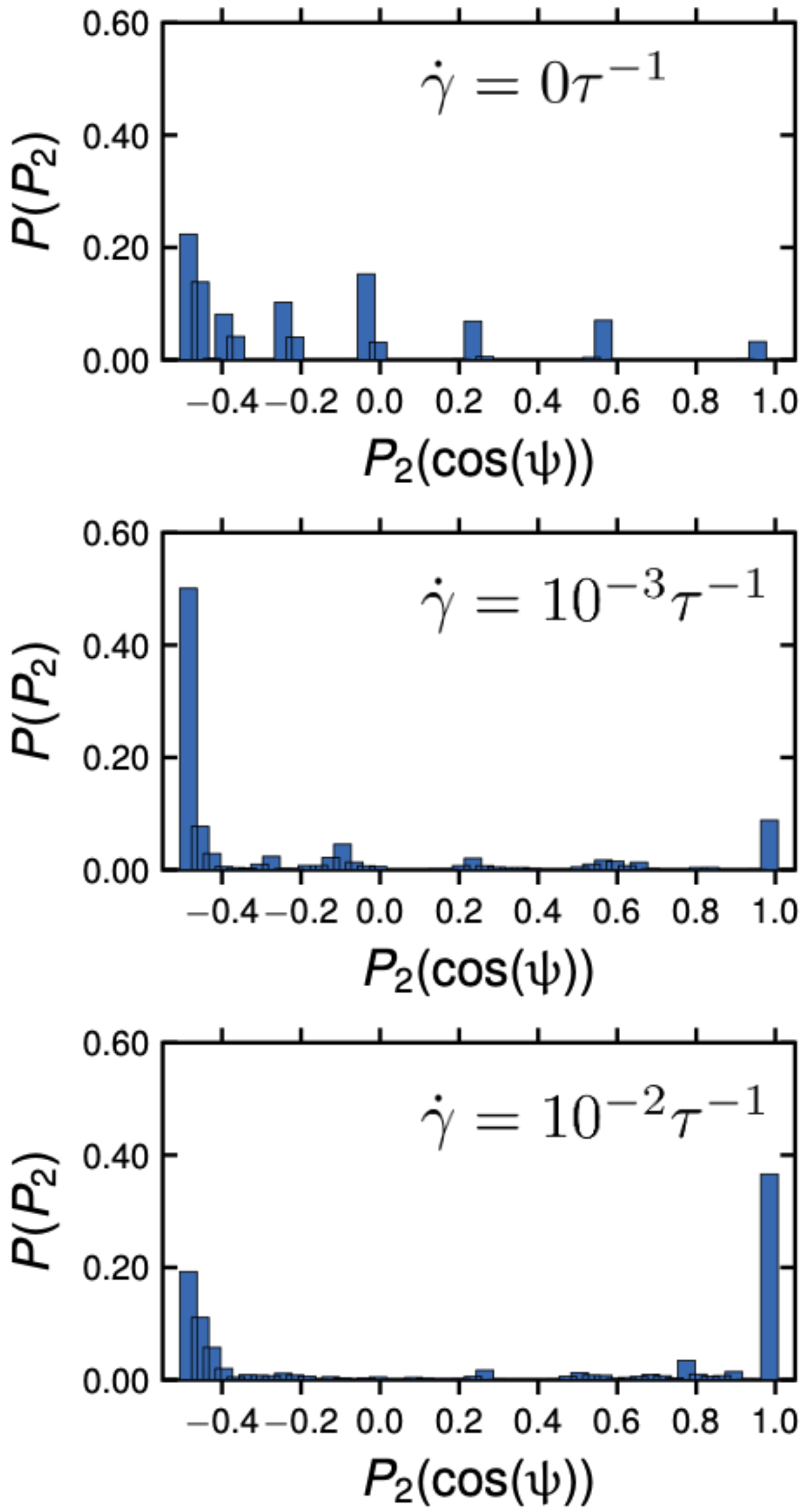

Supplementary Figure 14 | Distributions of $P_2(cos(\psi))$ at shear rates of $\dot{\gamma} = [0, 10^{-3}, 10^{-2}]\ \tau^{-1}$. $\dot{\gamma} = 0\ \tau^{-1}$ represents the initial state. At $\dot{\gamma} = 0.001\ \tau^{-1}$, well below the critical shear rate ($\dot{\gamma}$*), most domains adopt perpendicular orientation to the flow ($P_2 \approx 0.5$). At $\dot{\gamma} = 0.01\ \tau^{-1}$, the distribution begins to invert with many domains aligning parallel to the flow ($P_2 \approx 1.0$).

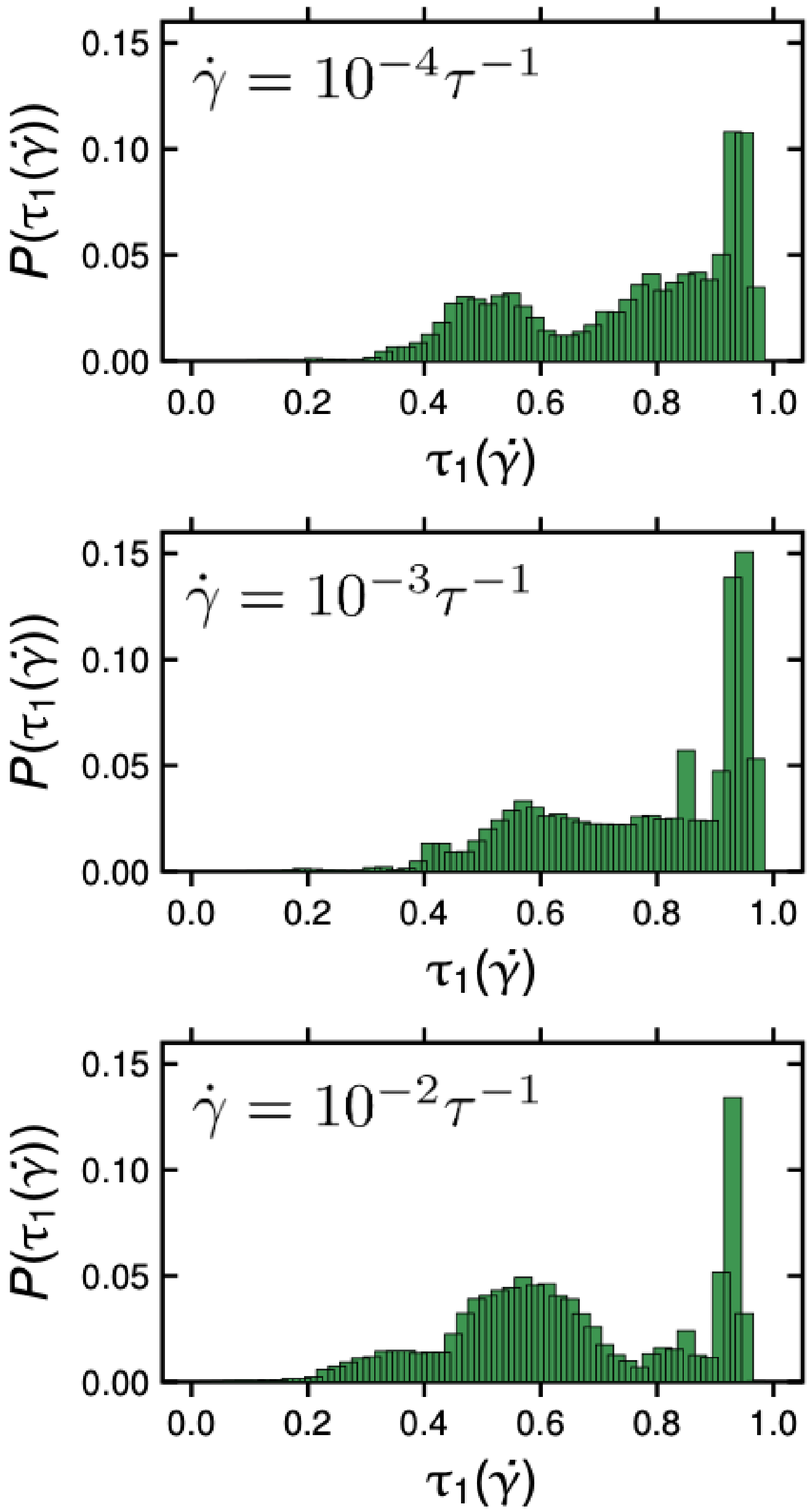

Supplementary Figure 15 | Distributions of $\tau_1(\dot{\gamma})$ at shear rates of $\dot{\gamma} = [10^{-4}, 10^{-3}, 10^{-2}]\ \tau^{-1}$. At $\dot{\gamma} = 0.001\ \tau^{-1}$, a larger fraction of τ_1 values approach 1.0, indicating enhanced smectic layering at this shear rate, consistent with the interpretation that the smectic layers predominantly slide past one another, constraining the mesogens within layers.

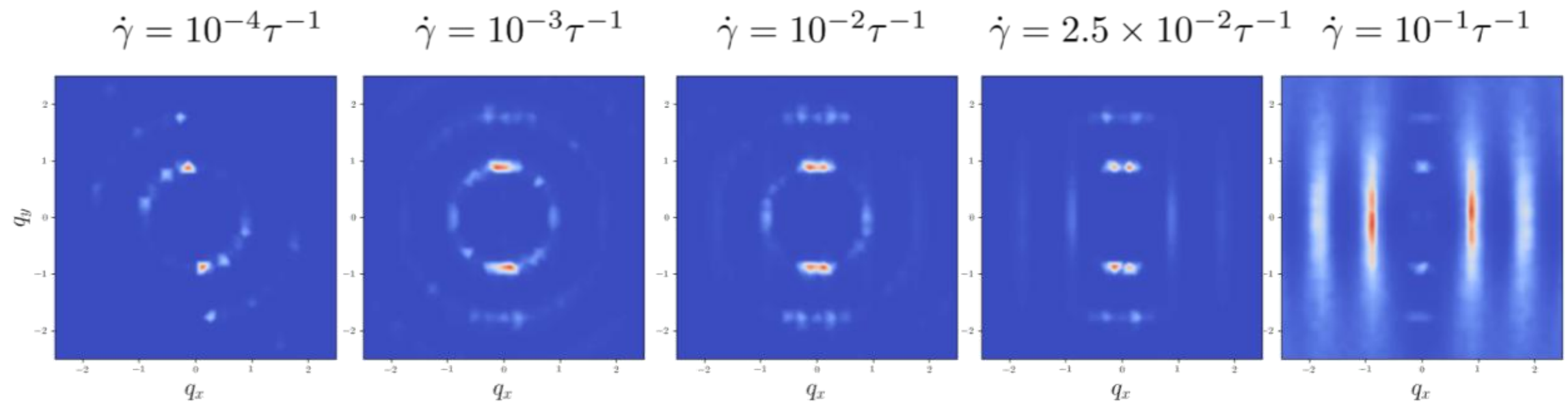

Supplementary Figure 16 | Scattering functions in the (q_x, q_y) plane from NEMD simulations across increasing shear rates. Left to right: low to high shear rate. At low shear, the scattering displays a ring with intensity maxima at $(0, \pm q^*)$, characteristic of a perpendicular smectic orientation. As shear increases, additional maxima appear at $(\pm q^*, 0)$, indicating the coexistence of perpendicular and parallel smectic orientations. At the highest shear rate, the smectic ring disappears and the scattering transitions to two elongated intensity lobes at $(\pm q^*, 0)$, reflecting a nematic state with strong flow-aligned mesogens and loss of smectic layer order.

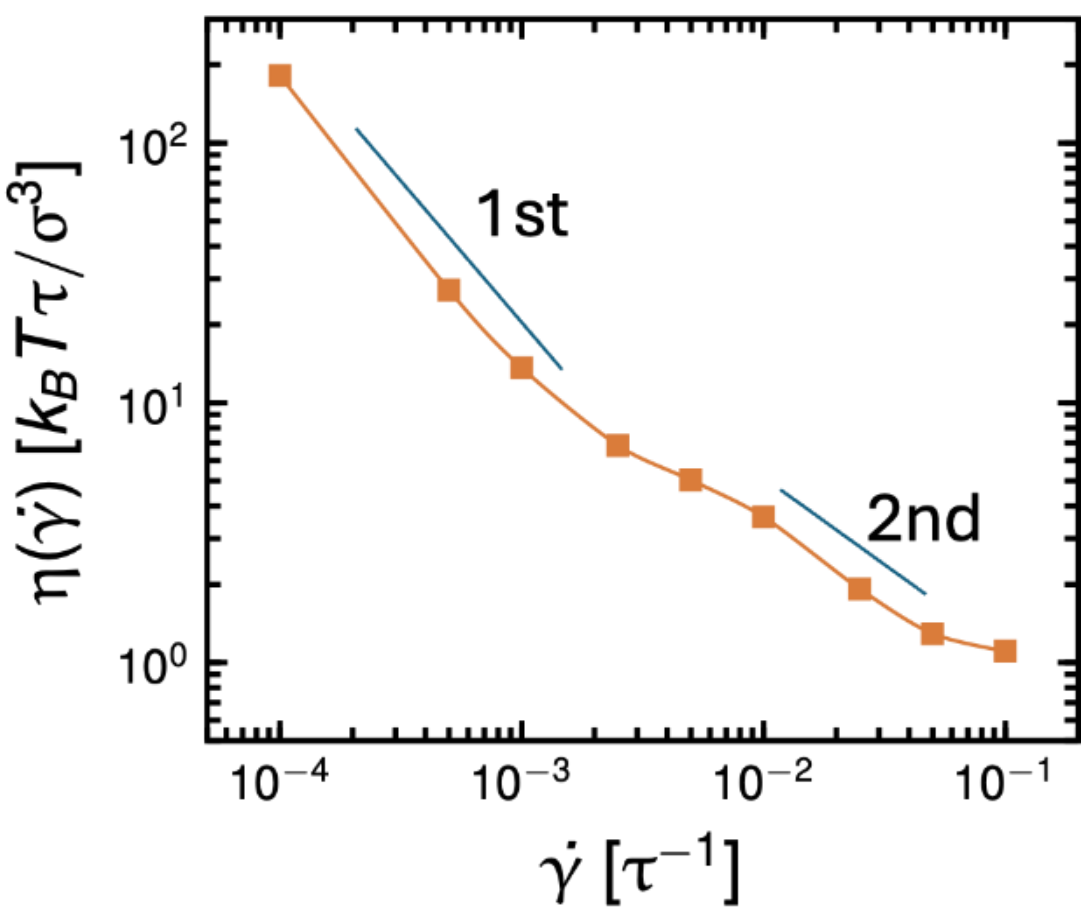

Supplementary Figure 17 | Shear-dependent viscosity $\eta(\dot{\gamma})$ obtained from NEMD simulations under steady simple shear. For each shear rate $\dot{\gamma}$, the viscosity is calculated from the averaged shear stress according to $\eta(\dot{\gamma}) = \langle \tau_{xz} \rangle / \dot{\gamma}$, where $\langle \tau_{xz} \rangle$ is the time- and ensemble-averaged xz component of the stress tensor, averaged over 100 independent simulations. The resulting viscosity curve displays two distinct shear-thinning regimes, similar to the behavior observed experimentally in rheology measurements. Each shear-thinning regime corresponds to a structural transition identified in the scattering patterns, from perpendicular smectic alignment to coexistence of orientations, and ultimately to a predominantly parallel (nematic) orientation at high shear rates.

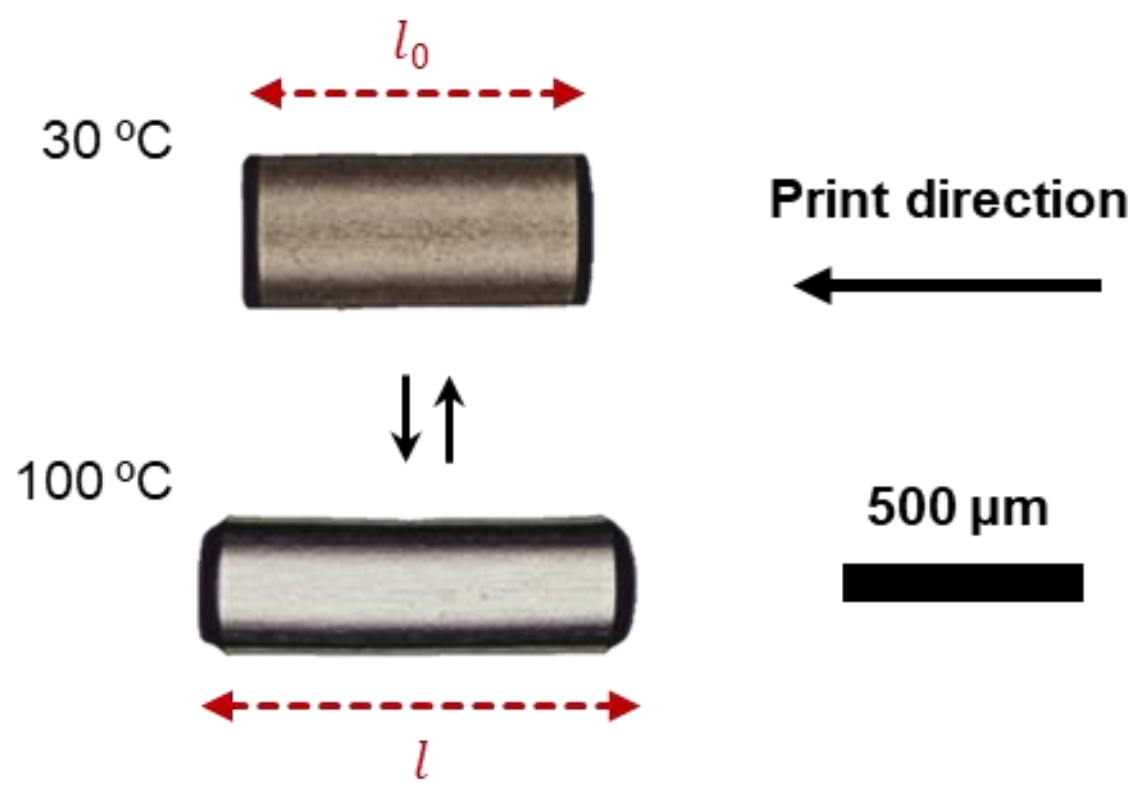

Supplementary Figure 18 | Actuation strain measurement of printed filaments using an optical microscope. The actuation strain, ε_a(%), is defined as $\varepsilon_a = (l - l_0)/l_0 \times 100$, where l_0 and l are the filament length at 30 ºC and 100 ºC, respectively.

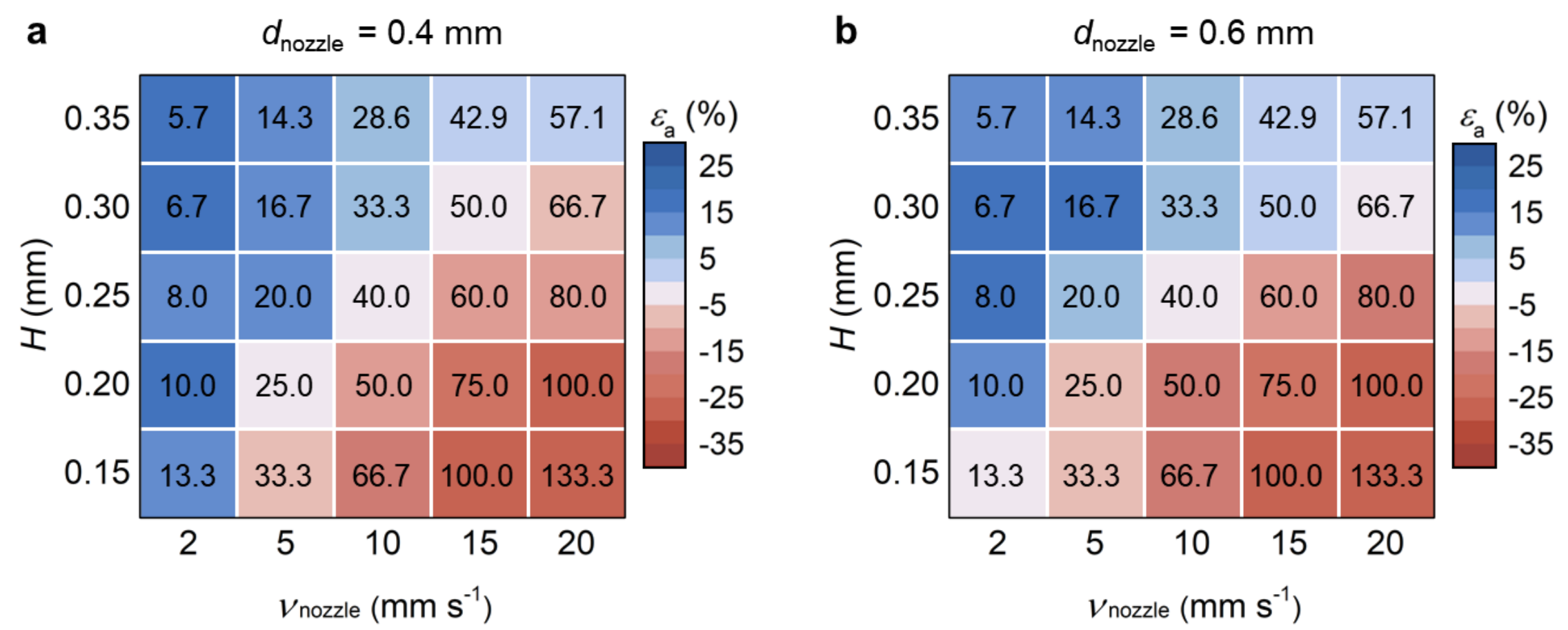

Supplementary Figure 19 | Effect of nozzle height (*H*) and nozzle diameter (d_{nozzle}) on alignment and actuation behavior. Heat maps of actuation strain (ε_a) at 100 °C for filaments printed at varying nozzle translation speeds (v_{nozzle}) and gap distances (*H*) under constant conditions of *P* = 500 kPa and T_{nozzle} = 40 °C, using 0.4 mm (**a**) and 0.6 mm (**b**) diameter nozzles. The values shown in each cell represent the applied shear rates ($\dot{\gamma} = v_{nozzle}/H$) at each condition.

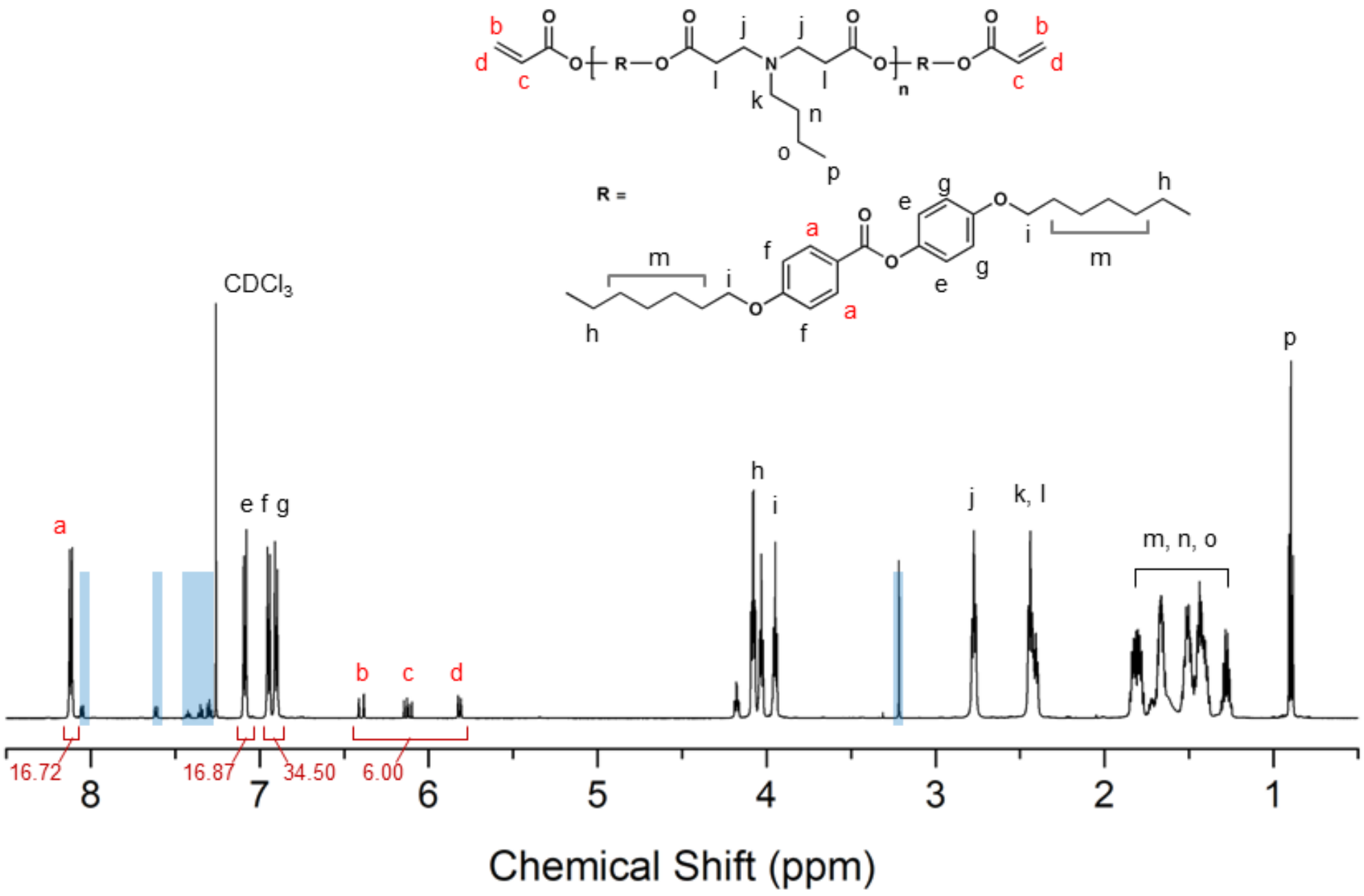

Supplementary Figure 20 | ^{1}H NMR spectra of the smectogenic ink with higher molecular weight (MW) recorded at 600 MHz in $CDCl_3$. The increased M_n was achieved by allowing further propagation of the ink via aza-Michael addition for 30 days at room temperature following synthesis. The light blue boxes highlight the peaks related to the photoinitiator (Irgacure 651) in the ink. The three peaks between 5.85 and 6.45 ppm (*b, c, d*) correspond to the six protons in the diacrylate end groups of the oligomer (CH_2=CHCOO–). The integration value of these peaks, set to six, was used to calibrate the other peaks. The peak at 8.12 ppm (*a*) corresponds to the two aromatic protons of the oligomer, with an integration of 16.72. From these values, the degree of polymerization (DP) was calculated as (16.72/2) – 1 = 7.36. The number-average molecular weight was then estimated as M_n = DP × M_n (repeating unit) + M_n (end groups) ≈ 7.36× 611.77 + 538.60 ≈ 5000 Da.

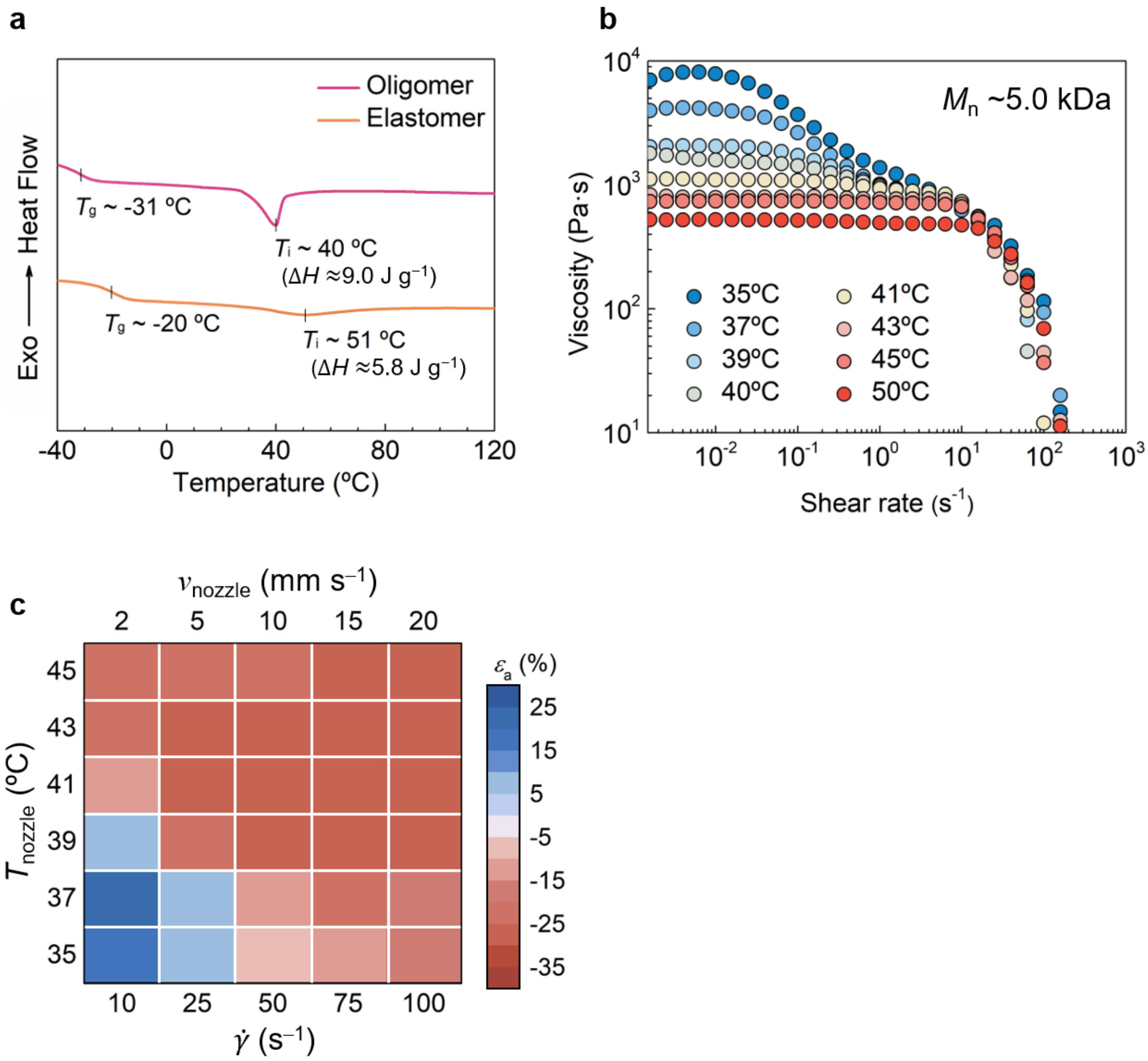

Supplementary Figure 21 | Influence of the molecular weight (MW) of the smectogenic ink on its alignment and actuation behavior. a, Differential scanning calorimetry (DSC) curves of smectogenic ink (oligomer, M_n ~5.0 kDa) and cured LCE during the second heating cycle. Measurements were performed between –50 ºC and 150 ºC at a rate of 10 ºC min^{-1} under nitrogen. **b**, Steady-shear viscosity as a function of shear rate at various temperatures for the oligomer. **c**, Heat map of actuation strain at 100 ºC for filaments printed at different temperatures and speeds, measured by optical microscopy. The shear rates ($\dot{\gamma}$) on the bottom axis are calculated from the corresponding v_{nozzle} values as $\dot{\gamma} = v_{nozzle}/H$, where H = 0.2 mm. The actuation strain, $\varepsilon_a(\%)$, is defined as $\varepsilon_a = (l - l_0)/l_0 \times 100$, where l_0 and l are the filament length at 30 ºC and 100 ºC, respectively.

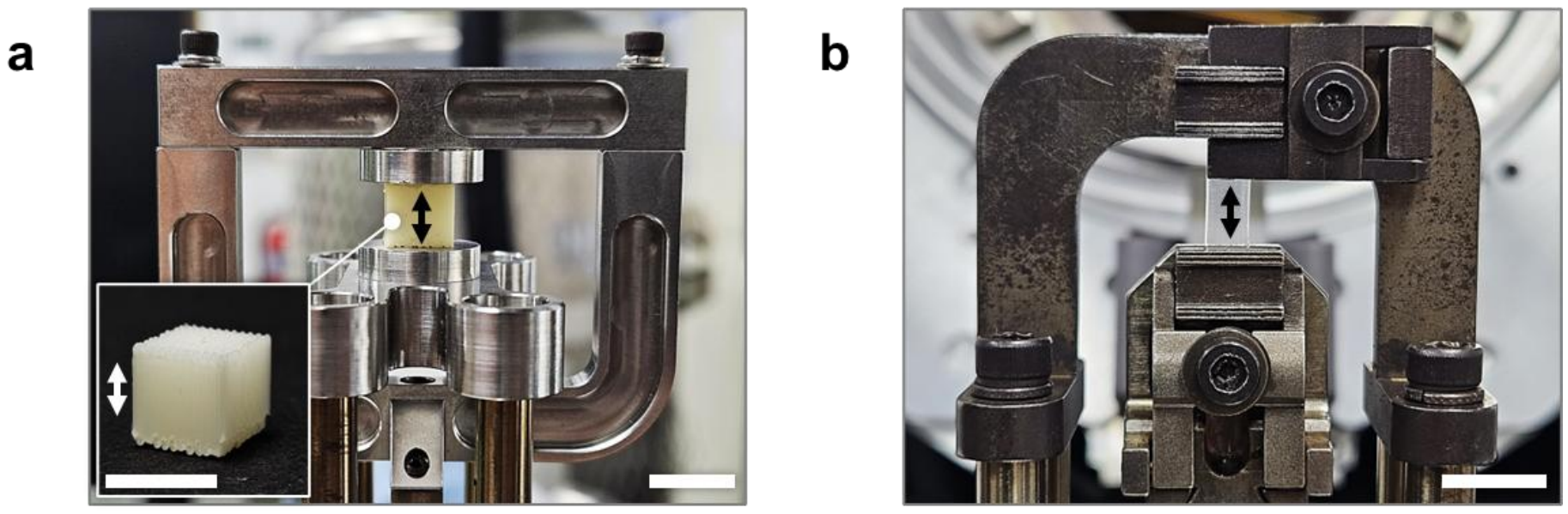

Supplementary Figure 22 | Isostrain testing setup. **a**, Compression-mode dynamic mechanical analysis used to measure the elongational blocking stress of an 8 × 8 × 8 mm^3 cube (inset) printed at T_{nozzle} = 35 °C and v_{nozzle} = 10 mm s^{-1}. **b**, Tensile-mode dynamic mechanical analysis used to measure the contractile blocking stress of a 15 × 5 × 0.2 mm^3 film printed at T_{nozzle} = 45 °C and v_{nozzle} = 20 mm s^{-1}. Bidirectional arrows indicate the print direction. Scale bars = 10 mm.

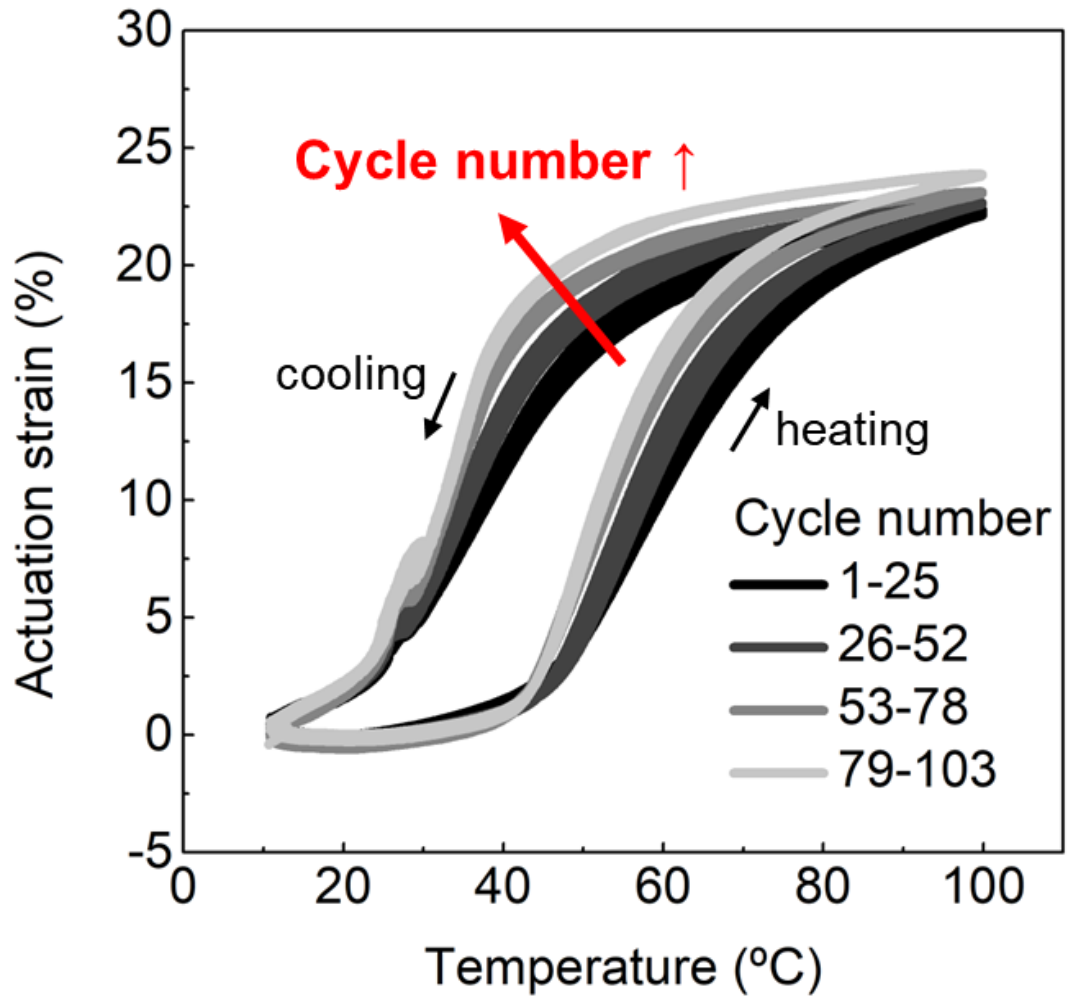

Supplementary Figure 23 | Thermal actuation strain of the LCE film printed at T_{nozzle} = 35 ºC and v_{nozzle} = 3 mm s^{-1} as a function of temperature during cyclic test. The thermal actuation strain was measured over 103 heating–cooling cycles between 10 and 100 °C at a rate of 5 °C min^{-1} under a constant applied stress of 1 kPa. Approximately 25 cycles could be performed with a fully filled gas cooling accessory (GCA); therefore, the GCA was refilled with liquid nitrogen before the 26th, 53rd, and 78th cycles to enable testing over 100 cycles. As the cycle number increased, the slope of the actuation curves became slightly steeper, while the maximum actuation strain changed only marginally, from 22.3% to 23.9% after 103 cycles.

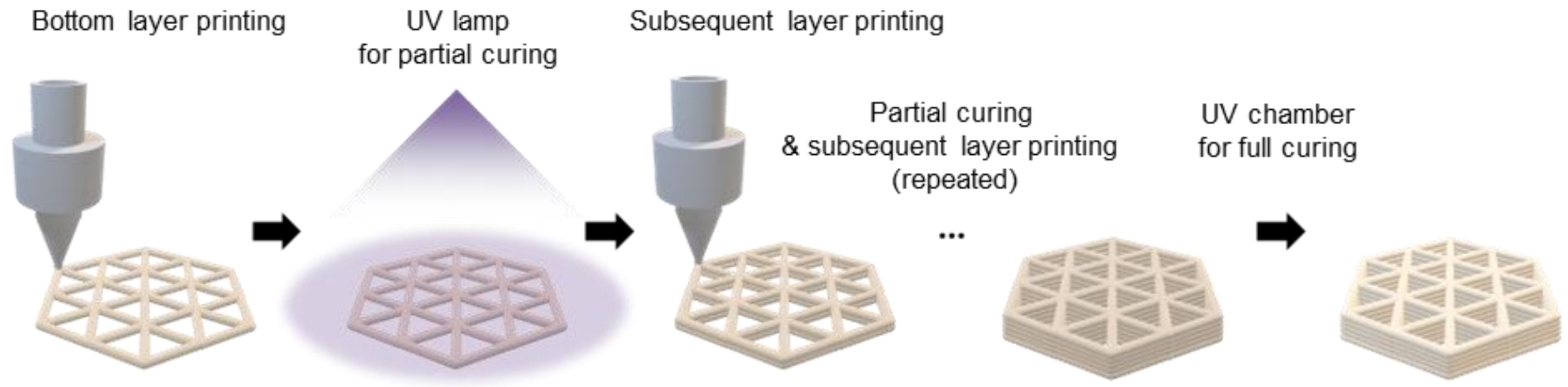

Supplementary Figure 24 | Schematic illustration of the multi-layer 3D printing process. Multi-layered structures were fabricated by layer-by-layer printing, with each layer exposed to weak UV light for 30 s for partial curing before stacking the next layer. After all layers were printed, the structures were fully cured in a UV chamber for 10 min.

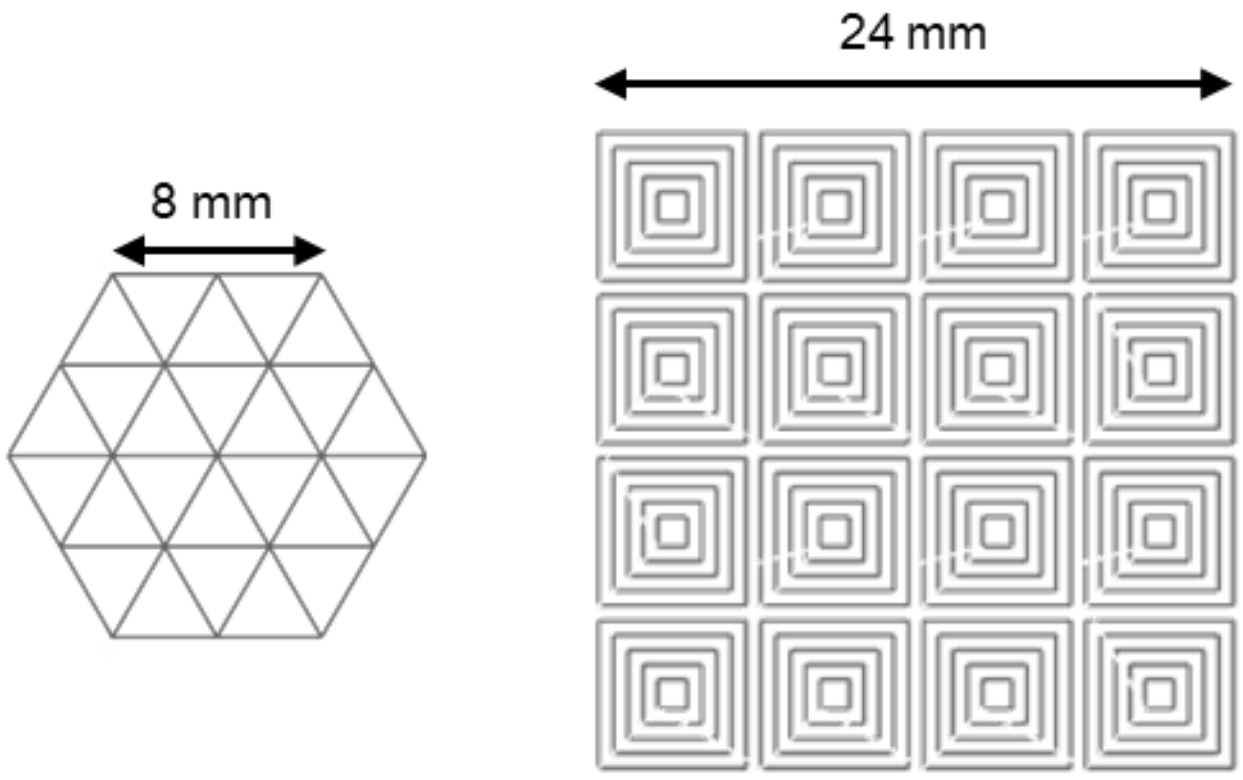

Supplementary Figure 25 | Print map for triangular lattice and 4 × 4 concentric square array. The triangular lattice structure consists of equilateral triangles with 4 mm sides (left), while the concentric square array comprises squares with 6 mm sides (right).

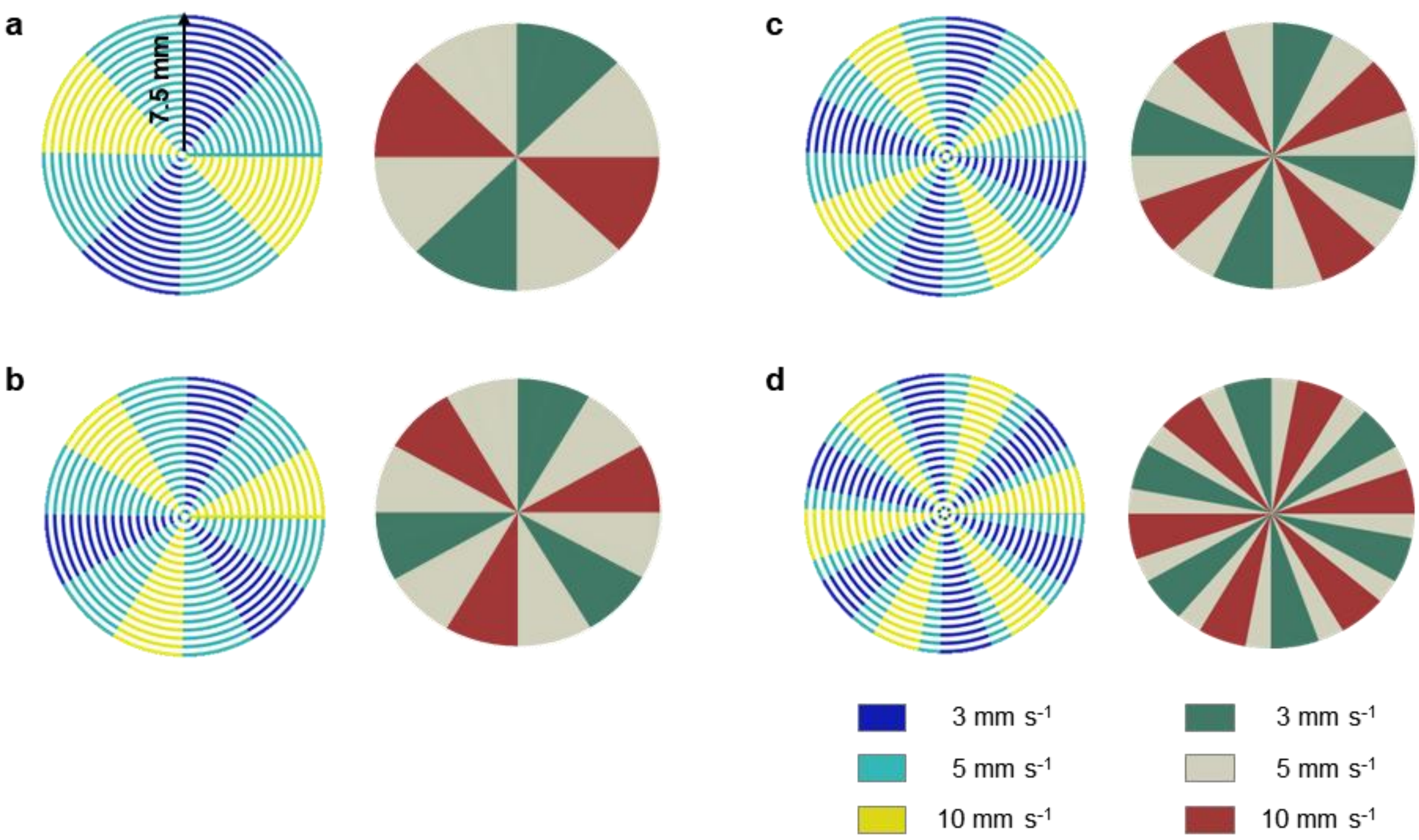

Supplementary Figure 26 | Concentric print paths (left) and corresponding FEA model (right) with stepwise variation in print speed along azimuthal or radial directions. a, Azimuthally programmed path with v_{nozzle} of 3, 5, 10, and 5 mm s^{-1} across 0–45°, 45–90°, 90–135° and 135–180°, repeated around the full circle. **b**, Azimuthally programmed path with v_{nozzle} of 3, 5, 10, and 5 mm s^{-1} across 0–30°, 30–60°, 60–90° and 90–120°, repeated around the full circle. **c**, Azimuthally programmed path with v_{nozzle} of 3, 5, 10, and 5 mm s^{-1} across 0–25°, 25–45°, 45–70° and 70–90°, repeated around the full circle. **d**, Azimuthally programmed path with v_{nozzle} of 3, 5, 10, and 5 mm s^{-1} across 0–10°, 10–30°, 30–40° and 40–60°, repeated around the full circle.

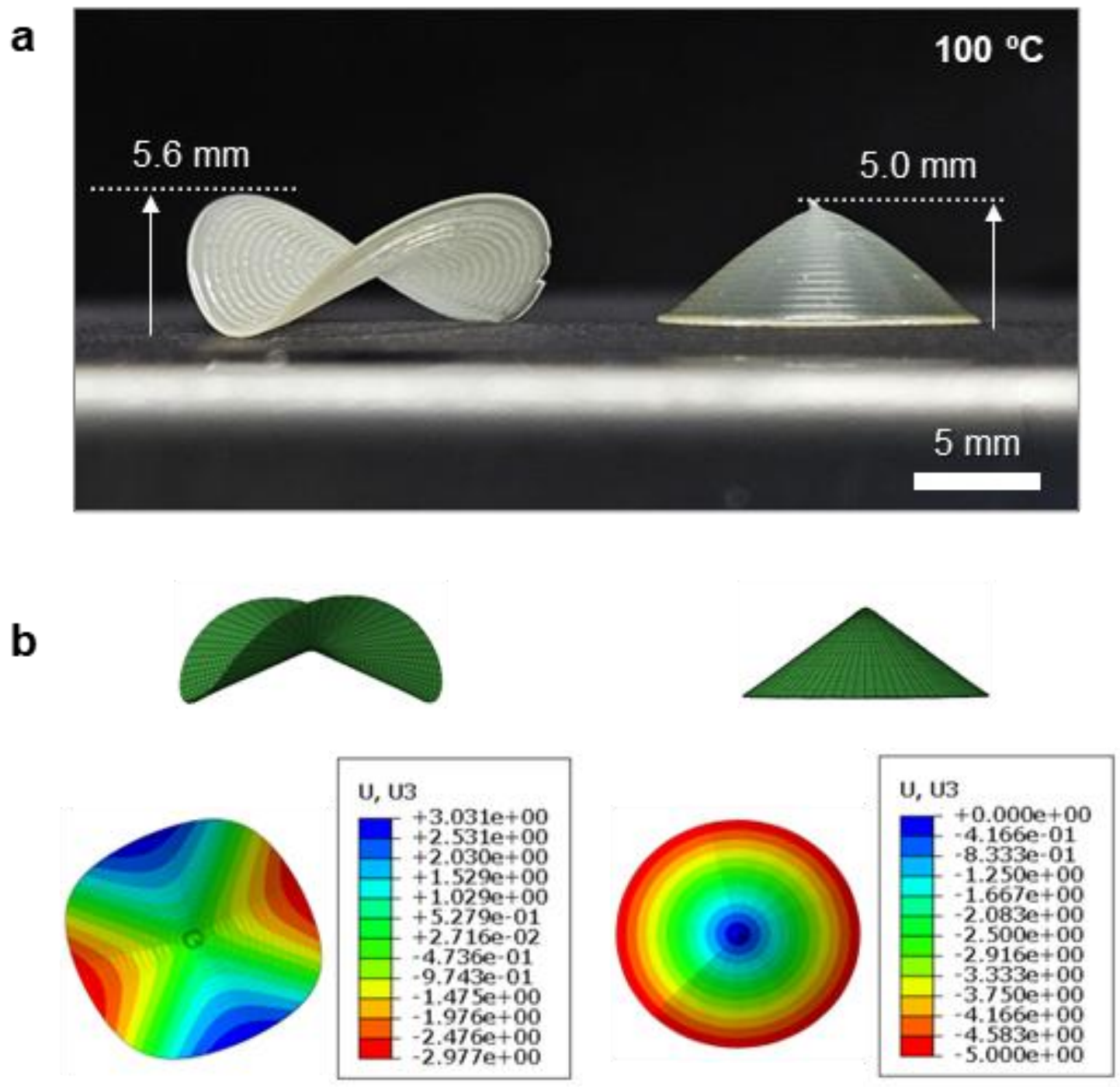

Supplementary Figure 27 | Comparison of vertical displacement between experimental measurements and finite element analysis (FEA) of thermal deformation. a, Thermal deformation of LCE discs printed along concentric paths at print speeds of 3 mm s^{-1} (left) and 10 mm s^{-1} (right). **b**, Corresponding FEA results showing deformed shapes and vertical displacement fields (unit in mm). In the FEA, fixed boundary conditions were applied to the central region of the mesh.

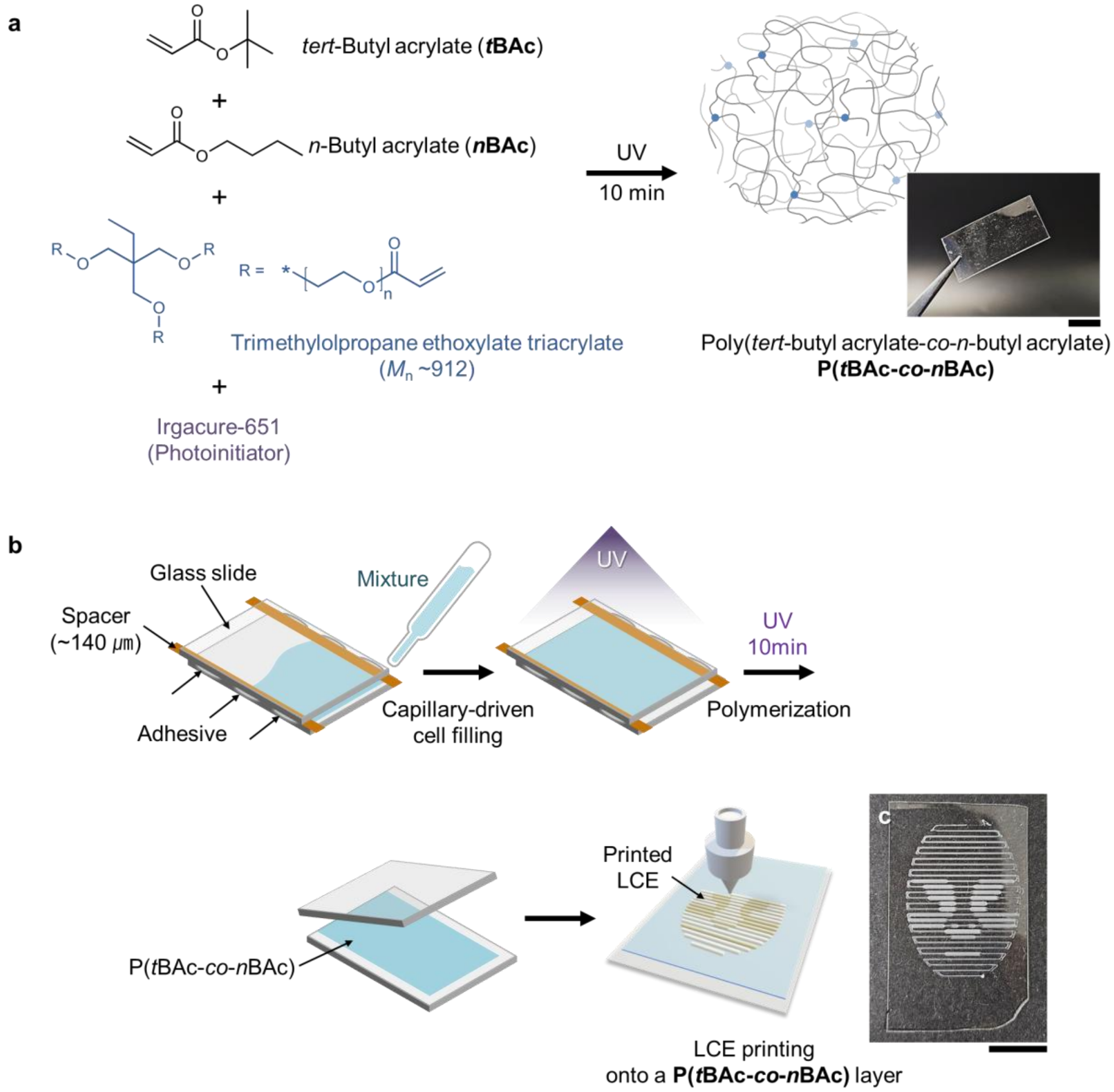

Supplementary Figure 28 | Fabrication of bilayer 3D-printed structures. a, Synthetic process of the flexible SMP substrate, composed of poly(*tert*-butyl acrylate-*co*-*n*-butyl acrylate). *t*BAc, *n*BAc, crosslinker, and photoinitiator were mixed at a weight ratio of 83.3:14.7:1.0:1.0 and thoroughly vortexed at ambient temperature. The precursor mixture was then polymerized under UV irradiation for 10 min. **b**, Preparation of the SMP film and LCE DIW onto the film. The precursor mixture from (**a**) was injected into a glass cell with a 140 μm gap and filled by capillary action. The cell was then transferred to a UV chamber and polymerized under UV irradiation for 10 min. After polymerization, the top glass slide was removed, and the film attached to the bottom glass slide was used as the substrate for fabricating bilayer 3D-printed structures. Scale bars = 10 mm.

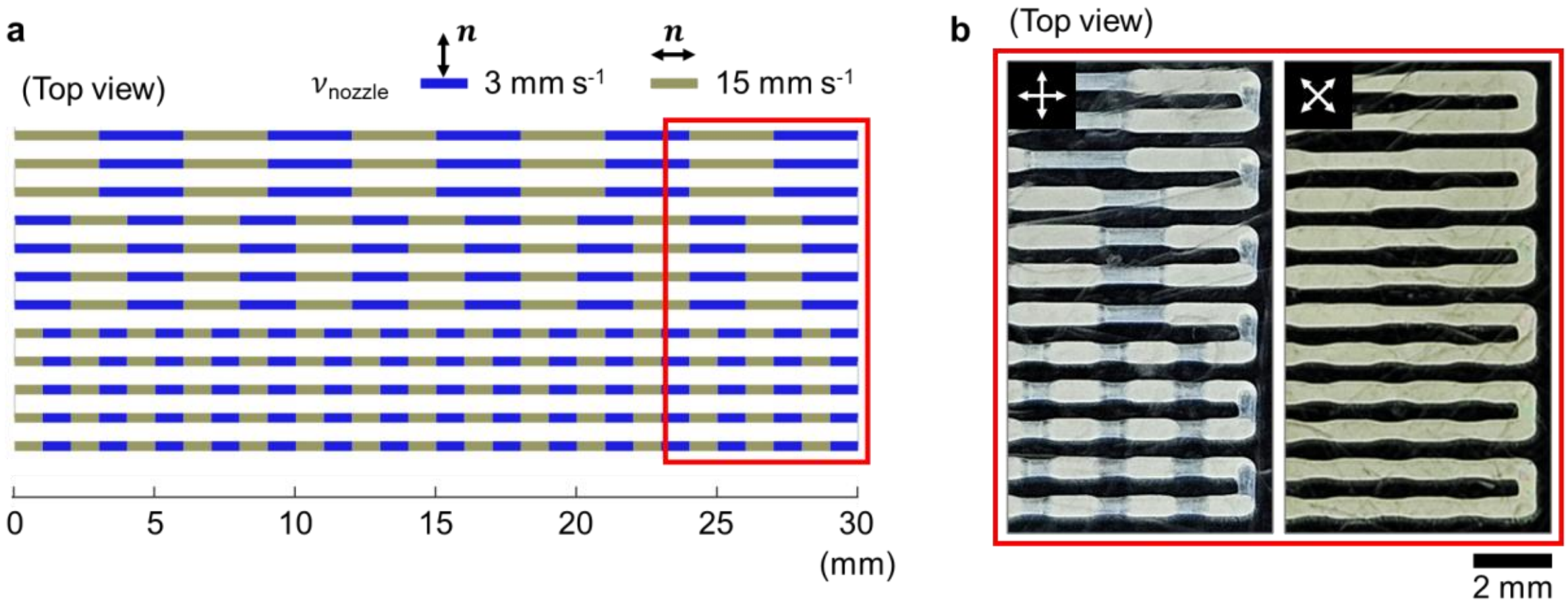

Supplementary Figure 29 | Smectic LCE filaments spatially programmed with alternating director patterns of different section lengths via encoded print speeds (v_{nozzle}). a, Print path with encoded print speeds. Double arrows indicate the director ($\vec{n}$). **b,** Polarized optical image of the printed filament acquired between crossed polarizers aligned (left) parallel to and (right) rotated by 45° with respect to the filament axis. Sections printed at $v_{nozzle} = 3$ mm s^{-1} and $v_{nozzle} = 15$ mm s^{-1} exhibit different birefringence intensities. White arrows indicate the orientations of the polarizer and analyzer.

Supplementary Table 1. Actuation strain values* at 100 ºC for filament printed under different temperatures and speeds, measured using optical microscopy.

v_{nozzle} (mm s^{-1}) / T_{nozzle} (ºC)	2	5	10	15	20
**35**	22.9 ± 3.1	23.3 ± 1.9	25.6 ± 1.2	20.1 ± 1.0	18.5 ± 1.8
**37**	18.2 ± 4.8	18.7 ± 2.6	18.5 ± 1.5	13.6 ± 3.4	6.2 ± 3.9
**39**	12.6 ± 0.03	4.1 ± 0.05	-4.7 ± 0.05	-13.4 ± 0.06	-21.2 ± 0.08
**41**	-10.5 ± 2.1	-18.7 ± 7.4	-30.4 ± 2.5	-30.7 ± 3.4	-31.2 ± 4.4
**43**	-13.4 ± 4.0	-17.0 ± 2.5	-24.4 ± 0.4	-30.8 ± 3.4	-34.5 ± 3.4
**45**	-8.0 ± 2.4	-18.4 ± 5.4	-21.0 ± 6.1	-26.1 ± 4.7	-30.2 ± 4.2

*Actuation strain, ε_a(%), is defined as $\varepsilon_a = (l - l_0)/l_0 \times 100$, where l_0 and l are the filament length at 30 ºC and 100 ºC, respectively.

Supplementary Table 2. Parameters used for CTE determination by curve fitting.

**Material**	**Print speed (mm s^{-1})**	L_1 **(mm)**	L_2 **(mm)**	L_3 **(1 °C^{-1})**	L_4 **(°C)**
Material 1	3	7.026	0.3672	0.0610	64.94
Material 2	5	6.989	0.1641	0.1040	64.93
Material 3	10	6.991	-0.6681	0.025	95.72

References

1. Milchev, A., Nikoubashman, A. & Binder, K. The smectic phase in semiflexible polymer materials: a large scale molecular dynamics study. *Comput. Mater. Sci.* **166**, 230–239 (2019).

2. Weeks, J. D., Chandler, D. & Andersen, H. C. Role of repulsive forces in determining the equilibrium structure of simple liquids. *J. Chem. Phys*. **54**, 5237–5247 (1971).

3. Kremer, K., & Grest, G. S. Dynamics of entangled linear polymer melts: a molecular-dynamics simulation. *J. Chem. Phys.* **92**, 5057–5086 (1990).

4. Thompson, A. P. *et al*. LAMMPS - a flexible simulation tool for particle-based materials modeling at the atomic, meso, and continuum scales. *Comput. Phys. Commun.* **271**, 108171–108204 (2022).

5. Carrillo, J.-M. Y. & Wang, Y. Shear-induced conformations of salt-free polyelectrolytes in semidilute solutions. *ACS Macro Lett.* **14**, 933–939 (2025).

6. Huang, G.-R. *et al*. An exact inversion method for extracting orientation ordering by small-angle scattering. *Phys. Chem. Chem. Phys.* **23**, 4120–4132 (2021).

7. Palermo, M. F., Pizzirusso, A., Muccioli, L. & Zannoni, C. An atomistic description of the nematic and smectic phases of 4-n-octyl-4′ cyanobiphenyl (8CB). *J. Chem. Phys.* **138**, 204901–204916 (2013).

8. Lopes, J. T., Romano, F., Grelet, E., Franco, L. F. M. & Giacometti, A. Phase behavior of hard cylinders. *J. Chem. Phys.* **154**, 104902–104913 (2021).

9. Tajbakhsh, A. R. & Terentjev, E. M. Spontaneous thermal expansion of nematic elastomers. *Eur. Phys. J. E* **6**, 181–188 (2001).

10. Warner, M. & Terentjev, E. M. Liquid Crystal Elastomers (Oxford Univ. Press, USA, 2007).

11. Wang, Z. *et al*. Three-dimensional printing of functionally graded liquid crystal elastomer. *Sci. Adv*. **6**, eabc0034 (2020).

12. Feng, X. *et al.* Melt electrowriting enabled 3D liquid crystal elastomer structures for cross-scale actuators and temperature field sensors. *Sci. Adv.* **10**, eadk3854 (2024).
