# Supplementary material for: Alignment switching in 3D-printed smectic liquid crystal elastomers
Source: Nat Commun. 2026 Jul 10;17:8530. doi: 10.1038/s41467-026-75368-z (PMC13483228; doi:10.1038/s41467-026-75368-z)
Supplement: Supplementary file 3 [file 41467_2026_75368_MOESM2_ESM.pdf]

Description of Additional Supplementary Files

Supplementary Movie 1. Actuation and load-bearing performance of 4 x 4 concentric square arrays printed at 35 ºC.

Supplementary Movie 2. Actuation and load-bearing performance of 4 x 4 concentric square arrays printed at 43 ºC.

Supplementary Movie 3. Triangular LCE lattices with five layers printed at 35 ºC and 43 ºC.

Supplementary Movie 4. Programmed deformation of triangular LCE lattices into a gear-like shape.

Supplementary Movie 5. Programmed deformation of triangular LCE lattices into a boomerang-like shape.

Supplementary Movie 6. Speed-modulated printing.

Supplementary Movie 7. Programmed surface topography.

Supplementary Movie 8. Reversibly switchable topography with a golf-ball dimple pattern.

Supplementary Movie 9. Reversibly switchable topography with a human face pattern.
